# Caring Letters Sent by a Clinician or Peer to At-Risk Veterans: A Randomized Clinical Trial

**DOI:** 10.1001/jamanetworkopen.2024.8064

**Published:** 2024-04-29

**Authors:** Mark A. Reger, Aaron Legler, MaryGrace Lauver, Kertu Tenso, Caitlin Manchester, Cameron Griffin, Kiersten L. Strombotne, Sara J. Landes, Shelan Porter, Jolie E. Bourgeois, Melissa M. Garrido

**Affiliations:** 1 Veterans Affairs Puget Sound Health Care System, Seattle, Washington; 2 Department of Psychiatry and Behavioral Sciences, University of Washington, Seattle; 3 Department of Veterans Affairs, Veterans Crisis Line, Office of Mental Health and Suicide Prevention, Veterans Affairs Central Office, Washington, DC; 4 Center for Mental Health Outcomes and Research, Central Arkansas Veterans Healthcare System, North Little Rock; 5 South Central Mental Illness Research Education and Clinical Center, Central Arkansas Veterans Healthcare System, North Little Rock; 6 Behavioral Health Quality Enhancement Research Initiative, Central Arkansas Veterans Healthcare System, North Little Rock; 7 Department of Psychiatry, University of Arkansas for Medical Sciences, Little Rock; 8 Partnered Evidence-Based Policy Resource Center, Veterans Affairs Boston Health Care System, Boston, Massachusetts; 9 Boston University School of Public Health, Boston, Massachusetts; 10 Veterans Affairs Serious Mental Illness Treatment Resource and Evaluation Center, Office of Mental Health and Suicide Prevention, Ann Arbor, Michigan

## Abstract

**Importance:**

Caring letters is an evidence-based suicide prevention intervention in acute care settings, but its outcomes among individuals who contact a national crisis line have not previously been evaluated.

**Objective:**

To examine the outcomes of the Veterans Crisis Line (VCL) caring letters intervention and determine whether there are differences in outcomes by signatory.

**Design, Setting, and Participants:**

This parallel randomized clinical trial compared signatories of caring letters and used an observational design to compare no receipt of caring letters with any caring letters receipt. Participants included veterans who contacted the VCL. Enrollment occurred between June 11, 2020, and June 10, 2021, with 1 year of follow-up. Analyses were completed between July 2022 and August 2023.

**Intervention:**

Veterans were randomized to receive 9 caring letters for 1 year from either a clinician or peer veteran signatory.

**Main Outcomes and Measures:**

The primary outcome measure was suicide attempt incidence in the 12 months following the index VCL contact. Incidence of Veterans Health Administration (VHA) inpatient, outpatient, and emergency health care use were secondary outcomes. All-cause mortality was an exploratory outcome. Wilcoxon rank-sum tests and χ^2^ tests were used to assess the differences in outcomes among the treatment and comparison groups.

**Results:**

A total of 102 709 veterans (86 942 males [84.65%]; 15 737 females [15.32%]; mean [SD] age, 53.82 [17.35] years) contacted the VCL and were randomized. No association was found among signatory and suicide attempts, secondary outcomes, or all-cause mortality. In the analysis of any receipt of caring letters, there was no evidence of an association between caring letters receipt and suicide attempt incidence. Caring letters receipt was associated with increased VHA health care use (any outpatient: hazard ratio [HR], 1.10; 95% CI, 1.08-1.13; outpatient mental health: HR, 1.19; 95% CI, 1.17-1.22; any inpatient: HR, 1.13; 95% CI, 1.08-1.18; inpatient mental health: HR, 1.14; 95% CI, 1.07-1.21). Caring letters receipt was not associated with all-cause mortality.

**Conclusions and Relevance:**

Among VHA patients who contacted the VCL, caring letters were not associated with suicide attempts, but were associated with a higher probability of health care use. No differences in outcomes were identified by signatory.

**Trial Registration:**

isrctn.org Identifier: ISRCTN27551361

## Key Points

**Question:**

Is the Veterans Crisis Line (VCL) caring letters intervention associated with reduced suicide attempts, and are there differences in outcomes by signatory?

**Findings:**

In this parallel randomized clinical trial of 102 709 veterans who contacted the VCL, caring letters were not associated with suicide attempts, but they were associated with higher probabilities of outpatient and inpatient mental health care use. No differences in outcomes were identified by signatory.

**Meaning:**

These findings suggest that caring letters may support access to care for high-risk veterans who contact the VCL.

## Teaser

This parallel randomized clinical trial compares suicide attempt incidence among veterans who received caring letters from a clinician vs a peer following a call to the Veterans Crisis Line.

## Introduction

The suicide rate for veterans in the US exceeds the rate for nonveteran adults by 57%.^1^ The US Department of Veterans Affairs (VA) established the Veterans Crisis Line (VCL) as part of a comprehensive strategy to prevent suicide^2^ among a high-risk group of veterans.^3^ While VCL contact is beneficial,^4,5^ veterans remain at high risk of suicide for up to a year after contacting the VCL. Suicide prevention interventions may be helpful after contact with the VCL, but scalable and effective approaches are needed.

The US Surgeon General’s Call to Action to Implement the National Strategy for Suicide Prevention addressed this challenge, noting the opportunity to increase the use of the caring letters suicide prevention intervention following crisis line calls.^6^ Caring letters (sometimes called caring contacts) are an evidence-based intervention in acute care settings,^7^ but, to our knowledge, they have never been tested with crisis-line callers. The intervention consists of multiple messages mailed to high-risk individuals over time, often from a clinician, to communicate that the sender cares about the recipient’s well-being.^8^ Leading theories of suicide emphasize the critical protective outcomes of feeling cared about.^9^ The caring letters are thought to increase caring connections and remind high-risk individuals that help and health care resources are available.

The VCL established a caring letters intervention for veterans who contact the crisis line. Given the limited evidence for the intervention in this new population, we conducted a randomized clinical trial, combined with a comparison of any caring letters receipt to no caring letters receipt. One of the key challenges was identifying an appropriate signatory for the letters. Many veterans do not have an established relationship with a VA clinician, and VCL responders do not have ongoing clinical relationships with veterans. We randomized eligible veterans who contacted VCL to caring letters from either a clinician or peer veteran signatory. Peer veterans were selected for testing in addition to the traditional approach of a clinician based on the unique cultural characteristics of veterans, their preference for peer support, and pilot work in the field showing the potential benefits of peer veteran caring letters.^10,11^ The study was conducted in the VA’s national integrated health care system and, to our knowledge, represents the largest evaluation of caring letters to date.

## Methods

### Trial Design and Participants

We used a mixed-methods effectiveness-implementation hybrid type 1 trial. This study design focuses primarily on evaluating the outcomes of caring letters in this context while also exploring the implementation of the intervention (eg, collection of administrative data and metrics, interviews with veterans and staff). Details of the formative and summative qualitative aspects of the evaluation have been reported elsewhere.^12,13^ This article presents the randomized evaluation of the impact of peer vs clinician letters on outcomes, as well as an observational evaluation of outcomes associated with caring letters receipt. The quantitative evaluation reported here did not include any contact with veteran participants. Methods and data ascertainment for analyses were considered nonresearch and did not require institutional review board approval per Department of Veterans Affairs Office of Research and Development Program Guide 1200.21, and informed consent was not required.^14^ This parallel randomized clinical trial followed the Consolidated Standards of Reporting Trials (CONSORT) reporting guideline. The trial protocol is available in Supplement 1.

All veterans who contacted the VCL with an identifiable address in the VHA’s Corporate Data Warehouse (CDW) and who contacted the crisis line between June 11, 2020, and June 10, 2021, were screened for inclusion in the caring letters cohort (Figure). Friends and family calling on behalf of a veteran, civilians, and callers who died prior to randomization were excluded. Individuals who recontacted the VCL after enrollment were flagged so that they were not re-enrolled.

**Figure.**
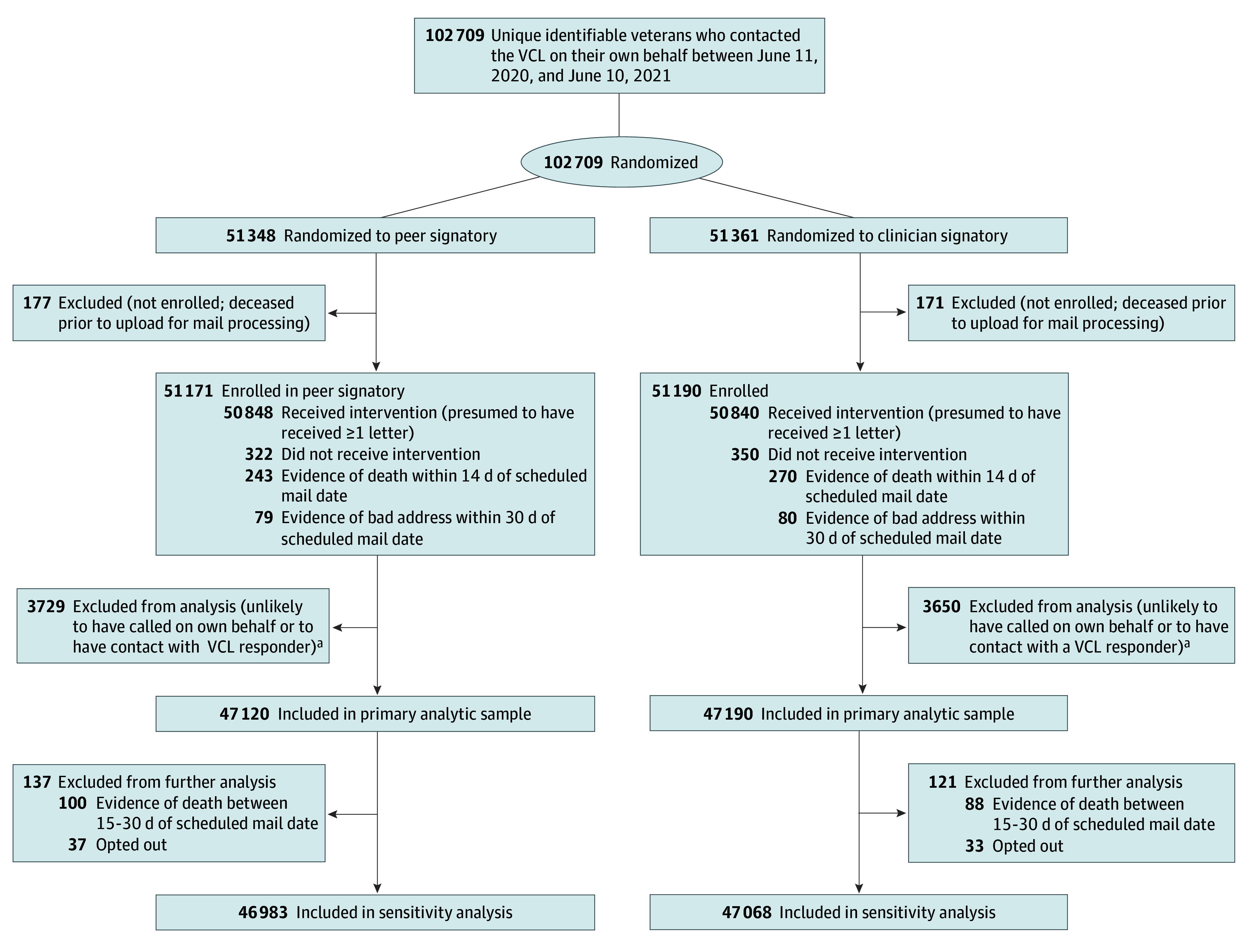
CONSORT Diagram of Peer-Clinician Evaluation ^a^Individuals who were unlikely to have called on their own behalf or to have contact with a Veterans Crisis Line (VCL) responder were identified as those for whom there was a record that the call from a third party caller, whose call record corresponded to an outgoing call from a responder that did not end with contact with the veteran, and whose call source was listed as an email referral, compassionate care, caregiver, social media, or assigned callback.

We compared members of the eligible cohort with a second cohort that included individuals who would have been eligible for caring letters had the intervention been available before June 2020 or if they had provided enough identifiable information during their VCL call (eg, name, phone number, and/or social security number) (eFigure 1 in Supplement 1). For this comparison, we restricted the sample to veterans who had at least 1 VHA encounter in the 24 months before the index call.

### Randomization

Many veterans who contact the VCL do not have an established relationship with a VA clinician, and VCL responders do not have ongoing clinical relationships with veterans. Since it was unclear who should send the caring letters, we randomly assigned veterans to receive caring letters from either a clinician or a peer veteran, both of whom work with the VCL but whom the veterans have not met. The clinician was identified as a counselor, and the letters included the title Dr.

Randomization to the peer and clinician conditions was conducted using permuted block randomization (block size = 4), stratified by sex. New callers were identified and randomized weekly by the senior statistician. Eligible callers were enrolled in the study unless they requested to opt out of the intervention.

### Intervention

A total of 9 caring letters in the form of flat cards were mailed to eligible participants in colorful greeting card envelopes. The letters included caring messages, well wishes, and a 1-800 VA phone number for questions regarding clinical needs, the caring letters, or to opt out of the intervention. The content of the clinician and peer veteran cards was similar, but the language and style were updated to reflect cultural norms. Examples of the cards and their content have been published elsewhere.^12^ One letter was sent on Veterans Day, based on veteran preferences,^15^ and 8 additional letters were mailed to each veteran 1, 2, 3, 4, 6, 8, 10, and 12 months after the initial VCL contact date, similar to previous trials.^16,17^ Every envelope also included a resource card that listed the VCL number and online VHA mental health resources.

### Data Sources and Outcomes

Data were obtained from the VHA CDW and Vital Status File, VCL operational databases, and VHA Office of Mental Health and Suicide Prevention surveillance data. Our primary outcome was suicide attempts, which were ascertained from the *International Statistical Classification of Diseases and Related Health Problems, Tenth Revision *(*ICD-10*) codes for suicide attempt and intentional self-harm and indicators for suicide attempt, suicidal self-directed violence, and undetermined self-directed violence from the VA’s required active surveillance program.^18^ Secondary outcomes were VHA health care use (incidence of any outpatient, mental health outpatient, any inpatient, mental health inpatient, and emergency department [ED] use). We also assessed the number of VHA visits. Examination of all-cause mortality was an exploratory aim. Outcomes were tracked for 12 months from the date of the VCL index contact. The widths of 95% CIs have not been adjusted for multiplicity and may not be used in place of hypothesis testing for secondary outcomes.

In addition to measuring intervention receipt, we controlled for age, sex, race, ethnicity, marital status, age at separation from service, discharge status, military branch, incidence of past-year suicide attempt, incidence of past-year inpatient or outpatient mental health visit, incidence of Elixhauser comorbidities^19^ in the past 2 years, and VA medical facility. Race and ethnicity were based on values included in the electronic health record. Race categories included American Indian or Alaska Native, Asian, Black, Native Hawaiian or Other Pacific Islander, White, more than 1 race, and race unknown. Ethnicity was measured as Hispanic, not Hispanic, or unknown. In our analyses of the impact of receiving any letters, we also controlled for the year and the month of call, and whether the call happened after the beginning of the COVID-19 pandemic (indicator for the call occurring in March 2020 or later). In the signatory analysis, 2 individuals were missing data on age and 1646 (1.7%) were missing data on age at separation (29 were also missing data on sex). In the letter receipt analysis, 1893 (0.8%) were missing data on age at separation. We used complete case analysis because the number of individuals with missing data on age at separation was balanced across treatment groups in both analyses.

### Statistical Analysis

The methods include 2 analytic approaches to address the aims: (1) a randomized study of clinician vs peer caring letters and (2) a cohort study with a historical control of receipt vs nonreceipt of the intervention. Analyses were completed between July 2022 and August 2023. Statistical significance was set at *P* < .05, and all tests were 2-sided. The software package Stata version 17 (StataCorp) was used.

#### Peer vs Clinician Signatory

The trial was powered to detect a relative difference of 10.8% in suicide attempt incidence across signatory conditions. Additional details are available in the eAppendix in Supplement 1. We assessed the balance of covariates by signatory with standardized differences, considering a value of 0.1 or less to indicate adequate balance. We conducted χ^2^ and Wilcoxon rank-sum tests of differences in outcomes in the 2 signatory groups, as well as logistic regression models controlling for the covariates noted above. The primary analysis focused on 94 310 individuals who were randomized to either a peer or clinician-signed letter who were presumed to receive at least 1 letter (no evidence of death within 14 days of the scheduled mail date [513 individuals] or evidence of an incomplete or inaccurate address within 30 days of the scheduled mail date [159 individuals]) (Figure). In sensitivity analyses, we further excluded individuals with evidence of death between 15 to 30 days of the scheduled mail date (188 individuals) and those who opted out of the intervention (70 individuals).

#### Receipt of Any Letters vs No Letters

The analysis was powered to detect a relative difference of 9.1% in suicide attempt incidence among individuals receiving and not receiving letters (eAppendix in Supplement 1). Our main analysis in the cohort study included individuals enrolled in the intervention or who would have been enrolled in the intervention had it been available earlier, regardless of whether there was evidence of death or a bad address immediately after the index call. We assessed the balance of covariates across treatment and comparison groups with standardized differences and conducted χ^2^ tests and Wilcoxon rank-sum tests of differences in outcomes in the treatment and comparison groups. We considered a difference-in-differences strategy to isolate changes in outcomes due to receipt of caring letters from changes in outcomes due to calls during the COVID-19 pandemic^12^ (Statistical Analysis Plan in Supplement 2), but the parallel trends assumption was not supported. Instead, we ran individual-level time-to-event analyses for each binary outcome, controlling for the variables noted above, including whether the call occurred in March 2020 or later. Individuals could be included in both the treatment and comparison cohorts. We treated the index call for the treatment group as a censoring event for the comparison observation and used patient-level clustered standard errors. In analyses of suicide attempts and health care use, death was treated as a censoring event. Visual inspection of time to events across treatment and comparison individuals support the proportional hazards assumption for suicide attempt and utilization analyses (eFigure 2 in Supplement 1).

In the sensitivity analyses, we restricted the sample to those with no evidence of an invalid address in the 365 days following the index call (ie, presumed completers). We also explored the sensitivity of results in a sample including 1 index call per individual for callers who had at least 1 call in the 12 months prior to the index call (ie, repeat callers).

## Results

### Peer vs Clinician Signatory

The primary analytic sample for the signatory comparison included 94 310 individuals (47 120 peer signatory [49.96%]; 47 190 clinician signatory [50.03%]; 86 942 males [84.65%], 15 737 females [15.32%]; mean [SD] age, 53.82 [17.35] years). All characteristics were well-balanced across signatory groups (Table 1 and eTable 3 in Supplement 1). We did not observe a significant association between signatory and our primary outcome in either unadjusted or adjusted analyses (Table 2 and eTable 4 and 5 in Supplement 1). Of the sample, 3489 peer signatory recipients (7.4%) and 3567 clinician signatory recipients (7.6%) had a suicide attempt within 12 months of the index VCL call. Peer and clinician signatory recipients had similar rates of secondary outcomes, including mortality (3.8% of peer and 3.7% of clinician recipients died within 12 months of the index call) and health care use (eg, 69.0% of peer recipients and 68.5% of clinician recipients had at least 1 outpatient mental health visit in the 12 months following the index call). Results were robust to changes in exclusion criteria (eTable 6 in Supplement 1). There were similar low rates of those who opted out in both signatory conditions (37 opted out from the peer group; 33 opted out from the clinician group).

**Table 1.** Characteristics of Veterans Receiving Caring Letters With Peer vs Clinician Signatories^a^

Characteristics	Participants, No. (%)		Absolute value of standardized difference
	Peer (n = 47 120)	Clinician (n = 47 190)	
Age, mean (SD), y^b^	53.77 (17.31)	53.80 (17.32)	0.002
Age at separation, mean (SD), y^c^	26.24 (6.69)	26.23 (6.68)	0.003
Sex^d^			
Female	7456 (15.82)	7375 (15.63)	0.005
Male	39 649 (84.14)	39 801 (84.34)	
Race			0.001
American Indian or Alaska Native	507 (1.08)	527 (1.12)	
Asian	542 (1.15)	562 (1.19)	
Black	12 271 (26.04)	12 224 (25.90)	
Native Hawaiian or Other Pacific Islander	473 (1.00)	497 (1.05)	
White	27 939 (59.29)	28 010 (59.36)	
More than one race	726 (1.54)	708 (1.50)	
Race unknown	4662 (9.89)	4662 (9.88)	
Hispanic ethnicity	3744 (7.95)	3646 (7.73)	0.002
Married	16 822 (35.70)	16 957 (35.93)	0.003
Branch of service			
Air Force	5999 (12.73)	5948 (12.60)	0.002
Army	24 405 (51.79)	24 497 (51.91)	
Navy	9240 (19.61)	9254 (19.61)	
Marine Corps	6131 (13.01)	6131 (12.99)	
Other and missing	1345 (2.85)	1360 (2.88)	
Discharge type			0.003
Honorable	41 946 (89.02)	42 051 (89.11)	
Dishonorable	378 (0.80)	376 (0.80)	
Other	3543 (7.52)	3534 (7.49)	
Unknown	1253 (2.66)	1229 (2.60)	
Past-year suicide attempt	3006 (6.38)	2978 (6.31)	0.003
Past-year inpatient mental health encounter	3205 (6.80)	3222 (6.83)	0.001
Past-year outpatient mental health encounter	25 489 (54.09)	25 576 (54.20)	0.002

^a^ Rates of Elixhauser comorbidities are also balanced across signatory groups. Full results are available in eTable 3 in Supplement 1.

^b^ Missing for 2 individuals.

^c^ Missing for 1646 individuals.

^d^ Missing for 29 individuals.

**Table 2.** Recipients of Caring Letters Signed by Peers and Clinicians Had Similar Rates of Outcomes

Outcome	Individuals experiencing outcome, No. (%)		Average incremental effect of clinician signatory (95% CI)^a^
	Peer group	Clinician group	
Any suicide attempt	3489 (7.40)	3567 (7.56)	0.001 (−0.002 to 0.005)
All-cause mortality	1777 (3.77)	1751 (3.71)	−0.001 (−0.003 to 0.002)
Any outpatient use	43 725 (92.79)	43 833 (92.89)	0.001 (−0.002 to 0.003)
Any outpatient mental health care use	32 494 (68.96)	32 336 (68.52)	−0.004 (−0.009 to 0.001)
Any inpatient use	9555 (20.28)	9629 (20.40)	0.001 (−0.003 to 0.006)
Any inpatient mental care health use	4855 (10.30)	4939 (10.47)	0.001 (−0.003 to 0.005)
Any emergency department use	17 653 (37.46)	17 574 (37.24)	−0.002 (−0.008 to 0.004)

^a^ From logistic regression of outcomes on clinician signatory, sociodemographic and clinical characteristics, and station fixed effects. Average incremental effect represents the mean percentage point difference in estimated likelihood of outcome if one were to change from receiving a peer signatory letter to receiving a clinician signatory letter, holding all other variables constant at their original values. Full model output is available in the eAppendix 1 in Supplement 1.

### Receipt of Any Letters vs No Letters

The primary sample included 227 502 observations (203 636 unique callers), with 87 926 (38.65%) who were mailed caring letters and 139 576 (61.35%) who were not mailed caring letters. There were 23 866 individuals represented in both the treatment and comparison groups. Treatment and comparison individuals had similar sociodemographic and clinical characteristics (Table 3 and eTable 7 in Supplement 1), with standardized differences of less than 0.1 for all covariates except for past-year outpatient mental health visits (58.5% of treatment and 64.9% of comparison individuals; standardized difference, 0.13). There was no evidence of an association between caring letter receipt and suicide attempt incidence in either our unadjusted or adjusted analyses (eTables 8 and 9 in Supplement 1). In our sample, 6801 caring letter recipients (7.7%) and 10 910 nonrecipients (7.8%) had a suicide attempt in the 12 months following the index call. In adjusted survival analyses, we did not find evidence of an association between receipt of caring letters and mortality. However, veterans who received caring letters were significantly more likely to have subsequent inpatient care (any inpatient: hazard ratio [HR], 1.13; 95% CI, 1.08-1.18), inpatient mental health care (HR, 1.14; 95% CI, 1.07-1.21), outpatient care (HR, 1.10; 95% CI, 1.08-1.13), outpatient mental health care (HR, 1.19; 95% CI, 1.17-1.22), and ED visits (HR, 1.11; 95% CI, 1.07-1.14) (*P* < .001) (Table 4 and eTable 9 in Supplement 1). Similar patterns were observed when the sample was restricted to those presumed to have received all caring letters and when we examined repeat callers only (eTables 10 and 11 in Supplement 1).

**Table 3.** Characteristics of Veterans Who Did and Did Not Receive Caring Letters^a^

Variable	Individuals, No. (%)		Absolute value of standardized difference
	No caring letters (n = 139 576)	Caring letters (n = 87 926)	
Age, mean (SD), y	53.12 (16.81)	54.44 (17.20)	0.077
Age at separation, mean (SD)^b^	25.97 (6.70)	26.25 (6.69)	0.041
Sex			
Female	21 644 (15.51)	13 922 (15.83)	0.009
Male	117 932 (84.49)	74 004 (84.17)	
Race			
American Indian or Alaska Native	1625 (1.16)	977 (1.11)	0.004
Asian	1465 (1.05)	1026 (1.17)	
Black	37 358 (26.77)	23 356 (26.56)	
Native Hawaiian or Other Pacific Islander	1388 (0.99)	919 (1.05)	
White	85 262 (61.09)	53 018 (60.30)	
More than one race	1922 (1.38)	1331 (1.51)	
Race unknown	10 556 (7.56)	7299 (8.30)	
Hispanic ethnicity	10 745 (7.70)	6980 (7.94)	0.027
Married	49 804 (35.68)	31 837 (36.21)	0.015
Branch of service			
Air Force	17 321 (12.41)	11 285 (12.83)	0
Army	74 551 (53.41)	46 307 (52.67)	
Navy	27 129 (19.44)	17 290 (19.66)	
Marine Corps	17 867 (12.80)	11 338 (12.89)	
Other and missing	2708 (1.94)	1706 (1.94)	
Discharge type			0.004
Honorable	126 070 (90.32)	79 548 (90.47)	
Dishonorable	974 (0.70)	543 (0.62)	
Other	10 115 (7.25)	6326 (7.19)	
Unknown	2417 (1.73)	1509 (1.72)	
Past-year suicide attempt	8925 (6.39)	5805 (6.60)	0.008
Past-year inpatient mental health encounter	12 443 (8.91)	6489 (7.38)	0.056
Past-year outpatient mental health encounter	90 593 (64.91)	51 398 (58.46)	0.133

^a^ Rates of Elixhauser comorbidities are also balanced across signatory groups. Full results are available in eTable 5 in Supplement 1.

^b^ Missing for 1893 individuals.

**Table 4.** Outcomes Among Individuals Receiving and Not Receiving Caring Letters

Outcome	Individuals experiencing outcome, No. (%)		Hazard ratio of caring letter receipt (95% CI)^a^
	No letters (comparison) group	Letters (treatment) group	
Any suicide attempt	10 910 (7.81)	6801 (7.73)	1.02 (0.95-1.09)
All-cause mortality	5607 (4.02)	3870 (4.40)	1.07 (0.96-1.18)
Any outpatient use	132 777 (95.13)	84 318 (95.90)	1.10 (1.08-1.13)
Any outpatient mental health care use	104 816 (75.10)	62 375 (70.94)	1.19 (1.17-1.22)
Any inpatient use	32 173 (23.05)	18 886 (21.48)	1.13 (1.08-1.18)
Any inpatient mental care health use	17 042 (12.21)	9548 (10.86)	1.14 (1.07-1.21)
Any emergency department use	56 183 (40.25)	34 552 (39.30)	1.11 (1.07-1.14)

^a^ From time to event analyses of outcomes on caring letter receipt, sociodemographic and clinical characteristics, and station fixed effects. Cause-specific hazard ratios are presented for outcomes other than mortality. Full model output is available in the eAppendix in Supplement 1.

## Discussion

Among a national sample of veterans who contacted the VCL, we did not find evidence that caring letters were associated with a reduction in suicide attempts, but they were associated with a higher probability of VHA health care utilization. Our results did not support a reduction in all-cause mortality. There were no differences in outcomes by signatory.

Prior research on the association of caring letters and other caring contacts (eg, text messages) with suicide attempts has been mixed.^16,20,21^ A recent meta-analysis found a protective association for suicide attempts at 1 year, but not 2 years, postrandomization to caring contacts.^22^ The current trial was much larger than any prior study and scaled up the intervention in novel ways; veterans received caring letters from someone they had never met. It is possible that this approach reduced the impact of the intervention since prior trials used messages from someone the patients knew. Future analyses could examine whether certain groups of veterans are more likely to benefit from caring letters (eg, callers with higher risk vs callers with lower risk).

Caring letters were associated with higher probability of VHA inpatient and outpatient health care utilization. In the current trial, the intervention included a resource card with information that could be used to connect to care. In qualitative interviews with veterans who received the VCL intervention, some reported using the resource card to connect with mental health care or discussed how the cards helped them stay engaged in care.^13^ Others reported improved perspectives of the VA, and many reported keeping the resource card or letters. Potential mechanisms of increased health care utilization require additional study. Prior researchers have speculated that caring letters may increase patient willingness to engage in care,^22^ but examinations of ED visits or hospitalizations after receipt of caring contacts have been inconclusive.^22^ We hypothesized that the intervention would be associated with higher rates of outpatient mental health care utilization and thereby decrease inpatient mental health care needs. Our findings that both inpatient and outpatient mental health care rates were higher among caring letter recipients are equally intuitive since higher rates of outpatient care may identify more acute care needs. A primary goal of the VHA is to improve veteran access to high-quality care, especially in mental health settings.^23^ Veterans who contact the VCL are a high-risk group who often have complex mental health needs. While this study did not examine the benefits of increased health care utilization, future research could explore the potential advantages of increased treatment.

We did not observe differences in outcomes by signatory. In qualitative interviews with veterans, many could not recall who sent the letters, but they described in detail how the cards made them feel.^13^ The characteristics of our intervention may have contributed to these findings since the veterans did not know the sender personally. This possibility requires additional study.

All-cause mortality was recommended as a potentially useful outcome in a prior systematic review of caring contacts since it improves power compared with suicide mortality.^22^ However, all-cause mortality includes many causes of death that we would not expect caring contacts to affect, and our results did not support an association with the intervention. Exploratory analyses (due to low power) for suicide mortality are planned when the cause of death data become available in the future.

### Limitations

This study has limitations, particularly due to the start of the COVID-19 pandemic. While we randomized to signatory, we relied on an observational comparison with a historical cohort to estimate the impact of receiving caring letters. The pandemic started only a few months before the launch of the trial. Our estimates may be biased to the extent the pandemic affected both the likelihood of contacting the VCL^24^ and our outcomes. Although we could not use our planned difference-in-differences strategy to account for the effect of the COVID-19 pandemic, the analyses presented here adjust for the impact of secular trends and the pandemic on outcomes. Future work should compare outcomes among individuals who received caring letters after the height of the COVID-19 pandemic with individuals who contacted VCL before caring letters were offered. In addition, the use of health care records to ascertain suicide attempts is a common method,^25,26^ but may underestimate attempts. However, this study had the advantage of access to VHA suicide attempt surveillance data, which has been shown to improve ascertainment^27^ relative to the use of *ICD-10* codes alone. Moreover, since the intervention group was more likely to receive subsequent VHA care, suicide attempts may have been identified at a higher rate among caring letter recipients, making it less likely we would detect an effect of the intervention. In addition, the mechanisms associated with higher health care use are unknown. Despite these limitations, these results provide the first evidence of the potential impacts of caring letters for veterans contacting the VCL.

## Conclusions

In this study, caring letters were not associated with suicide attempts or all-cause mortality, but they were associated with higher probabilities of outpatient and inpatient mental health care use, ED visits, and any outpatient or inpatient VA care use. No differences in outcomes were identified when cards were sent from a peer veteran vs a clinician the recipient had not met. These results will be used by the VCL to optimize the caring letters intervention.

## Notes

Supplement 1. **eAppendix. **Description of Power Analyses **eFigure 1**. CONSORT Diagram, Receipt of Any Letters vs No Letters Evaluation **eFigure 2.** Visual Inspection of Proportional Hazards Assumptions **eTable 1.** Power to Detect Differences in Suicide Attempt Incidence, Suicide Mortality, and VHA Mental Health Encounters in Pre-Post Comparison of Caring Contacts **eTable 2.** Power to Detect Differences in Suicide Attempt Incidence, Suicide Mortality, and VHA Mental Health Encounters in Randomized Comparison of Two Versions of Caring Contacts **eTable 3.** Descriptive Statistics by Caring Letters Signatory **eTable 4.** Rates of All Outcomes for Peer-Provider Signatory Comparison **eTable 5.** Average Incremental Effects From Logistic Regressions of Association Between Outcomes and Caring Letters Signatory **eTable 6.** Average Incremental Effects From Logistic Regressions of Association Between Outcomes and Caring Letters Signatory in Sensitivity Analysis **eTable 7.** Descriptive Statistics by Caring Letters Receipt **eTable 8.** Rates of All Outcomes for Letters vs No Letters Comparison **eTable 9.** Hazard Ratios From Time-to-Event Analyses of Association Between Outcomes and Caring Letters Receipt, Full Sample **eTable 10.** Hazard Ratios From Time-to-Event Analyses of Association Between Outcomes and Caring Letters Receipt, Presumed Completers **eTable 11.** Hazard Ratios From Time-to-Event Analyses of Association Between Outcomes and Caring Letters Receipt, Repeat Callers
eReferences.

Supplement 2. **eAppendix 1.** Trial Protocol **eAppendix 2. **Statistical Analysis Plan

Supplement 3.
Data Sharing Statement
