# Supplementary material for: Caring Letters Sent by a Clinician or Peer to At-Risk Veterans: A Randomized Clinical Trial
Source: JAMA Netw Open. 2024 Apr 29;7(4):e248064. doi: 10.1001/jamanetworkopen.2024.8064 (PMC11059042; doi:10.1001/jamanetworkopen.2024.8064)
Supplement: Supplement 1. — eAppendix. Description of Power Analyses eFigure 1. CONSORT Diagram, Receipt of Any Letters vs No Letters Evaluation eFigure 2. Visual Inspection of Proportional Hazards Assumptions eTable 1. Power to Detect Differences in Suicide Attempt Incidence, Suicide Mortality, and VHA Mental Health Encounters in Pre-Post Comparison of Caring Contacts eTable 2. Power to Detect Differences in Suicide Attempt Incidence, Suicide Mortality, and VHA Mental Health Encounters in Randomized Comparison of Two Versions of Caring Contacts eTable 3. Descriptive Statistics by Caring Letters Signatory eTable 4. Rates of All Outcomes for Peer-Provider Signatory Comparison eTable 5. Average Incremental Effects From Logistic Regressions of Association Between Outcomes and Caring Letters Signatory eTable 6. Average Incremental Effects From Logistic Regressions of Association Between Outcomes and Caring Letters Signatory in Sensitivity Analysis eTable 7. Descriptive Statistics by Caring Letters Receipt eTable 8. Rates of All Outcomes for Letters vs No Letters Comparison eTable 9. Hazard Ratios From Time-to-Event Analyses of Association Between Outcomes and Caring Letters Receipt, Full Sample eTable 10. Hazard Ratios From Time-to-Event Analyses of Association Between Outcomes and Caring Letters Receipt, Presumed Completers eTable 11. Hazard Ratios From Time-to-Event Analyses of Association Between Outcomes and Caring Letters Receipt, Repeat Callers eReferences. [file jamanetwopen-e248064-s001.pdf]

Supplemental Online Content

Reger MA, Legler A, Lauver M. Caring letters sent by a clinician or peer to at-risk veterans: a randomized clinical trial. *JAMA Netw Open*. 2024;7(4):e248064.
doi:10.1001/jamanetworkopen.2024.8064

eAppendix. Description of Power Analyses

eFigure 1. CONSORT Diagram, Receipt of Any Letters vs No Letters Evaluation

eFigure 2. Visual Inspection of Proportional Hazards Assumptions

eTable 1. Power to Detect Differences in Suicide Attempt Incidence, Suicide Mortality, and VHA Mental Health Encounters in Pre-Post Comparison of Caring Contacts

eTable 2. Power to Detect Differences in Suicide Attempt Incidence, Suicide Mortality, and VHA Mental Health Encounters in Randomized Comparison of Two Versions of Caring Contacts

eTable 3. Descriptive Statistics by Caring Letters Signatory

eTable 4. Rates of All Outcomes for Peer-Provider Signatory Comparison

eTable 5. Average Incremental Effects From Logistic Regressions of Association Between Outcomes and Caring Letters Signatory

eTable 6. Average Incremental Effects From Logistic Regressions of Association Between Outcomes and Caring Letters Signatory in Sensitivity Analysis

eTable 7. Descriptive Statistics by Caring Letters Receipt

eTable 8. Rates of All Outcomes for Letters vs No Letters Comparison

eTable 9. Hazard Ratios From Time-to-Event Analyses of Association Between Outcomes and Caring Letters Receipt, Full Sample

eTable 10. Hazard Ratios From Time-to-Event Analyses of Association Between Outcomes and Caring Letters Receipt, Presumed Completers

eTable 11. Hazard Ratios From Time-to-Event Analyses of Association Between Outcomes and Caring Letters Receipt, Repeat Callers

eReferences.

This supplemental material has been provided by the authors to give readers additional information about their work.

Description of Power Analyses

If the sample included 90,000 VHA enrollees who call the VCL, the study would be powered to detect differences in suicide attempt incidence and engagement with VHA mental health care in both pre-post comparisons and in comparisons of two versions of caring contacts.

We assume that there are 90,000 VHA enrollees who call the VCL and who may receive caring contacts over a 1-year period. We assume we can identify 40,000 VHA enrollees who called the VCL in a one-year period before the caring contacts intervention began. Baseline estimates are based on previous analyses of VCL data (eTables 1 and 2).[1] We have 80% power to detect differences in outcomes listed in eTable 1 in a pre-post comparison of caring contacts when $\alpha = .05$. We have 80% power to detect differences in outcomes listed in eTable 2 across two versions of caring contacts when $\alpha = .05$.

© 2024 Reger MA et al. *JAMA Network Open.*

eFigure 1. CONSORT Diagram, Receipt of Any Letters vs No Letters Evaluation

Unique VCL Calls by identifiable Veterans
6/11/2018 to 6/10/2021
385,646 Calls[a]; 235,768 Unique Callers

Primary Analytic Cohort – Received Letters (Treatment)

N = 102,709 Calls, 102,709 Veterans

Exclusion[b]: 14,728 Calls, 14,728 Veterans
- Call from 3rd party caller (n = 3,396 Calls)
- Outgoing call that did not include Veteran contact (n = 5,323 Calls)
- Call source listed as email referral, compassionate care, caregiver, Facebook, or assigned callback (n = 746 Calls)
- No inpatient or outpatient visit within 24 months (n = 7,669 Calls)

N = 87,981 Calls, 87,981 Veterans
Exclusion: Death date recorded before VCL call date (n = 55)
Primary Analytic Cohort Treatment Group:
N = 87,926 Calls

Primary Analytic Cohort - No Letters (Comparison)

N = 282,937 Calls, 159,021 Veterans

Exclusion[b]: 26,384 Calls, 23,167 Veterans
- Call from 3rd party caller (n = 8,959 Calls)
- Outgoing call that did not include Veteran contact (n = 1,528 Calls)
- Call source listed as email referral, compassionate care, caregiver, Facebook, or assigned callback (n = 1,329 Calls)
- No inpatient or outpatient visit within 24 months (n = 16,197 Calls)

N = 256,553 Calls, 139,680 Veterans

Randomly select 1 Call Per Veteran
Exclusion: Death date recorded before VCL call date (n = 104)
Primary Analytic Cohort Comparison Group:
N = 139,576 Calls

Sub Analysis 1 (SA1): Presumed Completer Analysis (Treatment)

Primary Analytic Cohort N = 87,926
Exclusion: N = 6,989
- Evidence of death within 30 days of expected mail date (n=765)
- Missing/Bad Address on 1st Letter (n = 107)
- Opted out (n=69)
- Within 1 year of VCL Call Date:
  - Flagged Do Not Send prior to Death Date (n=375)
  - Flagged Do Not Send with no evidence of Death. (n=6,157)

SA1 Treatment Group: n=80,992

Sub Analysis 1 (SA1): Presumed Completer Analysis (Comparison)

Primary Analytic Cohort N = 139,576
Exclusion: N = 1,151
- Evidence of death within 30 days of expected mail date (n=1,151)
- Missing/Bad Address on 1st Letter (n = 0)
- Opted out (n=0)
- Within 1 year of VCL Call Date:
  - Flagged Do Not Send prior to Death Date (n=0)
  - Flagged Do Not Send with no evidence of Death. (n=0)

SA1 Comparison Group: n = 138,529

Sub Analysis 2: Repeat Callers

Total N = 344,532 Calls; 227,650 Veterans
Treatment: N = 87,981 Calls, 87,981 Veterans
Comparison: N = 256,553 Calls, 139,680 Veterans
- Exclusion: No Call within 1 year Prior to VCL Call Date
  - Treatment (n = 72,316 Calls, 72,316 Veterans)
  - Comparison (n=132,956 Calls, 129,725 Veterans)

Exclusion: Death date recorded before VCL call date (n = 15)
Randomly Select 1 Call per Veteran

SA2 Treatment Group: N = 9,579 Calls, 9,579 Veterans

SA2 Comparison Group: SA2 N = 35,533 Calls, 35,533 Veterans

a) To create the pool of eligible calls for the comparison group, we dropped comparison calls after the index caring letters date for Veterans who are in the treatment group.
b) Exclusion categories not mutually exclusive.

© 2024 Reger MA et al. *JAMA Network Open.*

eFigure 2. Visual Inspection of Proportional Hazards Assumptions

Visual inspection of time to events across treatment and comparison individuals support the proportional hazards assumption in our analyses of the association of receipt of letters with suicide attempt and utilization outcomes.

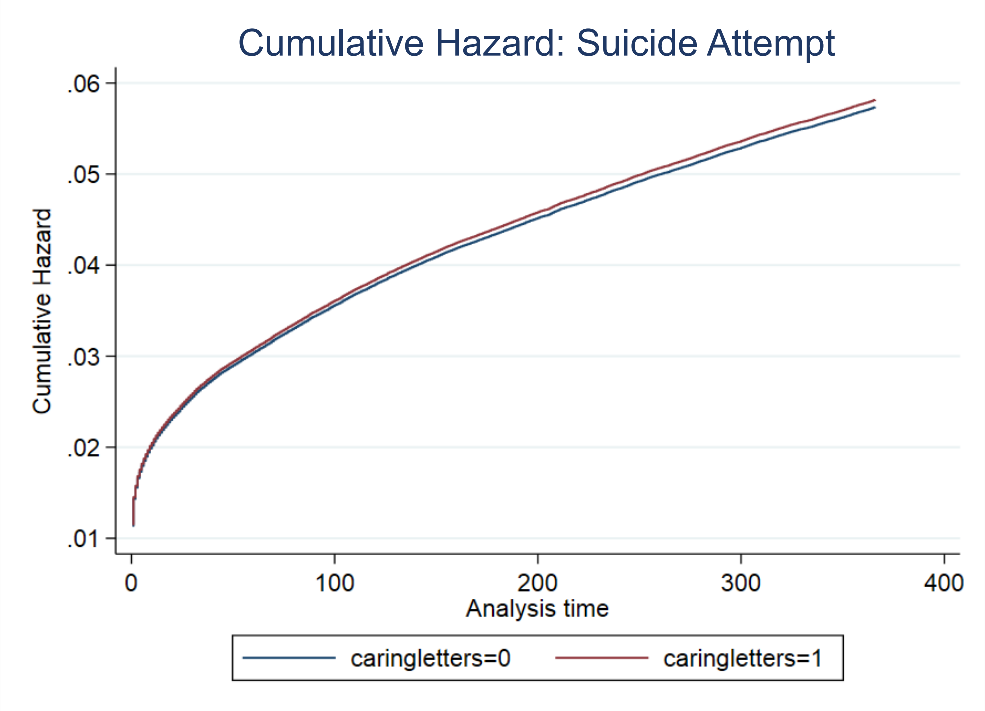

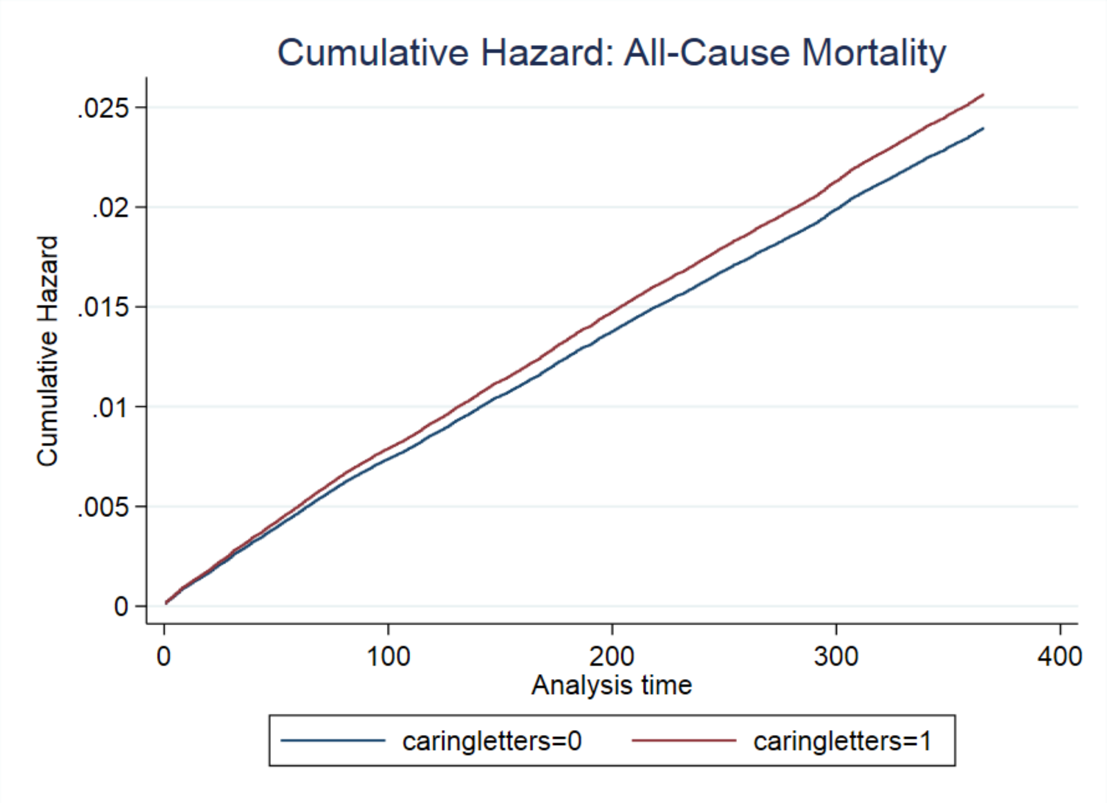

© 2024 Reger MA et al. *JAMA Network Open.*

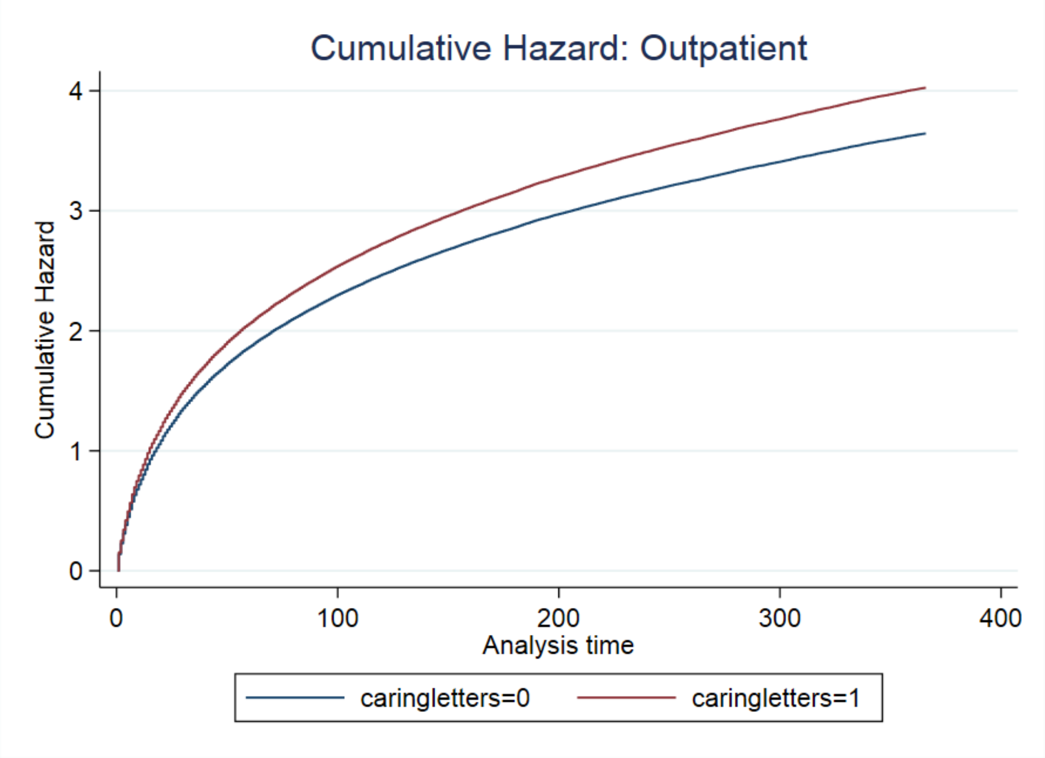

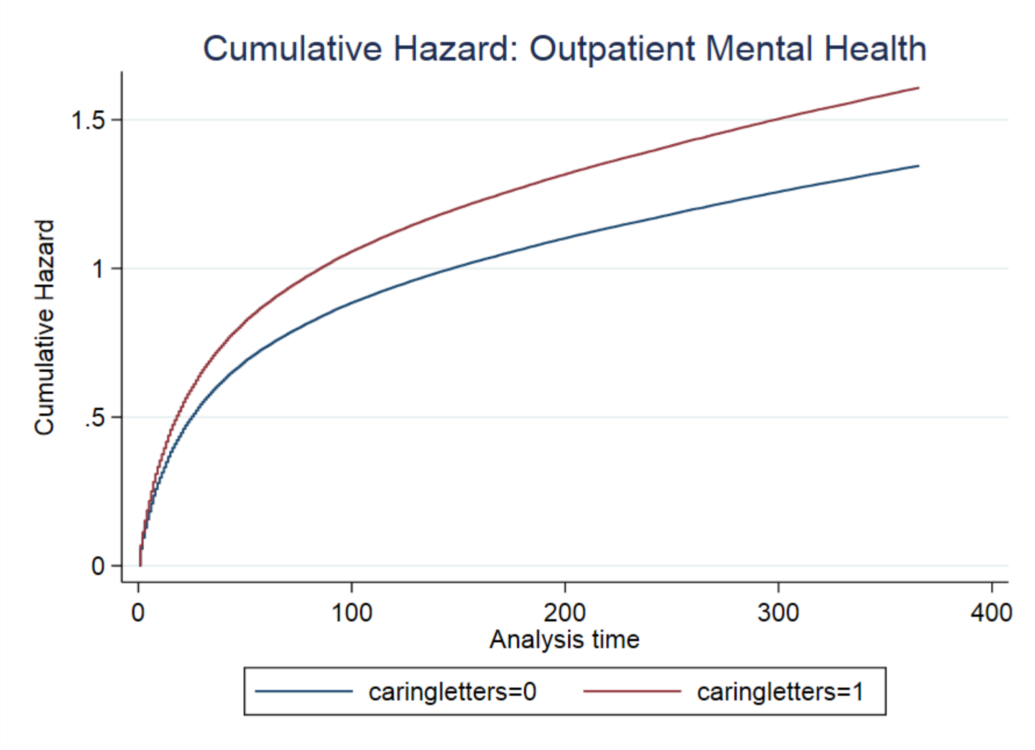

© 2024 Reger MA et al. *JAMA Network Open.*

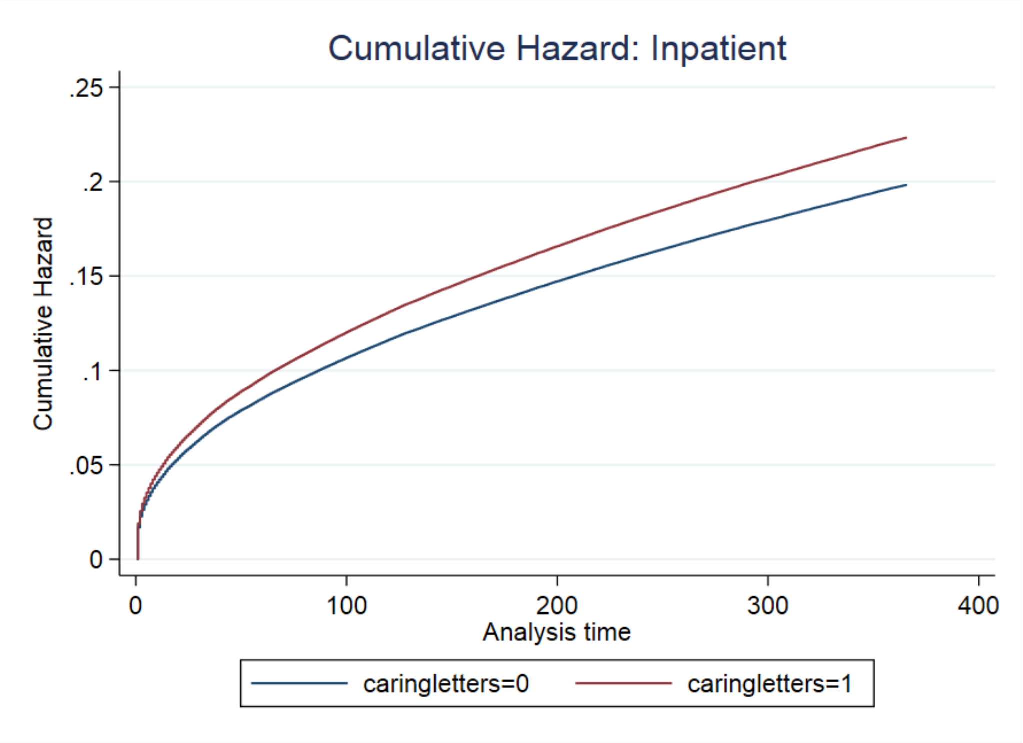

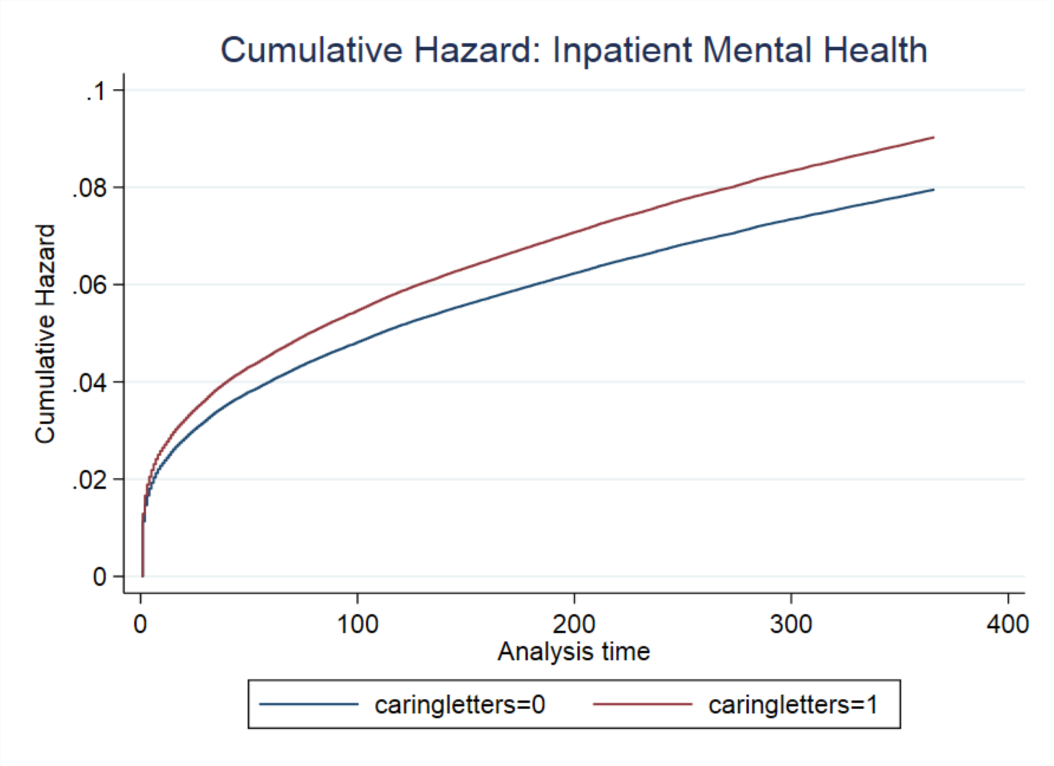

© 2024 Reger MA et al. *JAMA Network Open.*

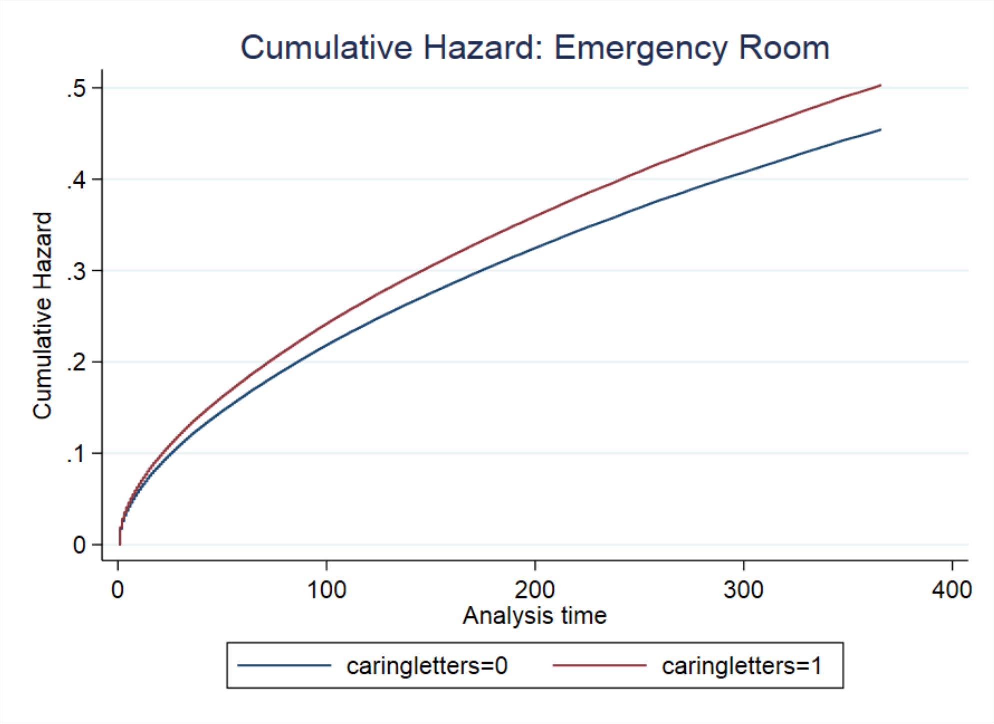

© 2024 Reger MA et al. *JAMA Network Open.*

eTable 1. Power to Detect Differences in Suicide Attempt Incidence, Suicide Mortality, and VHA Mental Health Encounters in Pre-Post Comparison of Caring Contacts

Outcome	Relative difference	Absolute difference
Suicide attempt incidence (rate per 100,000 person-years)	9.1%	Reduction from 3172 to 2883 per 100,000 person-years
Suicide mortality (rate per 100,000 person-years, entire sample)	28.1%	Reduction from 301 to 216 per 100,000 person-years
VHA mental health encounter incidence	1.2%	Increase from 68.6% to 69.4% of callers with an encounter in 12-month period

eTable 2. Power to Detect Differences in Suicide Attempt Incidence, Suicide Mortality, and VHA Mental Health Encounters in Randomized Comparison of Two Versions of Caring Contacts

Outcome	Relative difference	Absolute difference
Suicide attempt incidence (rate per 100,000 person-years)	10.8%	Reduction from 2760 to 2462 per 100,000 person-years[a]
Suicide mortality (rate per 100,000 person-years, entire sample)	33.0%	Reduction from 271 to 182 per 100,000 person-years[b]
VHA mental health encounter incidence	1.06%	Increase from 75.5% to 76.3% of callers with an encounter in 12 month period[c]

a) We assume that each arm has a 13% decrease in suicide attempt incidence relative to baseline.[2]
b) We assume that each arm has a 10% decrease in suicide attempt incidence relative to baseline
c) We assume that each arm has a 10% increase in encounter incidence relative to baseline

© 2024 Reger MA et al. *JAMA Network Open.*

eTable 3. Descriptive Statistics by Caring letters Signatory

	Peer (N=47,120)		Provider (N=47,190)		Total (N=94,310)		Std. diff.
	N or Mean	% or SD	N or Mean	% or SD	N or Mean	% or SD	
Age in years	53.77	(17.31)	53.80	(17.32)	53.78	(17.32)	-0.002
Age at separation	26.24	(6.69)	26.23	(6.68)	26.23	(6.68)	0.003
Age group							0.000
Less than 40	12,924	(27.43)	12,930	(27.40)	25,854	(27.41)	
40-54	10,294	(21.85)	10,397	(22.03)	20,691	(21.94)	
55-64	9,644	(20.47)	9,517	(20.17)	19,161	(20.32)	
65-79	11,414	(24.22)	11,509	(24.39)	22,923	(24.31)	
80+	2,844	(6.04)	2,835	(6.01)	5,679	(6.02)	
Sex							-0.005
Female	7,456	(15.82)	7,375	(15.63)	14,831	(15.73)	
Male	39,649	(84.14)	39,801	(84.34)	79,450	(84.24)	
Sex Missing	15	(0.03)	14	(0.03)	29	(0.03)	
Race							-0.001
White	27,939	(59.29)	28,010	(59.36)	55,949	(59.32)	
Black	12,271	(26.04)	12,224	(25.90)	24,495	(25.97)	
Asian	542	(1.15)	562	(1.19)	1,104	(1.17)	
More Than One Race	726	(1.54)	708	(1.50)	1,434	(1.52)	
American Indian or Alaska Native	507	(1.08)	527	(1.12)	1,034	(1.10)	
Native Hawaiian or Other Pacific Islander	473	(1.00)	497	(1.05)	970	(1.03)	
Race Unknown	4,662	(9.89)	4,662	(9.88)	9,324	(9.89)	
Ethnicity							-0.002
Hispanic	3,744	(7.95)	3,646	(7.73)	7,390	(7.84)	
Not Hispanic	39,465	(83.75)	39,690	(84.11)	79,155	(83.93)	
Ethnicity Unknown	3,911	(8.30)	3,854	(8.17)	7,765	(8.23)	
Marital status							0.003
Married	16,822	(35.70)	16,957	(35.93)	33,779	(35.82)	
Single/Never Married	11,638	(24.70)	11,586	(24.55)	23,224	(24.63)	
Divorced/Separated/Widowed	17,495	(37.13)	17,461	(37.00)	34,956	(37.06)	
Marital status unknown	1,165	(2.47)	1,186	(2.51)	2,351	(2.49)	
Branch of service							-0.002
Air Force	5,999	(12.73)	5,948	(12.60)	11,947	(12.67)	
Army	24,405	(51.79)	24,497	(51.91)	48,902	(51.85)	
Navy	9,240	(19.61)	9,254	(19.61)	18,494	(19.61)	
Marine Corps	6,131	(13.01)	6,131	(12.99)	12,262	(13.00)	
Other and missing	1,345	(2.85)	1,360	(2.88)	2,705	(2.87)	
Discharge type							0.003
Honorable	41,946	(89.02)	42,051	(89.11)	83,997	(89.06)	
Dishonorable	378	(0.80)	376	(0.80)	754	(0.80)	
Other	3,543	(7.52)	3,534	(7.49)	7,077	(7.50)	
Unknown	1,253	(2.66)	1,229	(2.60)	2,482	(2.63)	
Past SRE	3,006	(6.38)	2,978	(6.31)	5,984	(6.35)	0.003
Past IP MH	3,205	(6.80)	3,222	(6.83)	6,427	(6.81)	-0.001
Past OP MH	25,489	(54.09)	25,576	(54.20)	51,065	(54.15)	-0.002
Acquired immune deficiency syndrome	413	(0.88)	454	(0.96)	867	(0.92)	-0.009
Alcohol disorder	12,067	(25.61)	12,063	(25.56)	24,130	(25.59)	
Anemias due to other nutritional deficiencies	5,106	(10.84)	4,991	(10.58)	10,097	(10.71)	0.008
Autoimmune conditions	1,170	(2.48)	1,147	(2.43)	2,317	(2.46)	0.003
Chronic blood loss (iron deficiency)	394	(0.84)	385	(0.82)	779	(0.83)	0.002
Leukemia	135	(0.29)	136	(0.29)	271	(0.29)	-0.000
Lymphoma	228	(0.48)	203	(0.43)	431	(0.46)	0.008
Metastatic cancer	263	(0.56)	289	(0.61)	552	(0.59)	-0.007
Solid tumor without metastasis, in situ	1,261	(2.68)	1,162	(2.46)	2,423	(2.57)	0.005
Solid tumor without metastasis, malignant	2,460	(5.22)	2,411	(5.11)	4,871	(5.16)	0.005
Cerebrovascular disease - Primary	7,496	(15.91)	7,480	(15.85)	14,976	(15.88)	0.002
Cerebrovascular disease - Sequela	850	(1.80)	853	(1.81)	1,703	(1.81)	-0.000

© 2024 Reger MA et al. *JAMA Network Open.*

Coagulopathy	1,236	(2.62)	1,269	(2.69)	2,505	(2.66)	-0.004
Dementia	1,288	(2.73)	1,324	(2.81)	2,612	(2.77)	-0.004
Depression	24,590	(52.19)	24,680	(52.30)	49,270	(52.24)	-0.002
Diabetes with chronic complications	6,307	(13.38)	6,321	(13.39)	12,628	(13.39)	-0.000
Diabetes without chronic complications	8,344	(17.71)	8,299	(17.59)	16,643	(17.65)	0.003
Drug use disorder	9,916	(21.04)	9,852	(20.88)	19,768	(20.96)	0.004
Fluid and electrolyte disorders	4,810	(10.21)	4,742	(10.05)	9,552	(10.13)	0.005
Heart failure	2,731	(5.80)	2,679	(5.68)	5,410	(5.74)	0.005
Homeless	7,465	(15.84)	7,456	(15.80)	14,921	(15.82)	0.001
Hypertension, complicated	3,674	(7.80)	3,686	(7.81)	7,360	(7.80)	-0.001
Hypertension, uncomplicated	19,320	(41.00)	19,170	(40.62)	38,490	(40.81)	0.008
Liver disease, mild	4,032	(8.56)	4,092	(8.67)	8,124	(8.61)	-0.004
Liver disease and failure, moderate to severe	370	(0.79)	393	(0.83)	763	(0.81)	-0.005
Liver disease	49	(0.10)	57	(0.12)	106	(0.11)	-0.005
Chronic pulmonary disease	7,623	(16.18)	7,697	(16.31)	15,320	(16.24)	-0.004
Neurological disorders affecting movement	1,425	(3.02)	1,446	(3.06)	2,871	(3.04)	-0.002
Other neurological disorders	2,708	(5.75)	2,769	(5.87)	5,477	(5.81)	-0.005
Seizures and epilepsy	1,644	(3.49)	1,618	(3.43)	3,262	(3.46)	0.003
Obesity	9,050	(19.21)	9,225	(19.55)	18,275	(19.38)	-0.009
Paralysis	687	(1.46)	679	(1.44)	1,366	(1.45)	0.002
Peripheral vascular disease	3,298	(7.00)	3,315	(7.02)	6,613	(7.01)	-0.001
Psychoses	12,169	(25.83)	12,305	(26.08)	24,474	(25.95)	-0.006
Pulmonary circulation disease	736	(1.56)	762	(1.61)	1,498	(1.59)	-0.004
Renal (kidney) failure and disease, moderate	2,659	(5.64)	2,661	(5.64)	5,320	(5.64)	0.000
Renal (kidney) failure and disease, severe	589	(1.25)	557	(1.18)	1,146	(1.22)	0.006
Renal failure	687	(1.46)	681	(1.44)	1,368	(1.45)	0.001
Hypothyroidism	3,034	(6.44)	3,095	(6.56)	6,129	(6.50)	-0.005
Other thyroid disorders	903	(1.92)	790	(1.67)	1,693	(1.80)	0.018
Peptic ulcer with bleeding	436	(0.93)	450	(0.95)	886	(0.94)	-0.003
Valvular disease	1,181	(2.51)	1,189	(2.52)	2,370	(2.51)	-0.001
Weight loss	2,004	(4.25)	1,989	(4.21)	3,993	(4.23)	0.002

© 2024 Reger MA et al. *JAMA Network Open.*

eTable 4. Rates of all Outcomes for Peer-Provider Signatory Comparison

Outcome (one year forward)	Total Sample (N=94,310)	Peer Signatory (N=47,120)	Provider Signatory (N=47,190)	P-value+
**Any Nonfatal suicide attempt and intentional self-harm**				
At least one event, n (%)	7,056 (7.48)	3,489 (7.40)	3,567 (7.56)	0.368
Frequency, Mean (std. dev)	0.21 (1.52)	0.20 (1.53)	0.21 (1.52)	0.372
**All-cause mortality**				
All-cause mortality, n (%)	3,528 (3.74)	1,777 (3.77)	1,751 (3.71)	0.623
**Any outpatient utilization**				
At least one visit, n (%)	87,558 (92.84)	43,725 (92.79)	43,833 (92.89)	0.587
Visit count, mean (std. dev)	22.20 (22.84)	22.25 (0.484)	22.15 (22.80)	0.818
**Outpatient mental health utilization**				
At least one visit, n (%)	64,830 (68.74)	32,494 (68.96)	32,336 (68.52)	0.148
Visit count, mean (std. dev)	8.71 (15.26)	8.69 (15.09)	8.74 (15.43)	0.481
**Any inpatient utilization**				
At least one visit, n (%)	19,184 (20.34)	9,555 (20.28)	9,629 (20.40)	0.629
Visit count, mean (std. dev)	0.46 (1.33)	0.46 (1.31)	0.46 (1.35)	0.795
**Inpatient mental health utilization**				
At least one visit, n (%)	9,794 (10.38)	4,855 (10.30)	4,939 (10.47)	0.413
Visit count, mean (std. dev)	0.18 (0.70)	0.17 (0.70)	0.18 (0.70)	0.426
**Emergency department utilization**				
At least one visit, n (%)	35,227 (37.35)	17,653 (37.46)	17,574 (37.24)	0.479
Visit count, mean (std. dev)	1.60 (3.71)	1.62 (3.71)	1.59 (3.72)	0.546

Notes: +Chi-squared test was used for categorical variables, Wilcoxon rank-sum test was used for frequencies.

© 2024 Reger MA et al. *JAMA Network Open.*

eTable5. Average Incremental Effects From Logistic Regressions of Association between Outcomes and Caring Letters Signatory

VARIABLES	All-Cause Mortality	Suicide Attempt	Outpatient Care	Outpatient Mental Health Care	Inpatient Care	Inpatient Mental Health Care	Emergency Department Use
Randomization type (ref. Peer)	-0.001	0.001	0.001	-0.004	0.001	0.001	-0.002
	(-0.003 - 0.002)	(-0.002 - 0.005)	(-0.002 - 0.003)	(-0.009 - 0.001)	(-0.003 - 0.006)	(-0.003 - 0.005)	(-0.008 - 0.004)
	0.518	0.389	0.678	0.105	0.601	0.581	0.501
Age in years	0.002***	-0.002***	-0.000***	-0.005***	-0.000***	-0.001***	-0.002***
	(0.001 - 0.002)	(-0.002 - -0.001)	(-0.001 - -0.000)	(-0.006 - -0.005)	(-0.001 - -0.000)	(-0.001 - -0.001)	(-0.002 - -0.002)
	0.000	0.000	0.000	0.000	0.000	0.000	0.000
Female sex	-0.015***	-0.004*	0.008***	0.034***	-0.030***	-0.018***	-0.007
	(-0.019 - -0.011)	(-0.008 - 0.000)	(0.004 - 0.012)	(0.026 - 0.042)	(-0.037 - -0.024)	(-0.023 - -0.013)	(-0.015 - 0.001)
	0.000	0.078	0.000	0.000	0.000	0.000	0.107
Race (ref. White)							
Black	-0.012***	-0.011***	0.007***	0.013***	-0.002	0.003	0.054***
	(-0.015 - -0.009)	(-0.015 - -0.007)	(0.003 - 0.010)	(0.007 - 0.020)	(-0.008 - 0.004)	(-0.002 - 0.007)	(0.046 - 0.061)
	0.000	0.000	0.000	0.000	0.500	0.292	0.000
Asian	-0.014**	0.000	0.015***	-0.025**	-0.020*	-0.005	-0.005
	(-0.026 - -0.002)	(-0.014 - 0.015)	(0.005 - 0.026)	(-0.049 - -0.000)	(-0.043 - 0.004)	(-0.022 - 0.013)	(-0.032 - 0.022)
	0.020	0.953	0.005	0.049	0.097	0.614	0.712
More Than One Race	-0.016***	-0.010	0.002	-0.000	-0.008	-0.007	0.013
	(-0.026 - -0.007)	(-0.021 - 0.002)	(-0.009 - 0.013)	(-0.022 - 0.022)	(-0.027 - 0.012)	(-0.021 - 0.007)	(-0.010 - 0.037)
	0.001	0.116	0.719	0.978	0.446	0.300	0.271
American Indian or Alaska Native	0.004	0.007	-0.002	-0.010	0.016	-0.009	0.009
	(-0.010 - 0.018)	(-0.008 - 0.022)	(-0.015 - 0.012)	(-0.035 - 0.016)	(-0.007 - 0.039)	(-0.025 - 0.007)	(-0.018 - 0.037)
	0.596	0.339	0.821	0.457	0.173	0.295	0.514
Native Hawaiian or Other Pacific Islander	-0.013**	0.001	0.019***	0.028**	-0.013	-0.004	0.020
	(-0.024 - -0.002)	(-0.015 - 0.017)	(0.007 - 0.031)	(0.003 - 0.053)	(-0.037 - 0.011)	(-0.023 - 0.014)	(-0.009 - 0.050)
	0.018	0.931	0.002	0.028	0.285	0.654	0.182
Race Unknown	-0.001	-0.006*	-0.014***	-0.015***	-0.007	-0.003	0.003
	(-0.007 - 0.004)	(-0.012 - 0.000)	(-0.020 - -0.009)	(-0.026 - -0.005)	(-0.017 - 0.003)	(-0.011 - 0.005)	(-0.009 - 0.014)
	0.647	0.057	0.000	0.004	0.175	0.442	0.665
Ethnicity (ref. Not Hispanic)							
Hispanic	-0.006**	0.004	0.009***	0.012**	-0.008*	-0.008**	0.022***
	(-0.011 - -0.001)	(-0.003 - 0.010)	(0.004 - 0.014)	(0.002 - 0.023)	(-0.018 - 0.001)	(-0.015 - -0.000)	(0.010 - 0.034)
	0.012	0.260	0.001	0.023	0.097	0.036	0.000
Ethnicity Unknown	0.004	0.007*	-0.012***	0.004	0.003	0.005	-0.010
	(-0.003 - 0.010)	(-0.001 - 0.014)	(-0.017 - -0.007)	(-0.007 - 0.015)	(-0.009 - 0.015)	(-0.004 - 0.014)	(-0.023 - 0.003)
	0.252	0.078	0.000	0.438	0.618	0.256	0.142
Marital Status (ref. Married)							
Single/Never Married	0.008***	-0.003	-0.006***	-0.013***	0.030***	0.011***	0.046***
	(0.004 - 0.011)	(-0.007 - 0.001)	(-0.010 - -0.002)	(-0.020 - -0.006)	(0.023 - 0.036)	(0.006 - 0.016)	(0.038 - 0.054)
	0.000	0.192	0.003	0.000	0.000	0.000	0.000
Divorced/Separated/	0.008***	0.002	-0.000	-0.001	0.033***	0.011***	0.048***
Widowed	(0.005 - 0.011)	(-0.002 - 0.006)	(-0.004 - 0.003)	(-0.007 - 0.005)	(0.027 - 0.038)	(0.007 - 0.016)	(0.041 - 0.055)
	0.000	0.336	0.940	0.773	0.000	0.000	0.000
Marital Status Unknown	0.002	-0.004	-0.033***	-0.070***	-0.061***	-0.036***	-0.107***

© 2024 Reger MA et al. *JAMA Network Open.*

	(-0.009 - 0.014)	(-0.016 - 0.008)	(-0.042 - -0.025)	(-0.089 - -0.051)	(-0.081 - -0.042)	(-0.049 - -0.022)	(-0.130 - -0.084)
	0.678	0.512	0.000	0.000	0.000	0.000	0.000
Age at Separation Quartile (ref. quartile 1)							
Quartile 2	0.001	-0.003	0.000	-0.001	0.001	-0.002	-0.008*
	(-0.002 - 0.004)	(-0.008 - 0.002)	(-0.004 - 0.004)	(-0.008 - 0.006)	(-0.005 - 0.008)	(-0.007 - 0.003)	(-0.016 - 0.001)
	0.465	0.218	0.810	0.796	0.734	0.375	0.068
Quartile 3	-0.001	-0.003	0.001	0.009**	0.003	-0.000	-0.002
	(-0.004 - 0.003)	(-0.008 - 0.001)	(-0.003 - 0.005)	(0.001 - 0.016)	(-0.004 - 0.010)	(-0.006 - 0.005)	(-0.010 - 0.006)
	0.746	0.158	0.538	0.024	0.408	0.867	0.612
Quartile 4	-0.001	-0.003	0.009***	0.029***	-0.002	-0.000	-0.013***
	(-0.004 - 0.003)	(-0.008 - 0.002)	(0.005 - 0.013)	(0.021 - 0.036)	(-0.009 - 0.005)	(-0.006 - 0.005)	(-0.021 - -0.004)
	0.681	0.223	0.000	0.000	0.515	0.900	0.003
Military Branch (ref. Army)							
Air Force	-0.000	0.002	0.002	0.009**	-0.006*	-0.000	-0.001
	(-0.004 - 0.003)	(-0.003 - 0.007)	(-0.003 - 0.006)	(0.001 - 0.017)	(-0.014 - 0.001)	(-0.006 - 0.006)	(-0.010 - 0.008)
	0.861	0.433	0.440	0.024	0.095	0.973	0.903
Navy	-0.002	0.003	-0.002	0.011***	0.002	0.001	0.005
	(-0.005 - 0.001)	(-0.001 - 0.007)	(-0.006 - 0.002)	(0.004 - 0.017)	(-0.004 - 0.009)	(-0.004 - 0.005)	(-0.003 - 0.012)
	0.197	0.185	0.297	0.002	0.443	0.804	0.241
Marine Corps	-0.001	0.002	0.002	0.003	0.001	-0.001	-0.006
	(-0.005 - 0.003)	(-0.003 - 0.007)	(-0.002 - 0.006)	(-0.005 - 0.010)	(-0.006 - 0.008)	(-0.006 - 0.005)	(-0.015 - 0.003)
	0.533	0.370	0.320	0.526	0.791	0.793	0.200
Other or missing	-0.004	0.001	-0.003	0.016	0.002	-0.002	-0.002
	(-0.014 - 0.007)	(-0.015 - 0.016)	(-0.016 - 0.010)	(-0.008 - 0.039)	(-0.020 - 0.025)	(-0.019 - 0.016)	(-0.030 - 0.025)
	0.469	0.931	0.635	0.186	0.844	0.859	0.869
Discharge Type (ref. Honorable)							
Dishonorable	0.008	0.006	-0.015***	-0.021	0.010	0.010	0.031
	(-0.010 - 0.026)	(-0.014 - 0.026)	(-0.026 - -0.004)	(-0.050 - 0.007)	(-0.022 - 0.042)	(-0.014 - 0.033)	(-0.006 - 0.069)
	0.377	0.550	0.009	0.144	0.549	0.414	0.105
Other	0.008***	0.007**	-0.008***	-0.005	0.013***	0.010***	0.014**
	(0.002 - 0.015)	(0.001 - 0.012)	(-0.013 - -0.003)	(-0.015 - 0.006)	(0.004 - 0.023)	(0.003 - 0.016)	(0.003 - 0.026)
	0.008	0.025	0.002	0.360	0.005	0.005	0.012
Unknown	0.009	0.009	0.001	-0.002	-0.002	-0.001	0.012
	(-0.005 - 0.023)	(-0.009 - 0.027)	(-0.012 - 0.014)	(-0.028 - 0.024)	(-0.027 - 0.022)	(-0.021 - 0.018)	(-0.019 - 0.042)
	0.207	0.323	0.854	0.893	0.844	0.906	0.455
Any inpatient mental health stay in the past year	0.000	0.024***	-0.023***	0.055***	0.116***	0.110***	0.105***
	(-0.005 - 0.006)	(0.018 - 0.030)	(-0.039 - -0.007)	(0.039 - 0.072)	(0.104 - 0.127)	(0.101 - 0.119)	(0.091 - 0.119)
	0.927	0.000	0.005	0.000	0.000	0.000	0.000
Any outpatient mental health visit in the past year	-0.006***	0.012***	0.048***	0.214***	0.019***	0.024***	0.059***
	(-0.009 - -0.003)	(0.008 - 0.016)	(0.045 - 0.052)	(0.207 - 0.221)	(0.013 - 0.025)	(0.019 - 0.029)	(0.051 - 0.066)
	0.000	0.000	0.000	0.000	0.000	0.000	0.000
Any SRE in the past year	0.001	0.197***	0.041***	0.147***	0.103***	0.076***	0.061***
	(-0.005 - 0.007)	(0.186 - 0.207)	(0.036 - 0.046)	(0.135 - 0.160)	(0.092 - 0.114)	(0.068 - 0.084)	(0.049 - 0.074)
	0.694	0.000	0.000	0.000	0.000	0.000	0.000
Elixhauser comorbidities							
Acquired immune deficiency syndrome	-0.012**	0.010	0.030***	0.006	0.024**	0.010	0.074***
	(-0.021 - -0.002)	(-0.007 - 0.026)	(0.013 - 0.047)	(-0.022 - 0.035)	(0.001 - 0.047)	(-0.007 - 0.027)	(0.043 - 0.105)

© 2024 Reger MA et al. *JAMA Network Open.*

	0.021	0.249	0.000	0.670	0.044	0.231	0.000
Alcohol disorder	0.010***	0.016***	0.008***	0.026***	0.067***	0.028***	0.031***
	(0.006 - 0.013)	(0.011 - 0.020)	(0.003 - 0.013)	(0.018 - 0.033)	(0.060 - 0.074)	(0.024 - 0.033)	(0.024 - 0.039)
	0.000	0.000	0.001	0.000	0.000	0.000	0.000
Anemias due to other nutritional deficiencies	0.003*	0.000	0.018***	-0.008*	0.015***	-0.004	0.020***
	(-0.000 - 0.006)	(-0.006 - 0.006)	(0.010 - 0.025)	(-0.018 - 0.001)	(0.008 - 0.023)	(-0.010 - 0.002)	(0.009 - 0.030)
	0.091	0.955	0.000	0.089	0.000	0.201	0.000
Autoimmune conditions	-0.002	-0.000	0.028***	0.013	0.019**	-0.003	0.058***
	(-0.008 - 0.005)	(-0.011 - 0.010)	(0.015 - 0.040)	(-0.004 - 0.030)	(0.004 - 0.034)	(-0.014 - 0.008)	(0.039 - 0.077)
	0.607	0.961	0.000	0.130	0.011	0.578	0.000
Chronic blood loss (iron deficiency)	0.007	-0.002	-0.006	-0.002	0.020	-0.010	0.015
	(-0.003 - 0.017)	(-0.020 - 0.015)	(-0.042 - 0.030)	(-0.032 - 0.028)	(-0.005 - 0.044)	(-0.028 - 0.008)	(-0.018 - 0.047)
	0.156	0.817	0.745	0.894	0.118	0.290	0.380
Leukemia	0.016*	-0.026*	0.027	-0.005	0.005	-0.041***	0.031
	(-0.002 - 0.034)	(-0.058 - 0.005)	(-0.006 - 0.060)	(-0.050 - 0.040)	(-0.037 - 0.046)	(-0.071 - -0.010)	(-0.023 - 0.085)
	0.079	0.096	0.105	0.829	0.821	0.009	0.265
Lymphoma	0.015**	-0.024**	0.045***	-0.001	0.014	-0.031**	0.010
	(0.001 - 0.029)	(-0.048 - -0.001)	(0.025 - 0.066)	(-0.037 - 0.034)	(-0.019 - 0.046)	(-0.055 - -0.006)	(-0.032 - 0.052)
	0.033	0.041	0.000	0.936	0.414	0.016	0.649
Metastatic cancer	0.074***	-0.018	0.024	-0.074***	0.099***	-0.012	0.095***
	(0.055 - 0.093)	(-0.041 - 0.006)	(-0.011 - 0.060)	(-0.109 - -0.038)	(0.064 - 0.133)	(-0.038 - 0.014)	(0.054 - 0.136)
	0.000	0.139	0.182	0.000	0.000	0.358	0.000
Solid tumor without metastasis, in situ	-0.002	-0.014**	0.022***	-0.004	-0.014*	-0.012*	-0.015
	(-0.008 - 0.003)	(-0.026 - -0.001)	(0.010 - 0.035)	(-0.020 - 0.013)	(-0.029 - 0.001)	(-0.025 - 0.002)	(-0.034 - 0.005)
	0.388	0.028	0.000	0.677	0.061	0.086	0.134
Solid tumor without metastasis, malignant	0.009***	-0.006	0.027***	-0.012*	0.023***	-0.007	0.038***
	(0.005 - 0.014)	(-0.015 - 0.003)	(0.018 - 0.036)	(-0.024 - 0.001)	(0.011 - 0.035)	(-0.017 - 0.002)	(0.023 - 0.053)
	0.000	0.213	0.000	0.063	0.000	0.135	0.000
Cerebrovascular disease - Primary	0.003*	0.002	0.020***	-0.001	0.033***	0.006*	0.046***
	(-0.000 - 0.006)	(-0.003 - 0.007)	(0.014 - 0.026)	(-0.009 - 0.007)	(0.026 - 0.040)	(-0.000 - 0.011)	(0.037 - 0.055)
	0.054	0.518	0.000	0.810	0.000	0.051	0.000
Cerebrovascular disease - Sequela	-0.001	0.002	-0.021	-0.009	-0.004	-0.002	0.014
	(-0.008 - 0.006)	(-0.013 - 0.017)	(-0.048 - 0.007)	(-0.031 - 0.014)	(-0.022 - 0.015)	(-0.017 - 0.014)	(-0.012 - 0.039)
	0.755	0.797	0.140	0.445	0.700	0.829	0.288
Coagulopathy	0.015***	-0.001	0.020**	0.010	0.051***	0.011**	0.049***
	(0.009 - 0.022)	(-0.011 - 0.009)	(0.004 - 0.037)	(-0.007 - 0.028)	(0.035 - 0.066)	(0.000 - 0.023)	(0.029 - 0.070)
	0.000	0.815	0.015	0.252	0.000	0.042	0.000
Dementia	0.012***	-0.001	-0.027***	0.000	0.013*	0.009	0.005
	(0.006 - 0.017)	(-0.012 - 0.010)	(-0.046 - -0.009)	(-0.016 - 0.016)	(-0.001 - 0.027)	(-0.003 - 0.020)	(-0.014 - 0.023)
	0.000	0.795	0.004	0.981	0.071	0.148	0.613
Depression	-0.002	0.016***	0.028***	0.070***	0.011***	0.004*	0.029***
	(-0.005 - 0.000)	(0.012 - 0.020)	(0.024 - 0.031)	(0.064 - 0.076)	(0.005 - 0.017)	(-0.001 - 0.008)	(0.022 - 0.036)
	0.101	0.000	0.000	0.000	0.000	0.098	0.000
Diabetes with chronic complications	0.007***	-0.002	0.004	-0.005	0.023***	-0.002	0.038***
	(0.003 - 0.011)	(-0.010 - 0.005)	(-0.005 - 0.013)	(-0.015 - 0.006)	(0.013 - 0.032)	(-0.010 - 0.005)	(0.026 - 0.050)
	0.001	0.519	0.362	0.382	0.000	0.527	0.000
Diabetes without chronic complications	-0.001	0.001	0.028***	-0.000	0.006	0.005	0.009

© 2024 Reger MA et al. *JAMA Network Open.*

	(-0.004 - 0.003)	(-0.006 - 0.007)	(0.022 - 0.034)	(-0.009 - 0.009)	(-0.003 - 0.014)	(-0.003 - 0.012)	(-0.002 - 0.019)
	0.657	0.830	0.000	0.990	0.183	0.213	0.105
Drug use disorder	0.001	0.009***	0.008***	0.036***	0.048***	0.032***	0.051***
	(-0.003 - 0.004)	(0.004 - 0.013)	(0.002 - 0.014)	(0.027 - 0.044)	(0.041 - 0.055)	(0.027 - 0.038)	(0.042 - 0.059)
	0.755	0.000	0.007	0.000	0.000	0.000	0.000
Fluid and electrolyte disorders	0.009***	0.008***	-0.003	0.004	0.079***	0.018***	0.103***
	(0.005 - 0.013)	(0.003 - 0.014)	(-0.013 - 0.008)	(-0.007 - 0.014)	(0.069 - 0.088)	(0.012 - 0.025)	(0.092 - 0.115)
	0.000	0.005	0.630	0.492	0.000	0.000	0.000
Heart failure	0.021***	-0.006	0.003	-0.024***	0.038***	-0.013***	0.017**
	(0.016 - 0.026)	(-0.015 - 0.002)	(-0.011 - 0.016)	(-0.037 - -0.011)	(0.027 - 0.050)	(-0.021 - -0.004)	(0.003 - 0.032)
	0.000	0.150	0.699	0.000	0.000	0.005	0.021
Homeless	0.003	0.008***	-0.003	0.039***	0.046***	0.031***	0.063***
	(-0.001 - 0.007)	(0.003 - 0.012)	(-0.008 - 0.003)	(0.031 - 0.048)	(0.039 - 0.053)	(0.026 - 0.036)	(0.054 - 0.072)
	0.127	0.001	0.381	0.000	0.000	0.000	0.000
Hypertension, complicated	0.001	0.006	0.002	-0.011*	0.028***	0.002	0.029***
	(-0.003 - 0.005)	(-0.002 - 0.015)	(-0.009 - 0.013)	(-0.022 - 0.001)	(0.018 - 0.039)	(-0.007 - 0.010)	(0.016 - 0.042)
	0.622	0.147	0.712	0.073	0.000	0.671	0.000
Hypertension, uncomplicated	-0.003*	0.004*	0.038***	0.006*	0.017***	0.001	0.030***
	(-0.005 - 0.000)	(-0.000 - 0.008)	(0.035 - 0.042)	(-0.000 - 0.013)	(0.011 - 0.023)	(-0.004 - 0.005)	(0.023 - 0.038)
	0.087	0.054	0.000	0.054	0.000	0.794	0.000
Liver disease, mild	0.008***	-0.001	0.027***	0.013**	0.040***	0.014***	0.048***
	(0.003 - 0.012)	(-0.007 - 0.005)	(0.020 - 0.035)	(0.003 - 0.023)	(0.031 - 0.048)	(0.007 - 0.020)	(0.037 - 0.059)
	0.000	0.702	0.000	0.013	0.000	0.000	0.000
Liver disease and failure, moderate to severe	0.018***	-0.009	-0.003	-0.025	0.019	-0.017**	0.002
	(0.007 - 0.030)	(-0.025 - 0.008)	(-0.051 - 0.045)	(-0.057 - 0.008)	(-0.006 - 0.044)	(-0.033 - -0.001)	(-0.033 - 0.037)
	0.002	0.298	0.911	0.134	0.134	0.039	0.900
Liver disease	0.004	-0.027*		-0.024	0.087**	0.008	0.112*
	(-0.019 - 0.026)	(-0.058 - 0.004)		(-0.113 - 0.064)	(0.005 - 0.169)	(-0.034 - 0.051)	(-0.001 - 0.226)
	0.746	0.091		0.588	0.037	0.710	0.053
Chronic pulmonary disease	0.005***	0.005**	0.028***	0.010***	0.012***	0.005*	0.031***
	(0.002 - 0.008)	(0.000 - 0.010)	(0.023 - 0.033)	(0.003 - 0.018)	(0.005 - 0.018)	(-0.000 - 0.010)	(0.023 - 0.040)
	0.001	0.049	0.000	0.007	0.000	0.076	0.000
Neurological disorders affecting movement	0.001	0.018***	0.012*	0.031***	0.016**	0.021***	0.028***
	(-0.005 - 0.006)	(0.008 - 0.028)	(-0.002 - 0.026)	(0.016 - 0.046)	(0.003 - 0.029)	(0.010 - 0.031)	(0.011 - 0.045)
	0.761	0.000	0.081	0.000	0.014	0.000	0.001
Other neurological disorders	0.000	-0.007**	0.018***	0.022***	0.011**	-0.005	0.025***
	(-0.004 - 0.004)	(-0.014 - -0.000)	(0.008 - 0.028)	(0.010 - 0.034)	(0.001 - 0.021)	(-0.012 - 0.002)	(0.012 - 0.038)
	0.977	0.047	0.001	0.000	0.031	0.185	0.000
Seizures and epilepsy	0.009***	0.012***	0.014**	-0.006	0.020***	0.002	0.023***
	(0.003 - 0.016)	(0.004 - 0.021)	(0.002 - 0.027)	(-0.022 - 0.010)	(0.008 - 0.032)	(-0.006 - 0.011)	(0.007 - 0.039)
	0.004	0.004	0.019	0.473	0.002	0.594	0.006
Obesity	-0.005***	-0.003	0.027***	0.020***	0.006*	0.002	0.032***
	(-0.008 - -0.002)	(-0.007 - 0.002)	(0.022 - 0.032)	(0.013 - 0.027)	(-0.000 - 0.012)	(-0.003 - 0.007)	(0.025 - 0.040)
	0.001	0.233	0.000	0.000	0.062	0.386	0.000
Paralysis	0.006	0.006	0.000	-0.040***	0.024**	-0.008	0.006
	(-0.003 - 0.015)	(-0.011 - 0.022)	(-0.025 - 0.025)	(-0.066 - -0.015)	(0.003 - 0.046)	(-0.024 - 0.008)	(-0.022 - 0.034)
	0.215	0.508	0.998	0.002	0.027	0.341	0.654

© 2024 Reger MA et al. *JAMA Network Open.*

Peripheral vascular disease	0.005***	-0.006	0.028***	-0.005	0.023***	-0.008**	0.039***
	(0.001 - 0.009)	(-0.013 - 0.002)	(0.020 - 0.036)	(-0.015 - 0.006)	(0.013 - 0.032)	(-0.016 - -0.001)	(0.027 - 0.051)
	0.007	0.125	0.000	0.360	0.000	0.035	0.000
Psychoses	-0.000	0.014***	0.019***	0.075***	0.045***	0.051***	0.050***
	(-0.004 - 0.003)	(0.010 - 0.018)	(0.014 - 0.023)	(0.068 - 0.082)	(0.039 - 0.051)	(0.047 - 0.056)	(0.042 - 0.057)
	0.794	0.000	0.000	0.000	0.000	0.000	0.000
Pulmonary circulation disease	0.007*	0.004	0.021*	0.016	0.047***	0.008	0.060***
	(-0.000 - 0.013)	(-0.010 - 0.018)	(-0.001 - 0.043)	(-0.005 - 0.036)	(0.029 - 0.066)	(-0.006 - 0.023)	(0.035 - 0.085)
	0.061	0.573	0.056	0.132	0.000	0.267	0.000
Renal (kidney) failure and disease, moderate	0.004*	-0.002	0.011**	-0.006	-0.001	-0.005	0.001
	(-0.000 - 0.009)	(-0.011 - 0.007)	(0.000 - 0.023)	(-0.019 - 0.006)	(-0.011 - 0.010)	(-0.015 - 0.004)	(-0.014 - 0.015)
	0.056	0.691	0.047	0.316	0.919	0.270	0.942
Renal (kidney) failure and disease, severe	0.039***	-0.021**	-0.036**	-0.029**	0.027**	-0.037***	-0.005
	(0.028 - 0.050)	(-0.037 - -0.004)	(-0.067 - -0.005)	(-0.052 - -0.005)	(0.006 - 0.049)	(-0.053 - -0.021)	(-0.032 - 0.023)
	0.000	0.012	0.023	0.017	0.012	0.000	0.741
Renal failure	-0.009***	-0.009	0.033***	-0.001	-0.008	-0.002	0.011
	(-0.015 - -0.004)	(-0.023 - 0.006)	(0.014 - 0.053)	(-0.022 - 0.021)	(-0.026 - 0.009)	(-0.018 - 0.013)	(-0.014 - 0.036)
	0.001	0.242	0.001	0.938	0.362	0.779	0.384
Hypothyroidism	0.001	0.008**	0.031***	0.011**	0.010**	0.011***	0.009
	(-0.003 - 0.005)	(0.001 - 0.015)	(0.024 - 0.038)	(0.000 - 0.022)	(0.001 - 0.020)	(0.003 - 0.019)	(-0.003 - 0.020)
	0.769	0.028	0.000	0.042	0.031	0.006	0.154
Other thyroid disorders	-0.006*	-0.006	0.032***	0.008	-0.010	-0.019***	0.036***
	(-0.013 - 0.001)	(-0.018 - 0.006)	(0.016 - 0.047)	(-0.012 - 0.027)	(-0.026 - 0.006)	(-0.031 - -0.007)	(0.014 - 0.057)
	0.085	0.332	0.000	0.446	0.203	0.002	0.001
Peptic ulcer with bleeding	0.004	0.013	0.002	0.002	0.041***	0.015*	0.039**
	(-0.005 - 0.014)	(-0.004 - 0.029)	(-0.027 - 0.031)	(-0.026 - 0.030)	(0.018 - 0.065)	(-0.002 - 0.033)	(0.007 - 0.070)
	0.352	0.145	0.896	0.910	0.001	0.091	0.015
Valvular disease	0.001	0.001	0.020**	-0.012	0.027***	0.002	0.036***
	(-0.004 - 0.006)	(-0.011 - 0.013)	(0.005 - 0.036)	(-0.028 - 0.005)	(0.012 - 0.042)	(-0.011 - 0.014)	(0.017 - 0.056)
	0.644	0.835	0.011	0.171	0.000	0.799	0.000
Weight loss	0.011***	0.014***	0.020***	-0.001	0.023***	0.002	0.023***
	(0.006 - 0.016)	(0.006 - 0.023)	(0.008 - 0.031)	(-0.015 - 0.012)	(0.012 - 0.034)	(-0.007 - 0.010)	(0.008 - 0.038)
	0.000	0.001	0.001	0.872	0.000	0.701	0.003
Observations	92,625	92,664	92,434	92,664	92,664	92,664	92,664

Notes: DyDx incremental effects with 95% CI in parentheses, followed by p-value; *** p<0.01, ** p<0.05, * p<0.1. Incremental effects calculated after logistic regression of outcomes on the covariates listed above and station fixed effects. We dropped age at separation missing (N=1,646) and recoded branch missing to other due to small cell size. Model observation numbers vary because some variable categories were dropped because there were no outcomes by group. Since logistic regression is fit by maximum likelihood, the maximum likelihood estimate is infinite, and it does not converge. Stata's solution is to omit that variable. All-cause mortality: one station (checklist 59) omitted (39 obs.). Any outpatient use: liver_unk omitted (106 obs.), checklist 59 omitted (39 obs.), checklist 139 omitted (85 obs.).

© 2024 Reger MA et al. *JAMA Network Open.*

eTable 6. Average Incremental Effects from Logistic Regressions of Association between Outcomes and Caring Letters Signatory in Sensitivity Analysis (Excluding deaths 15-30 days after scheduled mail date and opt outs)

VARIABLES	All-Cause Mortality	Suicide Attempt	Outpatient Care	Outpatient Mental Health Care	Inpatient Care	Inpatient Mental Health Care	Emergency Department Use
Randomization type (ref. Peer)	-0.000<br>(-0.003 - 0.002)<br>0.684	0.001<br>(-0.002 - 0.005)<br>0.372	0.001<br>(-0.002 - 0.003)<br>0.692	-0.004<br>(-0.009 - 0.001)<br>0.112	0.001<br>(-0.003 - 0.006)<br>0.604	0.001<br>(-0.003 - 0.005)<br>0.622	-0.002<br>(-0.008 - 0.004)<br>0.464
Age in years	0.002***<br>(0.001 - 0.002)<br>0.000	-0.002***<br>(-0.002 - -0.001)<br>0.000	-0.000***<br>(-0.001 - -0.000)<br>0.000	-0.005***<br>(-0.006 - -0.005)<br>0.000	-0.000***<br>(-0.001 - -0.000)<br>0.000	-0.001***<br>(-0.001 - -0.001)<br>0.000	-0.002***<br>(-0.002 - -0.002)<br>0.000
Female sex	-0.014***<br>(-0.018 - -0.011)<br>0.000	-0.004*<br>(-0.008 - 0.001)<br>0.084	0.008***<br>(0.004 - 0.012)<br>0.000	0.034***<br>(0.026 - 0.042)<br>0.000	-0.031***<br>(-0.037 - -0.024)<br>0.000	-0.018***<br>(-0.023 - -0.013)<br>0.000	-0.007*<br>(-0.015 - 0.001)<br>0.093
Race (ref. White)							
Black	-0.011***<br>(-0.014 - -0.008)<br>0.000	-0.011***<br>(-0.015 - -0.007)<br>0.000	0.007***<br>(0.003 - 0.010)<br>0.000	0.013***<br>(0.006 - 0.020)<br>0.000	-0.002<br>(-0.008 - 0.004)<br>0.472	0.002<br>(-0.002 - 0.007)<br>0.303	0.054***<br>(0.046 - 0.061)<br>0.000
Asian	-0.015**<br>(-0.026 - -0.003)<br>0.011	0.000<br>(-0.015 - 0.015)<br>0.975	0.015***<br>(0.004 - 0.025)<br>0.006	-0.024*<br>(-0.049 - 0.000)<br>0.052	-0.020*<br>(-0.043 - 0.004)<br>0.097	-0.005<br>(-0.023 - 0.013)<br>0.590	-0.005<br>(-0.032 - 0.022)<br>0.716
More Than One Race	-0.017***<br>(-0.026 - -0.008)<br>0.000	-0.009<br>(-0.021 - 0.003)<br>0.131	0.002<br>(-0.009 - 0.013)<br>0.732	-0.000<br>(-0.022 - 0.021)<br>0.975	-0.008<br>(-0.028 - 0.011)<br>0.395	-0.007<br>(-0.021 - 0.007)<br>0.326	0.012<br>(-0.011 - 0.036)<br>0.304
American Indian or Alaska Native	0.006<br>(-0.008 - 0.019)<br>0.423	0.007<br>(-0.008 - 0.022)<br>0.338	-0.002<br>(-0.015 - 0.012)<br>0.809	-0.009<br>(-0.035 - 0.016)<br>0.470	0.016<br>(-0.007 - 0.040)<br>0.170	-0.009<br>(-0.025 - 0.007)<br>0.291	0.008<br>(-0.019 - 0.036)<br>0.557
Native Hawaiian or Other Pacific Islander	-0.012**<br>(-0.022 - -0.001)<br>0.031	0.001<br>(-0.015 - 0.017)<br>0.917	0.018***<br>(0.006 - 0.030)<br>0.003	0.028**<br>(0.003 - 0.053)<br>0.026	-0.014<br>(-0.038 - 0.010)<br>0.257	-0.004<br>(-0.023 - 0.014)<br>0.650	0.019<br>(-0.010 - 0.049)<br>0.197
Race Unknown	-0.001<br>(-0.006 - 0.005)<br>0.779	-0.006*<br>(-0.012 - 0.000)<br>0.061	-0.014***<br>(-0.019 - -0.009)<br>0.000	-0.016***<br>(-0.026 - -0.005)<br>0.003	-0.007<br>(-0.017 - 0.003)<br>0.160	-0.003<br>(-0.011 - 0.005)<br>0.441	0.002<br>(-0.010 - 0.014)<br>0.724
Ethnicity (ref. Not Hispanic)							
Hispanic	-0.006**<br>(-0.011 - -0.001)<br>0.015	0.004<br>(-0.003 - 0.010)<br>0.244	0.009***<br>(0.004 - 0.014)<br>0.001	0.012**<br>(0.001 - 0.023)<br>0.026	-0.008<br>(-0.018 - 0.002)<br>0.103	-0.008**<br>(-0.015 - -0.000)<br>0.037	0.022***<br>(0.010 - 0.034)<br>0.000
Ethnicity Unknown	0.004<br>(-0.003 - 0.010)<br>0.261	0.007*<br>(-0.001 - 0.014)<br>0.084	-0.012***<br>(-0.017 - -0.007)<br>0.000	0.004<br>(-0.006 - 0.015)<br>0.428	0.003<br>(-0.008 - 0.015)<br>0.584	0.005<br>(-0.004 - 0.014)<br>0.249	-0.010<br>(-0.023 - 0.004)<br>0.160

© 2024 Reger MA et al. *JAMA Network Open.*

Marital Status (ref. Married)							
Single/Never Married	0.008***	-0.003	-0.006***	-0.013***	0.030***	0.011***	0.046***
	(0.004 - 0.011)	(-0.007 - 0.002)	(-0.009 - -0.002)	(-0.020 - -0.006)	(0.023 - 0.036)	(0.006 - 0.016)	(0.038 - 0.054)
	0.000	0.207	0.004	0.001	0.000	0.000	0.000
Divorced/Separated/ Widowed	0.007***	0.002	0.000	-0.001	0.033***	0.011***	0.049***
	(0.005 - 0.010)	(-0.002 - 0.006)	(-0.003 - 0.004)	(-0.007 - 0.005)	(0.027 - 0.038)	(0.007 - 0.016)	(0.042 - 0.055)
	0.000	0.285	0.932	0.826	0.000	0.000	0.000
Marital Status Unknown	-0.000	-0.004	-0.033***	-0.070***	-0.063***	-0.036***	-0.108***
	(-0.011 - 0.011)	(-0.017 - 0.008)	(-0.042 - -0.024)	(-0.089 - -0.051)	(-0.082 - -0.044)	(-0.050 - -0.022)	(-0.131 - -0.085)
	0.996	0.476	0.000	0.000	0.000	0.000	0.000
Age at Separation Quartile (ref. quartile 1)							
Quartile 2	0.001	-0.003	0.001	-0.001	0.001	-0.002	-0.007*
	(-0.002 - 0.004)	(-0.008 - 0.002)	(-0.003 - 0.005)	(-0.008 - 0.006)	(-0.005 - 0.008)	(-0.008 - 0.003)	(-0.016 - 0.001)
	0.444	0.231	0.757	0.765	0.701	0.346	0.072
Quartile 3	-0.001	-0.003	0.001	0.008**	0.003	-0.000	-0.002
	(-0.004 - 0.003)	(-0.008 - 0.001)	(-0.003 - 0.006)	(0.001 - 0.016)	(-0.004 - 0.010)	(-0.006 - 0.005)	(-0.010 - 0.006)
	0.685	0.149	0.473	0.027	0.398	0.885	0.648
Quartile 4	-0.001	-0.003	0.009***	0.029***	-0.002	-0.000	-0.013***
	(-0.004 - 0.003)	(-0.008 - 0.002)	(0.005 - 0.013)	(0.021 - 0.036)	(-0.009 - 0.005)	(-0.006 - 0.005)	(-0.021 - -0.004)
	0.673	0.226	0.000	0.000	0.565	0.918	0.004
Military Branch (ref. Army)							
Air Force	-0.001	0.002	0.001	0.009**	-0.006*	-0.000	-0.001
	(-0.004 - 0.003)	(-0.003 - 0.007)	(-0.003 - 0.006)	(0.002 - 0.017)	(-0.014 - 0.001)	(-0.006 - 0.006)	(-0.010 - 0.008)
	0.595	0.512	0.540	0.019	0.096	0.998	0.864
Navy	-0.002	0.003	-0.002	0.011***	0.002	0.001	0.005
	(-0.005 - 0.001)	(-0.002 - 0.007)	(-0.006 - 0.002)	(0.004 - 0.017)	(-0.004 - 0.009)	(-0.004 - 0.006)	(-0.003 - 0.012)
	0.120	0.207	0.272	0.002	0.458	0.765	0.212
Marine Corps	-0.001	0.002	0.002	0.003	0.001	-0.001	-0.006
	(-0.005 - 0.002)	(-0.003 - 0.007)	(-0.002 - 0.006)	(-0.005 - 0.011)	(-0.006 - 0.008)	(-0.006 - 0.005)	(-0.015 - 0.003)
	0.487	0.430	0.336	0.480	0.819	0.798	0.214
Other or missing	-0.003	0.001	-0.003	0.016	0.002	-0.002	-0.002
	(-0.013 - 0.007)	(-0.015 - 0.016)	(-0.015 - 0.010)	(-0.008 - 0.039)	(-0.020 - 0.025)	(-0.019 - 0.016)	(-0.030 - 0.025)
	0.584	0.935	0.694	0.186	0.846	0.856	0.864
Discharge Type (ref. Honorable)							
Dishonorable	0.009	0.006	-0.014**	-0.021	0.010	0.010	0.031
	(-0.008 - 0.027)	(-0.014 - 0.026)	(-0.025 - -0.003)	(-0.049 - 0.008)	(-0.022 - 0.041)	(-0.014 - 0.033)	(-0.007 - 0.069)
	0.296	0.554	0.010	0.153	0.558	0.415	0.110
Other	0.008**	0.006**	-0.008***	-0.005	0.013***	0.010***	0.014**
	(0.002 - 0.014)	(0.001 - 0.012)	(-0.013 - -0.003)	(-0.015 - 0.006)	(0.004 - 0.023)	(0.003 - 0.016)	(0.003 - 0.025)

© 2024 Reger MA et al. *JAMA Network Open.*

	0.010	0.030	0.003	0.392	0.004	0.004	0.013
Unknown	0.008	0.008	0.001	-0.003	-0.004	-0.001	0.011
	(-0.005 - 0.022)	(-0.010 - 0.026)	(-0.012 - 0.014)	(-0.029 - 0.023)	(-0.029 - 0.021)	(-0.021 - 0.018)	(-0.020 - 0.041)
	0.232	0.387	0.887	0.833	0.775	0.900	0.494
Any inpatient mental health stay in the past year	0.001	0.024***	-0.023***	0.055***	0.115***	0.110***	0.104***
	(-0.004 - 0.007)	(0.018 - 0.031)	(-0.040 - -0.007)	(0.038 - 0.071)	(0.104 - 0.127)	(0.101 - 0.119)	(0.090 - 0.118)
	0.587	0.000	0.005	0.000	0.000	0.000	0.000
Any outpatient mental health visit in the past year	-0.006***	0.012***	0.048***	0.214***	0.019***	0.024***	0.059***
	(-0.009 - -0.003)	(0.008 - 0.016)	(0.045 - 0.052)	(0.207 - 0.221)	(0.013 - 0.025)	(0.019 - 0.029)	(0.052 - 0.066)
	0.000	0.000	0.000	0.000	0.000	0.000	0.000
Any SRE in the past year	0.001	0.197***	0.041***	0.147***	0.103***	0.076***	0.061***
	(-0.005 - 0.007)	(0.186 - 0.207)	(0.036 - 0.046)	(0.135 - 0.160)	(0.092 - 0.114)	(0.068 - 0.084)	(0.048 - 0.074)
	0.773	0.000	0.000	0.000	0.000	0.000	0.000
Elixhauser comorbidities							
Acquired immune deficiency syndrome	-0.011**	0.010	0.030***	0.005	0.023*	0.010	0.074***
	(-0.020 - -0.001)	(-0.007 - 0.026)	(0.013 - 0.046)	(-0.024 - 0.034)	(-0.000 - 0.046)	(-0.007 - 0.027)	(0.043 - 0.105)
	0.030	0.244	0.001	0.724	0.051	0.230	0.000
Alcohol disorder	0.009***	0.016***	0.008***	0.026***	0.067***	0.028***	0.031***
	(0.005 - 0.013)	(0.011 - 0.020)	(0.003 - 0.013)	(0.018 - 0.033)	(0.061 - 0.074)	(0.024 - 0.033)	(0.024 - 0.039)
	0.000	0.000	0.001	0.000	0.000	0.000	0.000
Anemias due to other nutritional deficiencies	0.003*	-0.000	0.018***	-0.008*	0.016***	-0.004	0.020***
	(-0.000 - 0.007)	(-0.006 - 0.006)	(0.011 - 0.026)	(-0.018 - 0.001)	(0.008 - 0.024)	(-0.010 - 0.002)	(0.009 - 0.030)
	0.062	0.979	0.000	0.093	0.000	0.213	0.000
Autoimmune conditions	-0.003	0.000	0.027***	0.014*	0.020***	-0.003	0.059***
	(-0.009 - 0.004)	(-0.010 - 0.010)	(0.015 - 0.040)	(-0.003 - 0.031)	(0.005 - 0.035)	(-0.014 - 0.008)	(0.040 - 0.078)
	0.425	0.999	0.000	0.099	0.009	0.596	0.000
Chronic blood loss (iron deficiency)	0.007	-0.003	-0.007	-0.001	0.020	-0.010	0.015
	(-0.002 - 0.017)	(-0.021 - 0.014)	(-0.043 - 0.029)	(-0.030 - 0.029)	(-0.005 - 0.045)	(-0.028 - 0.009)	(-0.017 - 0.048)
	0.142	0.732	0.716	0.971	0.112	0.300	0.357
Leukemia	0.014	-0.026	0.026	-0.004	0.007	-0.040**	0.035
	(-0.003 - 0.032)	(-0.057 - 0.006)	(-0.007 - 0.060)	(-0.048 - 0.041)	(-0.035 - 0.049)	(-0.070 - -0.009)	(-0.020 - 0.089)
	0.104	0.113	0.117	0.875	0.742	0.012	0.212
Lymphoma	0.015**	-0.024**	0.045***	-0.001	0.015	-0.030**	0.012
	(0.001 - 0.029)	(-0.048 - -0.001)	(0.024 - 0.066)	(-0.037 - 0.034)	(-0.018 - 0.048)	(-0.055 - -0.005)	(-0.030 - 0.055)
	0.031	0.043	0.000	0.940	0.367	0.017	0.573
Metastatic cancer	0.069***	-0.017	0.023	-0.070***	0.098***	-0.012	0.097***
	(0.050 - 0.088)	(-0.041 - 0.007)	(-0.014 - 0.060)	(-0.105 - -0.034)	(0.063 - 0.133)	(-0.038 - 0.014)	(0.055 - 0.138)
	0.000	0.154	0.220	0.000	0.000	0.381	0.000
Solid tumor without metastasis, in situ	-0.002	-0.014**	0.021***	-0.003	-0.015**	-0.012*	-0.015

© 2024 Reger MA et al. *JAMA Network Open.*

	(-0.008 - 0.003)	(-0.026 - -0.001)	(0.009 - 0.034)	(-0.020 - 0.013)	(-0.030 - -0.001)	(-0.025 - 0.002)	(-0.035 - 0.004)
	0.373	0.031	0.001	0.705	0.042	0.087	0.117
Solid tumor without metastasis, malignant	0.009***	-0.006	0.028***	-0.012*	0.023***	-0.007	0.038***
	(0.004 - 0.014)	(-0.015 - 0.003)	(0.019 - 0.037)	(-0.024 - 0.001)	(0.011 - 0.035)	(-0.017 - 0.002)	(0.023 - 0.053)
	0.000	0.207	0.000	0.063	0.000	0.131	0.000
Cerebrovascular disease - Primary	0.003*	0.002	0.020***	-0.001	0.033***	0.005*	0.047***
	(-0.000 - 0.006)	(-0.003 - 0.007)	(0.014 - 0.026)	(-0.009 - 0.007)	(0.026 - 0.040)	(-0.000 - 0.011)	(0.037 - 0.056)
	0.068	0.502	0.000	0.803	0.000	0.056	0.000
Cerebrovascular disease - Sequela	-0.001	0.001	-0.022	-0.008	-0.003	-0.001	0.013
	(-0.008 - 0.006)	(-0.014 - 0.016)	(-0.050 - 0.005)	(-0.031 - 0.014)	(-0.022 - 0.015)	(-0.017 - 0.014)	(-0.013 - 0.038)
	0.782	0.863	0.115	0.481	0.713	0.880	0.328
Coagulopathy	0.015***	-0.001	0.022***	0.012	0.051***	0.012**	0.049***
	(0.009 - 0.022)	(-0.011 - 0.009)	(0.006 - 0.038)	(-0.006 - 0.029)	(0.035 - 0.066)	(0.001 - 0.023)	(0.029 - 0.069)
	0.000	0.859	0.008	0.197	0.000	0.036	0.000
Dementia	0.010***	-0.002	-0.026***	0.000	0.013*	0.009	0.006
	(0.004 - 0.015)	(-0.013 - 0.009)	(-0.044 - -0.007)	(-0.016 - 0.016)	(-0.001 - 0.027)	(-0.003 - 0.021)	(-0.013 - 0.024)
	0.001	0.683	0.007	0.961	0.070	0.141	0.557
Depression	-0.002	0.016***	0.028***	0.070***	0.011***	0.004	0.029***
	(-0.005 - 0.001)	(0.012 - 0.019)	(0.024 - 0.031)	(0.064 - 0.077)	(0.005 - 0.016)	(-0.001 - 0.008)	(0.022 - 0.036)
	0.152	0.000	0.000	0.000	0.000	0.104	0.000
Diabetes with chronic complications	0.007***	-0.002	0.005	-0.005	0.022***	-0.002	0.038***
	(0.003 - 0.012)	(-0.010 - 0.005)	(-0.005 - 0.014)	(-0.015 - 0.006)	(0.013 - 0.032)	(-0.010 - 0.005)	(0.026 - 0.050)
	0.001	0.542	0.331	0.374	0.000	0.527	0.000
Diabetes without chronic complications	-0.001	0.001	0.028***	-0.000	0.006	0.005	0.009
	(-0.004 - 0.003)	(-0.006 - 0.007)	(0.022 - 0.034)	(-0.009 - 0.009)	(-0.003 - 0.014)	(-0.002 - 0.012)	(-0.002 - 0.019)
	0.707	0.812	0.000	0.969	0.167	0.190	0.105
Drug use disorder	0.000	0.009***	0.008***	0.036***	0.048***	0.032***	0.051***
	(-0.003 - 0.004)	(0.004 - 0.013)	(0.002 - 0.014)	(0.028 - 0.045)	(0.041 - 0.055)	(0.027 - 0.038)	(0.042 - 0.059)
	0.944	0.000	0.007	0.000	0.000	0.000	0.000
Fluid and electrolyte disorders	0.008***	0.008***	-0.003	0.005	0.079***	0.018***	0.104***
	(0.005 - 0.012)	(0.002 - 0.014)	(-0.013 - 0.008)	(-0.006 - 0.015)	(0.070 - 0.088)	(0.012 - 0.025)	(0.092 - 0.115)
	0.000	0.005	0.643	0.394	0.000	0.000	0.000
Heart failure	0.019***	-0.006	0.006	-0.023***	0.038***	-0.013***	0.019**
	(0.014 - 0.024)	(-0.015 - 0.002)	(-0.007 - 0.019)	(-0.036 - -0.010)	(0.027 - 0.050)	(-0.021 - -0.004)	(0.004 - 0.033)
	0.000	0.156	0.357	0.000	0.000	0.004	0.011
Homeless	0.003	0.008***	-0.003	0.039***	0.047***	0.031***	0.063***
	(-0.001 - 0.006)	(0.003 - 0.012)	(-0.008 - 0.003)	(0.030 - 0.048)	(0.039 - 0.054)	(0.026 - 0.036)	(0.054 - 0.072)

© 2024 Reger MA et al. *JAMA Network Open.*

	0.153	0.001	0.391	0.000	0.000	0.000	0.000
Hypertension, complicated	0.002	0.006	0.002	-0.011*	0.028***	0.002	0.029***
	(-0.002 - 0.006)	(-0.002 - 0.015)	(-0.009 - 0.013)	(-0.023 - 0.000)	(0.018 - 0.039)	(-0.007 - 0.010)	(0.016 - 0.043)
	0.350	0.135	0.737	0.055	0.000	0.698	0.000
Hypertension, uncomplicated	-0.002	0.004*	0.038***	0.006*	0.017***	0.000	0.030***
	(-0.005 - 0.001)	(-0.000 - 0.008)	(0.035 - 0.042)	(-0.000 - 0.013)	(0.011 - 0.023)	(-0.004 - 0.005)	(0.023 - 0.038)
	0.118	0.059	0.000	0.057	0.000	0.844	0.000
Liver disease, mild	0.007***	-0.001	0.027***	0.013**	0.040***	0.014***	0.048***
	(0.003 - 0.012)	(-0.007 - 0.005)	(0.019 - 0.035)	(0.002 - 0.023)	(0.031 - 0.049)	(0.007 - 0.020)	(0.036 - 0.059)
	0.001	0.719	0.000	0.016	0.000	0.000	0.000
Liver disease and failure, moderate to severe	0.016***	-0.008	-0.004	-0.025	0.019	-0.016**	0.003
	(0.005 - 0.027)	(-0.024 - 0.008)	(-0.053 - 0.045)	(-0.057 - 0.007)	(-0.006 - 0.044)	(-0.032 - -0.000)	(-0.032 - 0.038)
	0.004	0.323	0.877	0.130	0.141	0.045	0.874
Liver disease	0.003	-0.027*		-0.032	0.085**	0.008	0.109*
	(-0.019 - 0.025)	(-0.058 - 0.004)		(-0.122 - 0.057)	(0.003 - 0.166)	(-0.034 - 0.051)	(-0.004 - 0.223)
	0.786	0.092		0.479	0.041	0.710	0.059
Chronic pulmonary disease	0.005***	0.005**	0.028***	0.010***	0.012***	0.005*	0.031***
	(0.002 - 0.008)	(0.000 - 0.010)	(0.023 - 0.033)	(0.003 - 0.018)	(0.006 - 0.019)	(-0.000 - 0.010)	(0.023 - 0.039)
	0.001	0.044	0.000	0.007	0.000	0.069	0.000
Neurological disorders affecting movement	0.001	0.019***	0.011	0.031***	0.017**	0.021***	0.028***
	(-0.004 - 0.007)	(0.009 - 0.029)	(-0.002 - 0.025)	(0.016 - 0.046)	(0.004 - 0.030)	(0.011 - 0.032)	(0.011 - 0.045)
	0.710	0.000	0.100	0.000	0.011	0.000	0.001
Other neurological disorders	0.000	-0.007**	0.017***	0.022***	0.011**	-0.005	0.025***
	(-0.004 - 0.005)	(-0.014 - -0.000)	(0.007 - 0.028)	(0.010 - 0.034)	(0.001 - 0.021)	(-0.012 - 0.002)	(0.012 - 0.039)
	0.818	0.046	0.001	0.000	0.025	0.183	0.000
Seizures and epilepsy	0.010***	0.013***	0.014**	-0.007	0.019***	0.002	0.023***
	(0.003 - 0.016)	(0.004 - 0.021)	(0.002 - 0.026)	(-0.023 - 0.009)	(0.007 - 0.031)	(-0.006 - 0.011)	(0.007 - 0.039)
	0.003	0.003	0.024	0.407	0.002	0.610	0.006
Obesity	-0.005***	-0.003	0.027***	0.020***	0.006*	0.002	0.033***
	(-0.008 - -0.002)	(-0.007 - 0.002)	(0.022 - 0.032)	(0.013 - 0.027)	(-0.000 - 0.012)	(-0.003 - 0.007)	(0.025 - 0.040)
	0.001	0.235	0.000	0.000	0.060	0.370	0.000
Paralysis	0.005	0.006	-0.000	-0.041***	0.023**	-0.008	0.005
	(-0.004 - 0.014)	(-0.010 - 0.023)	(-0.025 - 0.024)	(-0.066 - -0.015)	(0.002 - 0.045)	(-0.024 - 0.008)	(-0.023 - 0.033)
	0.265	0.452	0.981	0.002	0.035	0.304	0.716
Peripheral vascular disease	0.005**	-0.006	0.028***	-0.004	0.023***	-0.008**	0.039***
	(0.001 - 0.008)	(-0.013 - 0.002)	(0.020 - 0.036)	(-0.014 - 0.007)	(0.013 - 0.032)	(-0.015 - -0.000)	(0.027 - 0.051)
	0.011	0.129	0.000	0.472	0.000	0.041	0.000
Psychoses	-0.000	0.014***	0.019***	0.075***	0.045***	0.052***	0.050***
	(-0.003 - 0.003)	(0.010 - 0.018)	(0.014 - 0.023)	(0.068 - 0.082)	(0.039 - 0.051)	(0.047 - 0.056)	(0.043 - 0.057)
	0.826	0.000	0.000	0.000	0.000	0.000	0.000

© 2024 Reger MA et al. *JAMA Network Open.*

Pulmonary circulation disease	0.006*	0.004	0.020*	0.016	0.048***	0.007	0.062***
	(-0.000 - 0.013)	(-0.010 - 0.018)	(-0.002 - 0.042)	(-0.005 - 0.036)	(0.029 - 0.067)	(-0.007 - 0.022)	(0.036 - 0.087)
	0.069	0.553	0.074	0.135	0.000	0.330	0.000
Renal (kidney) failure and disease, moderate	0.004*	-0.002	0.012**	-0.006	-0.000	-0.005	0.001
	(-0.001 - 0.008)	(-0.011 - 0.007)	(0.001 - 0.024)	(-0.018 - 0.006)	(-0.011 - 0.011)	(-0.015 - 0.004)	(-0.014 - 0.015)
	0.096	0.658	0.038	0.351	0.968	0.278	0.944
Renal (kidney) failure and disease, severe	0.036***	-0.020**	-0.029*	-0.029**	0.026**	-0.038***	-0.006
	(0.025 - 0.046)	(-0.037 - -0.004)	(-0.060 - 0.002)	(-0.053 - -0.006)	(0.004 - 0.047)	(-0.054 - -0.023)	(-0.034 - 0.022)
	0.000	0.016	0.070	0.015	0.019	0.000	0.673
Renal failure	-0.009***	-0.008	0.033***	-0.002	-0.007	-0.002	0.011
	(-0.014 - -0.003)	(-0.023 - 0.006)	(0.013 - 0.053)	(-0.023 - 0.020)	(-0.025 - 0.010)	(-0.017 - 0.014)	(-0.014 - 0.036)
	0.003	0.250	0.001	0.864	0.412	0.817	0.392
Hypothyroidism	0.000	0.008**	0.031***	0.011**	0.011**	0.011***	0.009
	(-0.004 - 0.004)	(0.001 - 0.015)	(0.023 - 0.038)	(0.000 - 0.022)	(0.001 - 0.020)	(0.003 - 0.019)	(-0.003 - 0.021)
	0.960	0.023	0.000	0.041	0.025	0.005	0.132
Other thyroid disorders	-0.006*	-0.006	0.031***	0.008	-0.011	-0.019***	0.035***
	(-0.013 - 0.001)	(-0.018 - 0.005)	(0.016 - 0.047)	(-0.012 - 0.027)	(-0.027 - 0.005)	(-0.031 - -0.007)	(0.014 - 0.057)
	0.080	0.289	0.000	0.443	0.190	0.002	0.001
Peptic ulcer with bleeding	0.003	0.013	0.002	0.003	0.039***	0.015*	0.038**
	(-0.006 - 0.012)	(-0.004 - 0.030)	(-0.028 - 0.031)	(-0.025 - 0.031)	(0.015 - 0.062)	(-0.002 - 0.033)	(0.007 - 0.070)
	0.526	0.132	0.920	0.817	0.001	0.086	0.016
Valvular disease	0.001	0.001	0.021***	-0.011	0.027***	0.001	0.036***
	(-0.004 - 0.006)	(-0.011 - 0.013)	(0.005 - 0.037)	(-0.028 - 0.006)	(0.012 - 0.042)	(-0.011 - 0.014)	(0.016 - 0.055)
	0.665	0.886	0.010	0.194	0.000	0.835	0.000
Weight loss	0.011***	0.014***	0.020***	-0.002	0.022***	0.002	0.021***
	(0.007 - 0.016)	(0.005 - 0.022)	(0.008 - 0.031)	(-0.015 - 0.012)	(0.010 - 0.033)	(-0.007 - 0.010)	(0.006 - 0.036)
	0.000	0.002	0.001	0.825	0.000	0.705	0.005
Observations	92,371	92,410	92,181	92,410	92,410	92,410	92,410

Notes: DyDx incremental effects with 95% CI in parentheses, followed by p-value; *** p<0.01, ** p<0.05, * p<0.1. Incremental effects calculated after logistic regression of outcomes on the covariates listed above and station fixed effects. We dropped age at separation missing (N=1,641) and recoded branch missing to other due to small cell size. Model observation numbers vary because some variable categories were dropped because there were no outcomes by group.

© 2024 Reger MA et al. *JAMA Network Open.*

eTable 7. Descriptive Statistics by Caring letters Receipt

	Caring Letters Received						
	No Caring Letters (N=139,576)		Caring Letters (N=87,926)		Total (N=227,502)		Std. diff.
Age in years	53.12	(16.81)	54.44	(17.20)	53.63	(16.97)	-0.077
Age at separation in years	25.97	(6.70)	26.25	(6.69)	26.08	(6.70)	-0.041
Age group							-0.074
Less than 40	38,690	(27.72)	22,883	(26.03)	61,573	(27.06)	
40-54	31,477	(22.55)	18,957	(21.56)	50,434	(22.17)	
55-64	30,956	(22.18)	18,302	(20.82)	49,258	(21.65)	
65-79	31,040	(22.24)	22,345	(25.41)	53,385	(23.47)	
80+	7,413	(5.31)	5,439	(6.19)	12,852	(5.65)	
Sex							0.009
Female	21,644	(15.51)	13,922	(15.83)	35,566	(15.63)	
Male	117932	(84.49)	74,004	(84.17)	191936	(84.37)	
Race							0.004
White	85,262	(61.09)	53,018	(60.30)	138280	(60.78)	
Black or African American	37,358	(26.77)	23,356	(26.56)	60,714	(26.69)	
American Indian or Alaska Native	1,625	(1.16)	977	(1.11)	2,602	(1.14)	
Asian	1,465	(1.05)	1,026	(1.17)	2,491	(1.09)	
More than one race	1,922	(1.38)	1,331	(1.51)	3,253	(1.43)	
Native Hawaiian or Other Pacific Islander	1,388	(0.99)	919	(1.05)	2,307	(1.01)	
Unknown	10,556	(7.56)	7,299	(8.30)	17,855	(7.85)	
Ethnicity							-0.027
Hispanic or Latino	10,745	(7.70)	6,980	(7.94)	17,725	(7.79)	
Not Hispanic or Latino	122289	(87.61)	75,768	(86.17)	198057	(87.06)	
Unknown	6,542	(4.69)	5,178	(5.89)	11,720	(5.15)	
Marital status							0.015
Married	49,804	(35.68)	31,837	(36.21)	81,641	(35.89)	
Single/Never married	32,904	(23.57)	21,181	(24.09)	54,085	(23.77)	
Divorced/Separated/Widowed	55,032	(39.43)	33,567	(38.18)	88,599	(38.94)	
Unknown	1,836	(1.32)	1,341	(1.53)	3,177	(1.40)	
Branch of service							0.000
Air Force	17,321	(12.41)	11,285	(12.83)	28,606	(12.57)	
Army	74,551	(53.41)	46,307	(52.67)	120858	(53.12)	
Navy	27,129	(19.44)	17,290	(19.66)	44,419	(19.52)	
Marine Corps	17,867	(12.80)	11,338	(12.89)	29,205	(12.84)	
Other and missing	2,708	(1.94)	1,706	(1.94)	4,414	(1.94)	
Discharge type							0.004
Honorable	126,070	(90.32)	79,548	(90.47)	205,618	(90.38)	
Dishonorable	974	(0.70)	543	(0.62)	1,517	(0.67)	
Other	10,115	(7.25)	6,326	(7.19)	16,441	(7.23)	
Unknown	2,417	(1.73)	1,509	(1.72)	3,926	(1.73)	
Past suicide-related event	8,925	(6.39)	5,805	(6.60)	14,730	(6.47)	-0.008
Past inpatient mental health stay	12,443	(8.91)	6,489	(7.38)	18,932	(8.32)	0.056
Past outpatient mental health visit	90,593	(64.91)	51,398	(58.46)	141991	(62.41)	0.133
Acquired immune deficiency syndrome	1,279	(0.92)	872	(0.99)	2,151	(0.95)	-0.008
Alcohol disorder	37,586	(26.93)	24,317	(27.66)	61,903	(27.21)	-0.016
Anemias due to other nutritional deficiencies	14,541	(10.42)	10,271	(11.68)	24,812	(10.91)	-0.040
Autoimmune conditions	3,462	(2.48)	2,342	(2.66)	5,804	(2.55)	-0.012
Chronic blood loss (iron deficiency)	1,088	(0.78)	794	(0.90)	1,882	(0.83)	-0.014
Leukemia	375	(0.27)	276	(0.31)	651	(0.29)	-0.008
Lymphoma	641	(0.46)	441	(0.50)	1,082	(0.48)	-0.006
Metastatic cancer	850	(0.61)	592	(0.67)	1,442	(0.63)	-0.008
Solid tumor without metastasis, in situ	3,417	(2.45)	2,480	(2.82)	5,897	(2.59)	-0.023
Solid tumor without metastasis, malignant	6,981	(5.00)	4,974	(5.66)	11,955	(5.25)	-0.029
Cerebrovascular disease - Primary	22,784	(16.32)	15,194	(17.28)	37,978	(16.69)	-0.026
Cerebrovascular disease - Sequela	2,588	(1.85)	1,732	(1.97)	4,320	(1.90)	-0.008
Coagulopathy	4,058	(2.91)	2,563	(2.91)	6,621	(2.91)	-0.000

© 2024 Reger MA et al. *JAMA Network Open.*

Dementia	4,225	(3.03)	2,649	(3.01)	6,874	(3.02)	0.000
Depression	74,801	(53.59)	49,590	(56.40)	124,391	(54.68)	-0.056
Diabetes with chronic complications	18,835	(13.49)	12,825	(14.59)	31,660	(13.92)	-0.031
Diabetes without chronic complications	24,365	(17.46)	16,843	(19.16)	41,208	(18.11)	-0.044
Drug use disorder	31,056	(22.25)	19,920	(22.66)	50,976	(22.41)	-0.010
Fluid and electrolyte disorders	15,377	(11.02)	9,715	(11.05)	25,092	(11.03)	-0.001
Heart failure	8,028	(5.75)	5,532	(6.29)	13,560	(5.96)	-0.023
Homeless	24,238	(17.37)	15,071	(17.14)	39,309	(17.28)	0.006
Hypertension, complicated	11,417	(8.18)	7,504	(8.53)	18,921	(8.32)	-0.013
Hypertension, uncomplicated	57,541	(41.23)	38,882	(44.22)	96,423	(42.38)	-0.061
Liver disease, mild	13,013	(9.32)	8,233	(9.36)	21,246	(9.34)	-0.001
Liver disease and failure, moderate to severe	1,211	(0.87)	785	(0.89)	1,996	(0.88)	-0.003
Liver disease	167	(0.12)	108	(0.12)	275	(0.12)	-0.001
Chronic pulmonary disease	23,783	(17.04)	15,532	(17.66)	39,315	(17.28)	-0.017
Neurological disorders affecting movement	4,052	(2.90)	2,900	(3.30)	6,952	(3.06)	-0.023
Other neurological disorders	8,413	(6.03)	5,538	(6.30)	13,951	(6.13)	-0.011
Seizures and epilepsy	5,586	(4.00)	3,304	(3.76)	8,890	(3.91)	0.013
Obesity	27,694	(19.84)	18,416	(20.94)	46,110	(20.27)	-0.028
Paralysis	2,279	(1.63)	1,387	(1.58)	3,666	(1.61)	0.004
Peripheral vascular disease	9,848	(7.06)	6,736	(7.66)	16,584	(7.29)	-0.023
Psychoses	36,343	(26.04)	24,655	(28.04)	60,998	(26.81)	-0.045
Pulmonary circulation disease	2,224	(1.59)	1,548	(1.76)	3,772	(1.66)	-0.013
Renal (kidney) failure and disease, moderate	7,271	(5.21)	5,406	(6.15)	12,677	(5.57)	-0.041
Renal (kidney) failure and disease, severe	1,712	(1.23)	1,178	(1.34)	2,890	(1.27)	-0.010
Renal failure	1,804	(1.29)	1,404	(1.60)	3,208	(1.41)	-0.026
Hypothyroidism	9,045	(6.48)	6,199	(7.05)	15,244	(6.70)	-0.023
Other thyroid disorders	2,382	(1.71)	1,716	(1.95)	4,098	(1.80)	-0.018
Peptic ulcer with bleeding	1,494	(1.07)	906	(1.03)	2,400	(1.05)	0.004
Valvular disease	3,720	(2.67)	2,435	(2.77)	6,155	(2.71)	-0.006
Weight loss	5,868	(4.20)	4,062	(4.62)	9,930	(4.36)	-0.020

© 2024 Reger MA et al. *JAMA Network Open.*

eTable 8. Rates of All Outcomes for Letters vs No Letters Comparison

Outcome (one year forward)	Total Sample (N=227,502)	No Caring Letters (N=139,576)	Caring Letters (N=87,926)	P-value+
**Any Nonfatal suicide attempt and intentional self-harm**				
At least one event, n (%)	17,703 (7.78)	10,905 (7.81)	6,798 (7.73)	0.480
Frequency, Mean (std. dev)	0.22 (1.55)	0.22 (1.55)	0.22 (1.55)	0.509
**All-cause mortality**				
All-cause mortality, n (%)	9,477 (4.17)	5,607 (4.02)	3,870 (4.40)	0.000
**Any outpatient utilization**				
At least one visit, n (%)	217,095 (95.43)	132,777 (95.13)	84,318 (95.90)	0.000
Visit count, mean (std. dev)	22.74 (22.66)	22.34 (22.44)	23.38 (23.00)	0.000
**Outpatient mental health utilization**				
At least one visit, n (%)	167,191 (73.49)	104,816 (75.10)	62,375 (70.94)	0.000
Visit count, mean (std. dev)	9.50 (15.70)	9.76 (15.78)	9.09 (15.56)	0.000
**Any inpatient utilization**				
At least one visit, n (%)	51,059 (22.44)	32,173 (23.05)	18,886 (21.48)	0.000
Visit count, mean (std. dev)	0.50 (1.38)	0.51 (1.39)	0.48 (1.37)	0.000
**Inpatient mental health utilization**				
At least one visit, n (%)	26,590 (11.69)	17,042 (12.21)	9,548 (10.86)	0.000
Visit count, mean (std. dev)	0.20 (0.74)	0.20 (0.75)	0.19 (0.72)	0.000
**Emergency department utilization**				
At least one visit, n (%)	90,735 (39.88)	56,183 (40.25)	34,552 (39.30)	0.000
Visit count, mean (std. dev)	2.38 (5.29)	2.81 (5.99)	1.71 (3.84)	0.000

Notes: +Chi-squared test was used for categorical variables, Wilcoxon rank-sum test was used for frequencies.

eTable 9. Hazard Ratios from Time-to-Event Analyses of Association between Outcomes and Caring Letters Receipt, Full Sample

VARIABLES	All-Cause Mortality	Suicide Attempt	Inpatient	Inpatient Mental Health	Outpatient	Outpatient Mental Health Care	Emergency Department Use
**Caring Letters Received**	1.067	1.016	**1.127***	**1.137***	**1.105***	**1.195***	**1.107***
	(0.962 - 1.184)	(0.946 - 1.091)	**(1.079 - 1.177)**	**(1.072 - 1.206)**	**(1.084 - 1.127)**	**(1.168 - 1.223)**	**(1.072 - 1.144)**
	0.219	0.670	**0.000**	**0.000**	**0.000**	**0.000**	**0.000**
Call during COVID Period	0.873*	0.892**	0.903***	0.932*	0.799***	0.707***	1.023
	(0.761 - 1.001)	(0.809 - 0.983)	(0.852 - 0.957)	(0.860 - 1.011)	(0.778 - 0.821)	(0.685 - 0.731)	(0.979 - 1.069)
	0.051	0.021	0.001	0.089	0.000	0.000	0.310
Call within prior year	1.138***	1.082***	1.093***	1.093***	0.968***	1.006	1.105***
	(1.075 - 1.204)	(1.043 - 1.122)	(1.069 - 1.117)	(1.062 - 1.125)	(0.957 - 0.979)	(0.993 - 1.018)	(1.086 - 1.124)
	0.000	0.000	0.000	0.000	0.000	0.380	0.000
Age 40-54	1.703***	0.851***	1.081***	0.972	1.019***	0.962***	0.986
	(1.526 - 1.901)	(0.816 - 0.887)	(1.050 - 1.113)	(0.938 - 1.007)	(1.006 - 1.033)	(0.948 - 0.975)	(0.966 - 1.006)
	0.000	0.000	0.000	0.121	0.004	0.000	0.177
Age 55-64	3.005***	0.696***	1.046***	0.896***	0.994	0.835***	0.932***
	(2.714 - 3.327)	(0.663 - 0.731)	(1.015 - 1.079)	(0.862 - 0.931)	(0.980 - 1.008)	(0.822 - 0.848)	(0.911 - 0.953)
	0.000	0.000	0.003	0.000	0.403	0.000	0.000
Age 65-79	4.488***	0.466***	0.969*	0.675***	0.955***	0.630***	0.826***
	(4.056 - 4.965)	(0.438 - 0.495)	(0.937 - 1.001)	(0.643 - 0.707)	(0.941 - 0.969)	(0.619 - 0.641)	(0.805 - 0.847)
	0.000	0.000	0.060	0.000	0.000	0.000	0.000
Age over 80	9.265***	0.210***	0.928***	0.234***	0.903***	0.304***	0.736***
	(8.286 - 10.360)	(0.179 - 0.246)	(0.880 - 0.979)	(0.202 - 0.271)	(0.884 - 0.923)	(0.294 - 0.316)	(0.707 - 0.766)
	0.000	0.000	0.006	0.000	0.000	0.000	0.000
Female	0.564***	0.970	0.808***	0.824***	1.083***	1.078***	0.999
	(0.511 - 0.622)	(0.929 - 1.013)	(0.785 - 0.832)	(0.793 - 0.856)	(1.070 - 1.097)	(1.064 - 1.093)	(0.980 - 1.020)
	0.000	0.175	0.000	0.000	0.000	0.000	0.959
American Indian	1.071	1.037	0.999	0.907	0.943***	0.957*	0.953
	(0.876 - 1.310)	(0.916 - 1.173)	(0.919 - 1.086)	(0.806 - 1.020)	(0.905 - 0.982)	(0.915 - 1.002)	(0.891 - 1.019)
	0.501	0.569	0.987	0.105	0.005	0.059	0.156
Asian	0.726**	1.039	0.833***	0.987	0.986	0.938***	0.924**
	(0.546 - 0.965)	(0.906 - 1.191)	(0.747 - 0.928)	(0.864 - 1.129)	(0.947 - 1.026)	(0.894 - 0.985)	(0.860 - 0.992)
	0.028	0.584	0.001	0.854	0.481	0.010	0.030
Black	0.641***	0.822***	0.960***	0.998	0.998	1.011*	1.172***
	(0.604 - 0.681)	(0.788 - 0.858)	(0.937 - 0.982)	(0.966 - 1.030)	(0.988 - 1.009)	(0.999 - 1.024)	(1.152 - 1.192)
	0.000	0.000	0.001	0.887	0.744	0.076	0.000
More than one race	0.706***	0.921	0.926*	0.937	0.998	1.016	1.034
	(0.559 - 0.893)	(0.815 - 1.040)	(0.854 - 1.004)	(0.842 - 1.042)	(0.963 - 1.034)	(0.975 - 1.058)	(0.975 - 1.096)
	0.004	0.184	0.063	0.227	0.903	0.455	0.262

© 2024 Reger MA et al. *JAMA Network Open.*

Native Hawaiian	0.755**	0.996	0.924	0.934	1.047**	0.983	0.980
	(0.591 - 0.965)	(0.860 - 1.153)	(0.831 - 1.027)	(0.816 - 1.070)	(1.005 - 1.091)	(0.937 - 1.031)	(0.909 - 1.055)
	0.025	0.957	0.141	0.325	0.029	0.479	0.585
Unknown race	0.919*	0.933**	0.947**	0.952*	0.936***	0.930***	1.004
	(0.838 - 1.008)	(0.873 - 0.996)	(0.908 - 0.987)	(0.899 - 1.008)	(0.919 - 0.953)	(0.911 - 0.950)	(0.974 - 1.036)
	0.073	0.037	0.010	0.093	0.000	0.000	0.790
Non-Hispanic	1.145***	0.956	1.044**	1.060**	0.982**	0.967***	0.940***
	(1.035 - 1.268)	(0.903 - 1.013)	(1.004 - 1.085)	(1.006 - 1.117)	(0.966 - 0.999)	(0.948 - 0.986)	(0.914 - 0.966)
	0.009	0.127	0.029	0.028	0.038	0.001	0.000
Unknown Ethnicity	1.258***	1.020	0.992	1.075*	0.860***	0.930***	0.860***
	(1.090 - 1.453)	(0.930 - 1.120)	(0.933 - 1.056)	(0.989 - 1.169)	(0.837 - 0.884)	(0.902 - 0.959)	(0.822 - 0.900)
	0.002	0.670	0.810	0.091	0.000	0.000	0.000
Never Married	1.220***	0.979	1.183***	1.172***	0.980***	0.981***	1.165***
	(1.142 - 1.304)	(0.939 - 1.021)	(1.152 - 1.214)	(1.132 - 1.214)	(0.969 - 0.992)	(0.968 - 0.994)	(1.143 - 1.188)
	0.000	0.325	0.000	0.000	0.001	0.005	0.000
Divorced/Separated/Widowed	1.209***	1.027	1.195***	1.151***	0.995	0.984***	1.167***
	(1.152 - 1.269)	(0.989 - 1.067)	(1.168 - 1.222)	(1.115 - 1.189)	(0.985 - 1.005)	(0.973 - 0.996)	(1.148 - 1.187)
	0.000	0.159	0.000	0.000	0.300	0.008	0.000
Marital Status Missing	1.338**	0.973	0.647***	0.668***	0.787***	0.903***	0.656***
	(1.054 - 1.698)	(0.837 - 1.132)	(0.565 - 0.741)	(0.560 - 0.796)	(0.753 - 0.824)	(0.858 - 0.950)	(0.600 - 0.717)
	0.017	0.723	0.000	0.000	0.000	0.000	0.000
Army	1.001	0.972	0.996	1.003	0.972***	0.958***	0.993
	(0.940 - 1.067)	(0.924 - 1.022)	(0.968 - 1.025)	(0.963 - 1.045)	(0.960 - 0.985)	(0.943 - 0.973)	(0.971 - 1.014)
	0.963	0.264	0.806	0.882	0.000	0.000	0.510
Navy	0.996	0.996	0.983	0.980	0.983**	0.976***	0.992
	(0.926 - 1.071)	(0.941 - 1.055)	(0.951 - 1.016)	(0.936 - 1.027)	(0.968 - 0.998)	(0.959 - 0.994)	(0.968 - 1.017)
	0.919	0.893	0.298	0.401	0.025	0.007	0.525
Marine Corps	0.979	1.024	0.995	0.993	0.962***	0.965***	0.976*
	(0.900 - 1.064)	(0.963 - 1.089)	(0.959 - 1.032)	(0.943 - 1.045)	(0.946 - 0.979)	(0.946 - 0.984)	(0.950 - 1.004)
	0.617	0.454	0.779	0.775	0.000	0.000	0.088
Others and missing	1.089	0.954	0.978	0.993	0.955**	0.982	0.992
	(0.896 - 1.322)	(0.822 - 1.107)	(0.894 - 1.070)	(0.879 - 1.122)	(0.917 - 0.995)	(0.937 - 1.030)	(0.926 - 1.063)
	0.391	0.532	0.631	0.911	0.027	0.455	0.823
Age at separation (Q2)	1.044	0.949**	0.996	0.985	1.008	1.005	0.970***
	(0.989 - 1.102)	(0.909 - 0.992)	(0.972 - 1.021)	(0.951 - 1.019)	(0.996 - 1.020)	(0.991 - 1.019)	(0.951 - 0.988)
	0.122	0.021	0.777	0.384	0.179	0.520	0.001
Age at separation (Q3)	1.027	0.957*	0.998	0.991	1.016**	1.009	0.963***
	(0.968 - 1.090)	(0.916 - 1.001)	(0.973 - 1.024)	(0.956 - 1.026)	(1.004 - 1.028)	(0.995 - 1.024)	(0.945 - 0.982)
	0.377	0.053	0.908	0.604	0.010	0.202	0.000
Age at separation (Q4)	0.986	0.917***	0.940***	0.949***	1.029***	1.036***	0.912***

© 2024 Reger MA et al. *JAMA Network Open.*

	(0.925 - 1.052)	(0.875 - 0.961)	(0.915 - 0.967)	(0.913 - 0.986)	(1.016 - 1.042)	(1.021 - 1.051)	(0.893 - 0.930)
	0.676	0.000	0.000	0.007	0.000	0.000	0.000
Dishonorable	1.027	1.136	0.985	1.039	0.784***	0.879***	1.109**
	(0.746 - 1.413)	(0.942 - 1.370)	(0.869 - 1.117)	(0.894 - 1.207)	(0.736 - 0.835)	(0.823 - 0.940)	(1.010 - 1.217)
	0.872	0.180	0.811	0.620	0.000	0.000	0.029
Other discharge	1.075	1.137***	1.089***	1.101***	0.974***	0.998	1.052***
	(0.974 - 1.185)	(1.080 - 1.197)	(1.053 - 1.127)	(1.056 - 1.148)	(0.957 - 0.990)	(0.980 - 1.016)	(1.025 - 1.080)
	0.150	0.000	0.000	0.000	0.002	0.817	0.000
Unknown discharge	0.976	1.044	0.931	0.929	0.966	0.981	0.941
	(0.777 - 1.224)	(0.892 - 1.222)	(0.839 - 1.034)	(0.804 - 1.074)	(0.925 - 1.009)	(0.932 - 1.033)	(0.874 - 1.013)
	0.832	0.589	0.180	0.321	0.121	0.463	0.107
Acquired immune deficiency syndrome	0.880	1.229***	1.135***	1.161***	1.119***	1.008	1.236***
	(0.707 - 1.095)	(1.083 - 1.396)	(1.054 - 1.222)	(1.054 - 1.278)	(1.076 - 1.164)	(0.962 - 1.056)	(1.167 - 1.310)
	0.251	0.001	0.001	0.002	0.000	0.746	0.000
Alcohol disorder	1.246***	1.207***	1.441***	1.321***	1.018***	1.047***	1.096***
	(1.177 - 1.319)	(1.163 - 1.253)	(1.410 - 1.474)	(1.281 - 1.362)	(1.007 - 1.030)	(1.034 - 1.060)	(1.077 - 1.115)
	0.000	0.000	0.000	0.000	0.001	0.000	0.000
Anemias due to other nutritional deficiencies	1.184***	1.042	1.123***	1.028	1.037***	0.989	1.087***
	(1.118 - 1.254)	(0.988 - 1.100)	(1.093 - 1.154)	(0.988 - 1.070)	(1.023 - 1.052)	(0.972 - 1.006)	(1.063 - 1.111)
	0.000	0.133	0.000	0.171	0.000	0.192	0.000
Autoimmune conditions	0.980	1.019	1.083***	0.937*	1.153***	1.028*	1.163***
	(0.873 - 1.100)	(0.926 - 1.121)	(1.031 - 1.139)	(0.866 - 1.013)	(1.126 - 1.180)	(0.998 - 1.058)	(1.119 - 1.210)
	0.736	0.706	0.002	0.099	0.000	0.070	0.000
Chronic blood loss (iron deficiency)	1.099	0.895	1.076*	0.966	0.998	0.982	1.022
	(0.952 - 1.267)	(0.748 - 1.070)	(0.997 - 1.162)	(0.849 - 1.098)	(0.955 - 1.043)	(0.932 - 1.036)	(0.958 - 1.090)
	0.197	0.223	0.060	0.595	0.925	0.507	0.515
Leukemia	1.339**	0.683*	1.059	0.604***	1.036	1.051	1.018
	(1.069 - 1.678)	(0.442 - 1.056)	(0.910 - 1.233)	(0.441 - 0.827)	(0.959 - 1.120)	(0.955 - 1.158)	(0.902 - 1.148)
	0.011	0.086	0.458	0.002	0.364	0.309	0.778
Lymphoma	1.365***	0.741*	1.147**	0.791**	1.099***	0.930*	1.153***
	(1.136 - 1.639)	(0.548 - 1.003)	(1.027 - 1.282)	(0.638 - 0.980)	(1.039 - 1.163)	(0.862 - 1.003)	(1.056 - 1.259)
	0.001	0.053	0.015	0.032	0.001	0.061	0.002
Metastatic cancer	3.190***	0.923	1.372***	0.854	1.149***	0.858***	1.249***
	(2.828 - 3.597)	(0.705 - 1.209)	(1.242 - 1.516)	(0.694 - 1.051)	(1.091 - 1.211)	(0.798 - 0.922)	(1.153 - 1.354)
	0.000	0.562	0.000	0.137	0.000	0.000	0.000
Solid tumor without metastasis, in situ	1.048	0.944	0.988	0.961	1.065***	0.987	0.998
	(0.954 - 1.151)	(0.823 - 1.083)	(0.932 - 1.047)	(0.869 - 1.064)	(1.037 - 1.094)	(0.953 - 1.022)	(0.954 - 1.044)
	0.328	0.414	0.682	0.447	0.000	0.465	0.926
Solid tumor without metastasis, malignant	1.334***	0.871***	1.153***	0.946	1.112***	0.972**	1.165***
	(1.244 - 1.431)	(0.790 - 0.960)	(1.107 - 1.201)	(0.882 - 1.014)	(1.091 - 1.134)	(0.947 - 0.998)	(1.128 - 1.203)

© 2024 Reger MA et al. *JAMA Network Open.*

	0.000	0.006	0.000	0.114	0.000	0.032	0.000
Cerebrovascular disease - Primary	1.132***	1.021	1.183***	1.059***	1.081***	0.991	1.160***
	(1.074 - 1.194)	(0.976 - 1.068)	(1.155 - 1.212)	(1.023 - 1.096)	(1.068 - 1.094)	(0.977 - 1.006)	(1.138 - 1.182)
	0.000	0.372	0.000	0.001	0.000	0.241	0.000
Cerebrovascular disease - Sequela	0.944	0.977	0.969	0.984	0.986	1.036*	1.012
	(0.828 - 1.077)	(0.855 - 1.117)	(0.907 - 1.035)	(0.887 - 1.091)	(0.952 - 1.021)	(0.994 - 1.081)	(0.960 - 1.068)
	0.393	0.738	0.351	0.759	0.433	0.097	0.649
Coagulopathy	1.276***	0.946	1.218***	1.062*	1.043***	0.993	1.134***
	(1.174 - 1.388)	(0.864 - 1.036)	(1.168 - 1.269)	(0.996 - 1.132)	(1.017 - 1.069)	(0.964 - 1.022)	(1.093 - 1.176)
	0.000	0.235	0.000	0.067	0.001	0.621	0.000
Dementia	1.224***	1.035	1.092***	1.164***	0.945***	1.039**	1.036*
	(1.128 - 1.328)	(0.936 - 1.144)	(1.043 - 1.143)	(1.086 - 1.248)	(0.921 - 0.969)	(1.008 - 1.070)	(0.996 - 1.076)
	0.000	0.503	0.000	0.000	0.000	0.014	0.075
Depression	0.891***	1.225***	1.040***	1.003	1.102***	1.162***	1.066***
	(0.848 - 0.936)	(1.179 - 1.273)	(1.018 - 1.063)	(0.972 - 1.035)	(1.092 - 1.113)	(1.149 - 1.175)	(1.049 - 1.083)
	0.000	0.000	0.000	0.836	0.000	0.000	0.000
Diabetes with chronic complications	1.261***	1.045	1.135***	0.990	1.071***	0.980**	1.134***
	(1.175 - 1.353)	(0.976 - 1.119)	(1.097 - 1.174)	(0.940 - 1.044)	(1.054 - 1.088)	(0.961 - 0.999)	(1.105 - 1.164)
	0.000	0.209	0.000	0.717	0.000	0.042	0.000
Diabetes without chronic complications	1.000	0.973	1.007	0.987	1.047***	0.998	1.012
	(0.935 - 1.069)	(0.915 - 1.034)	(0.976 - 1.039)	(0.942 - 1.034)	(1.032 - 1.061)	(0.980 - 1.015)	(0.988 - 1.036)
	0.999	0.375	0.643	0.585	0.000	0.792	0.345
Drug use disorder	1.025	1.127***	1.356***	1.418***	1.055***	1.065***	1.215***
	(0.962 - 1.092)	(1.083 - 1.173)	(1.325 - 1.388)	(1.373 - 1.465)	(1.042 - 1.067)	(1.051 - 1.079)	(1.193 - 1.237)
	0.441	0.000	0.000	0.000	0.000	0.000	0.000
Fluid and electrolyte disorders	1.280***	1.101***	1.400***	1.154***	1.033***	1.009	1.347***
	(1.206 - 1.358)	(1.049 - 1.156)	(1.365 - 1.437)	(1.113 - 1.196)	(1.018 - 1.048)	(0.992 - 1.026)	(1.319 - 1.376)
	0.000	0.000	0.000	0.000	0.000	0.312	0.000
Heart failure	1.573***	1.040	1.216***	0.945*	1.028***	0.949***	1.094***
	(1.472 - 1.681)	(0.955 - 1.132)	(1.172 - 1.261)	(0.887 - 1.007)	(1.008 - 1.048)	(0.925 - 0.973)	(1.061 - 1.128)
	0.000	0.367	0.000	0.082	0.006	0.000	0.000
Homeless	1.054	1.030	1.235***	1.279***	0.974***	1.061***	1.185***
	(0.989 - 1.124)	(0.991 - 1.071)	(1.207 - 1.264)	(1.241 - 1.318)	(0.962 - 0.986)	(1.047 - 1.076)	(1.164 - 1.207)
	0.108	0.137	0.000	0.000	0.000	0.000	0.000
Hypertension, complicated	0.995	1.071*	1.128***	1.042	1.020**	0.998	1.071***
	(0.929 - 1.066)	(0.996 - 1.151)	(1.090 - 1.167)	(0.988 - 1.099)	(1.002 - 1.038)	(0.977 - 1.021)	(1.041 - 1.101)
	0.886	0.065	0.000	0.132	0.028	0.878	0.000
Hypertension, uncomplicated	1.003	0.958**	1.083***	0.971*	1.106***	0.984***	1.074***
	(0.952 - 1.057)	(0.921 - 0.995)	(1.059 - 1.107)	(0.942 - 1.001)	(1.095 - 1.118)	(0.972 - 0.996)	(1.056 - 1.092)
	0.906	0.028	0.000	0.060	0.000	0.008	0.000

© 2024 Reger MA et al. *JAMA Network Open.*

Liver disease, mild	1.258***	1.019	1.201***	1.098***	1.057***	1.025***	1.167***
	(1.180 - 1.341)	(0.967 - 1.074)	(1.169 - 1.234)	(1.057 - 1.139)	(1.042 - 1.073)	(1.008 - 1.043)	(1.141 - 1.193)
	0.000	0.472	0.000	0.000	0.000	0.004	0.000
Liver disease and failure, moderate to severe	1.717***	0.888	1.035	0.905*	0.991	0.916***	1.024
	(1.510 - 1.952)	(0.755 - 1.045)	(0.964 - 1.111)	(0.807 - 1.015)	(0.947 - 1.037)	(0.869 - 0.966)	(0.961 - 1.091)
	0.000	0.153	0.347	0.087	0.692	0.001	0.468
Liver disease	1.284*	0.866	1.032	0.873	0.923	0.908	0.964
	(0.954 - 1.728)	(0.613 - 1.224)	(0.872 - 1.220)	(0.670 - 1.138)	(0.826 - 1.031)	(0.807 - 1.020)	(0.825 - 1.127)
	0.099	0.415	0.716	0.317	0.157	0.105	0.647
Chronic pulmonary disease	1.239***	1.063***	1.079***	1.025	1.083***	1.026***	1.131***
	(1.180 - 1.301)	(1.019 - 1.109)	(1.055 - 1.104)	(0.992 - 1.059)	(1.071 - 1.095)	(1.012 - 1.040)	(1.111 - 1.151)
	0.000	0.005	0.000	0.136	0.000	0.000	0.000
Neurological disorders affecting movement	0.994	1.077*	1.092***	1.178***	1.071***	1.095***	1.095***
	(0.903 - 1.096)	(0.993 - 1.168)	(1.044 - 1.142)	(1.108 - 1.253)	(1.047 - 1.096)	(1.066 - 1.124)	(1.055 - 1.136)
	0.911	0.073	0.000	0.000	0.000	0.000	0.000
Other neurological disorders	1.037	1.016	1.028*	1.005	1.044***	1.041***	1.078***
	(0.966 - 1.113)	(0.954 - 1.082)	(0.995 - 1.063)	(0.958 - 1.055)	(1.026 - 1.063)	(1.020 - 1.063)	(1.049 - 1.107)
	0.318	0.630	0.098	0.833	0.000	0.000	0.000
Seizures and epilepsy	1.206***	1.087**	1.066***	1.014	1.040***	0.978*	1.041**
	(1.102 - 1.320)	(1.018 - 1.160)	(1.027 - 1.107)	(0.964 - 1.067)	(1.019 - 1.062)	(0.955 - 1.001)	(1.008 - 1.075)
	0.000	0.013	0.001	0.583	0.000	0.064	0.014
Obesity	0.833***	0.972	0.995	0.987	1.111***	1.067***	1.086***
	(0.788 - 0.880)	(0.935 - 1.010)	(0.973 - 1.017)	(0.957 - 1.018)	(1.100 - 1.123)	(1.055 - 1.080)	(1.068 - 1.104)
	0.000	0.151	0.668	0.397	0.000	0.000	0.000
Paralysis	1.142*	1.060	1.113***	0.857***	1.012	0.863***	1.030
	(0.990 - 1.317)	(0.927 - 1.211)	(1.038 - 1.193)	(0.769 - 0.956)	(0.973 - 1.052)	(0.825 - 0.903)	(0.974 - 1.090)
	0.068	0.395	0.003	0.005	0.561	0.000	0.304
Peripheral vascular disease	1.131***	0.952	1.122***	0.902***	1.105***	0.994	1.129***
	(1.066 - 1.200)	(0.886 - 1.022)	(1.088 - 1.158)	(0.855 - 0.952)	(1.088 - 1.123)	(0.974 - 1.014)	(1.101 - 1.159)
	0.000	0.175	0.000	0.000	0.000	0.547	0.000
Psychoses	1.008	1.187***	1.216***	1.539***	1.070***	1.184***	1.141***
	(0.954 - 1.064)	(1.146 - 1.229)	(1.191 - 1.242)	(1.496 - 1.584)	(1.059 - 1.081)	(1.171 - 1.197)	(1.123 - 1.160)
	0.780	0.000	0.000	0.000	0.000	0.000	0.000
Pulmonary circulation disease	1.260***	1.081	1.149***	1.001	1.063***	1.023	1.136***
	(1.143 - 1.389)	(0.958 - 1.220)	(1.087 - 1.214)	(0.912 - 1.099)	(1.030 - 1.097)	(0.985 - 1.063)	(1.084 - 1.190)
	0.000	0.205	0.000	0.982	0.000	0.232	0.000
Renal (kidney) failure and disease, moderate	1.023	0.948	1.027	0.986	1.028***	0.999	1.010
	(0.951 - 1.102)	(0.867 - 1.037)	(0.987 - 1.068)	(0.923 - 1.053)	(1.009 - 1.048)	(0.975 - 1.025)	(0.978 - 1.042)
	0.540	0.245	0.188	0.672	0.005	0.962	0.547
Renal (kidney) failure and	1.983***	0.645***	1.249***	0.654***	1.061***	0.903***	1.078***

© 2024 Reger MA et al. *JAMA Network Open.*

disease, severe	(1.789 - 2.198)	(0.521 - 0.798)	(1.170 - 1.332)	(0.558 - 0.765)	(1.020 - 1.104)	(0.859 - 0.950)	(1.020 - 1.140)
	0.000	0.000	0.000	0.000	0.003	0.000	0.008
Renal failure	0.896*	1.019	0.976	0.995	1.031*	1.020	1.036
	(0.792 - 1.014)	(0.883 - 1.176)	(0.916 - 1.039)	(0.897 - 1.105)	(0.996 - 1.066)	(0.978 - 1.063)	(0.984 - 1.090)
	0.082	0.799	0.445	0.931	0.081	0.356	0.176
Hypothyroidism	0.990	1.057*	1.045***	1.076***	1.053***	1.035***	1.014
	(0.924 - 1.060)	(0.993 - 1.124)	(1.011 - 1.080)	(1.025 - 1.130)	(1.037 - 1.070)	(1.015 - 1.055)	(0.987 - 1.041)
	0.768	0.080	0.010	0.003	0.000	0.001	0.308
Other thyroid disorders	0.892	0.909	1.014	0.873***	1.070***	1.014	1.114***
	(0.775 - 1.028)	(0.804 - 1.026)	(0.955 - 1.076)	(0.796 - 0.958)	(1.039 - 1.103)	(0.979 - 1.050)	(1.065 - 1.166)
	0.114	0.123	0.651	0.004	0.000	0.442	0.000
Peptic ulcer with bleeding	1.124*	1.262***	1.093**	1.080	1.023	1.020	1.137***
	(0.982 - 1.287)	(1.115 - 1.427)	(1.021 - 1.170)	(0.980 - 1.189)	(0.984 - 1.064)	(0.976 - 1.067)	(1.074 - 1.203)
	0.090	0.000	0.010	0.121	0.251	0.380	0.000
Valvular disease	1.119***	0.957	1.069***	0.962	1.058***	0.975	1.072***
	(1.032 - 1.213)	(0.858 - 1.068)	(1.020 - 1.121)	(0.886 - 1.044)	(1.031 - 1.085)	(0.944 - 1.006)	(1.030 - 1.114)
	0.007	0.438	0.006	0.348	0.000	0.116	0.001
Weight loss	1.388***	1.085**	1.114***	1.028	1.051***	0.997	1.081***
	(1.295 - 1.488)	(1.010 - 1.166)	(1.075 - 1.154)	(0.975 - 1.084)	(1.030 - 1.072)	(0.974 - 1.021)	(1.049 - 1.115)
	0.000	0.026	0.000	0.309	0.000	0.826	0.000
Past-year inpatient mental health care use	0.877***	1.325***	1.643***	2.296***	1.047***	1.135***	1.387***
	(0.802 - 0.959)	(1.265 - 1.388)	(1.598 - 1.690)	(2.218 - 2.378)	(1.029 - 1.065)	(1.115 - 1.156)	(1.355 - 1.420)
	0.004	0.000	0.000	0.000	0.000	0.000	0.000
Past-year outpatient mental health care use	0.856***	1.215***	1.197***	1.411***	1.435***	2.065***	1.268***
	(0.811 - 0.903)	(1.162 - 1.270)	(1.167 - 1.228)	(1.356 - 1.469)	(1.420 - 1.451)	(2.038 - 2.093)	(1.245 - 1.292)
	0.000	0.000	0.000	0.000	0.000	0.000	0.000
Past-year suicide attempt	1.042	4.737***	1.501***	1.704***	1.266***	1.392***	1.198***
	(0.942 - 1.152)	(4.551 - 4.931)	(1.455 - 1.548)	(1.642 - 1.767)	(1.243 - 1.289)	(1.366 - 1.418)	(1.168 - 1.229)
	0.426	0.000	0.000	0.000	0.000	0.000	0.000
2019	0.952	1.064**	0.885***	0.898***	0.989	0.929***	0.876***
	(0.886 - 1.024)	(1.011 - 1.120)	(0.860 - 0.912)	(0.863 - 0.935)	(0.974 - 1.003)	(0.914 - 0.944)	(0.857 - 0.896)
	0.186	0.018	0.000	0.000	0.123	0.000	0.000
2020	1.047	1.166***	0.787***	0.781***	0.957***	0.823***	0.750***
	(0.912 - 1.203)	(1.058 - 1.286)	(0.743 - 0.834)	(0.721 - 0.845)	(0.931 - 0.985)	(0.797 - 0.851)	(0.718 - 0.783)
	0.513	0.002	0.000	0.000	0.002	0.000	0.000
2021	1.053	1.215***	0.801***	0.763***	1.108***	0.926***	0.744***
	(0.866 - 1.280)	(1.058 - 1.395)	(0.738 - 0.870)	(0.681 - 0.854)	(1.065 - 1.153)	(0.884 - 0.969)	(0.700 - 0.792)
	0.606	0.006	0.000	0.000	0.000	0.001	0.000
February	1.119**	1.052	1.002	0.974	1.027**	1.018	1.020

© 2024 Reger MA et al. *JAMA Network Open.*

	(1.008 - 1.242)	(0.976 - 1.134)	(0.959 - 1.048)	(0.916 - 1.035)	(1.005 - 1.048)	(0.993 - 1.043)	(0.987 - 1.054)
	0.035	0.188	0.918	0.396	0.013	0.162	0.247
March	1.112*	1.029	1.016	0.984	1.032***	1.074***	0.974
	(0.993 - 1.244)	(0.947 - 1.118)	(0.968 - 1.065)	(0.921 - 1.052)	(1.010 - 1.055)	(1.047 - 1.102)	(0.940 - 1.009)
	0.066	0.502	0.520	0.643	0.005	0.000	0.146
April	1.100	1.068	1.028	0.993	1.018	1.050***	1.011
	(0.980 - 1.234)	(0.983 - 1.161)	(0.979 - 1.078)	(0.929 - 1.062)	(0.995 - 1.041)	(1.023 - 1.078)	(0.975 - 1.048)
	0.107	0.121	0.267	0.847	0.126	0.000	0.554
May	1.136**	1.084*	1.057**	1.017	1.022*	1.092***	1.039**
	(1.013 - 1.274)	(0.998 - 1.177)	(1.008 - 1.109)	(0.952 - 1.086)	(1.000 - 1.045)	(1.065 - 1.121)	(1.003 - 1.076)
	0.029	0.055	0.021	0.618	0.050	0.000	0.035
June	1.164***	1.045	0.962	0.924**	1.005	1.011	0.949***
	(1.038 - 1.306)	(0.962 - 1.135)	(0.916 - 1.010)	(0.865 - 0.988)	(0.983 - 1.028)	(0.984 - 1.037)	(0.915 - 0.984)
	0.009	0.295	0.115	0.021	0.649	0.434	0.005
July	1.114*	1.056	0.931***	0.893***	1.011	0.979	0.934***
	(0.986 - 1.258)	(0.968 - 1.152)	(0.885 - 0.979)	(0.833 - 0.957)	(0.987 - 1.036)	(0.952 - 1.006)	(0.899 - 0.970)
	0.083	0.217	0.005	0.001	0.368	0.129	0.000
August	1.051	1.061	0.924***	0.914**	1.001	0.969**	0.934***
	(0.929 - 1.188)	(0.972 - 1.157)	(0.878 - 0.972)	(0.853 - 0.979)	(0.977 - 1.025)	(0.943 - 0.997)	(0.900 - 0.970)
	0.429	0.183	0.002	0.011	0.930	0.028	0.000
September	1.075	1.056	0.931***	0.905***	1.023*	0.981	0.916***
	(0.950 - 1.217)	(0.967 - 1.153)	(0.885 - 0.980)	(0.844 - 0.971)	(0.998 - 1.048)	(0.954 - 1.009)	(0.882 - 0.952)
	0.252	0.227	0.007	0.005	0.069	0.181	0.000
October	1.146**	1.079*	0.908***	0.882***	1.012	0.975*	0.898***
	(1.015 - 1.294)	(0.989 - 1.178)	(0.863 - 0.956)	(0.822 - 0.946)	(0.988 - 1.036)	(0.948 - 1.003)	(0.864 - 0.933)
	0.028	0.086	0.000	0.000	0.349	0.078	0.000
November	1.147**	1.023	0.912***	0.871***	1.003	0.960***	0.915***
	(1.013 - 1.298)	(0.935 - 1.119)	(0.865 - 0.960)	(0.811 - 0.936)	(0.979 - 1.028)	(0.933 - 0.987)	(0.880 - 0.952)
	0.031	0.619	0.001	0.000	0.798	0.004	0.000
December	1.064	1.081*	0.909***	0.840***	0.978*	0.933***	0.917***
	(0.938 - 1.207)	(0.990 - 1.182)	(0.862 - 0.957)	(0.781 - 0.902)	(0.954 - 1.002)	(0.907 - 0.960)	(0.882 - 0.953)
	0.332	0.084	0.000	0.000	0.071	0.000	0.000
Observations	225,609	225,609	225,609	225,609	225,609	225,609	225,609
AIC	215994	415385	1.204e+06	621335	4.929e+06	3.863e+06	2.146e+06
BIC	218741	418132	1.207e+06	624071	4.932e+06	3.866e+06	2.148e+06

Notes: *** p<0.01, ** p<0.05, * p<0.1; Coefficients are hazard ratios with 95% CI in parentheses, followed by p-value; Standard errors were clustered on patienticn level to account for multiple observations per individual; Excluded those with age at separation missing due to small cell size.

© 2024 Reger MA et al. *JAMA Network Open.*

eTable 10. Hazard Ratios from Time-to-Event Analyses of Association Between Outcomes and Caring Letters Receipt, Presumed Completers

VARIABLES	All-Cause Mortality	Suicide Attempt	Inpatient	Inpatient Mental Health	Outpatient	Outpatient Mental Health Care	Emergency Department Use
**Caring Letters Received**	1.018	0.996	**1.107*** **	**1.095*** **	**1.111*** **	**1.198*** **	**1.094*** **
	(0.908 - 1.141)	(0.926 - 1.071)	**(1.059 - 1.157)**	**(1.031 - 1.163)**	**(1.089 - 1.133)**	**(1.171 - 1.227)**	**(1.059 - 1.130)**
	0.759	0.915	**0.000**	**0.003**	**0.000**	**0.000**	**0.000**
Call during COVID Period	0.883	0.890**	0.907***	0.936	0.801***	0.707***	1.023
	(0.759 - 1.028)	(0.806 - 0.983)	(0.854 - 0.962)	(0.862 - 1.016)	(0.779 - 0.823)	(0.685 - 0.731)	(0.978 - 1.069)
	0.108	0.021	0.001	0.112	0.000	0.000	0.321
Call within prior year	1.173***	1.081***	1.097***	1.093***	0.969***	1.006	1.106***
	(1.101 - 1.249)	(1.042 - 1.122)	(1.073 - 1.122)	(1.061 - 1.125)	(0.958 - 0.980)	(0.993 - 1.019)	(1.086 - 1.125)
	0.000	0.000	0.000	0.000	0.000	0.350	0.000
Age 40-54	1.738***	0.845***	1.081***	0.969*	1.023***	0.962***	0.988
	(1.537 - 1.964)	(0.809 - 0.882)	(1.050 - 1.114)	(0.934 - 1.005)	(1.009 - 1.036)	(0.949 - 0.976)	(0.967 - 1.009)
	0.000	0.000	0.000	0.086	0.001	0.000	0.267
Age 55-64	2.984***	0.689***	1.042**	0.889***	0.995	0.834***	0.932***
	(2.661 - 3.346)	(0.656 - 0.724)	(1.010 - 1.074)	(0.854 - 0.925)	(0.981 - 1.009)	(0.821 - 0.847)	(0.911 - 0.953)
	0.000	0.000	0.010	0.000	0.466	0.000	0.000
Age 65-79	4.581***	0.458***	0.962**	0.665***	0.957***	0.629***	0.824***
	(4.089 - 5.133)	(0.430 - 0.487)	(0.930 - 0.995)	(0.633 - 0.698)	(0.943 - 0.972)	(0.618 - 0.640)	(0.803 - 0.845)
	0.000	0.000	0.024	0.000	0.000	0.000	0.000
Age over 80	9.836***	0.195***	0.919***	0.234***	0.902***	0.303***	0.734***
	(8.679 - 11.147)	(0.165 - 0.230)	(0.870 - 0.971)	(0.202 - 0.271)	(0.882 - 0.922)	(0.292 - 0.314)	(0.704 - 0.764)
	0.000	0.000	0.002	0.000	0.000	0.000	0.000
Female	0.569***	0.983	0.808***	0.823***	1.083***	1.077***	1.000
	(0.510 - 0.636)	(0.941 - 1.028)	(0.785 - 0.833)	(0.791 - 0.856)	(1.069 - 1.096)	(1.062 - 1.091)	(0.980 - 1.021)
	0.000	0.450	0.000	0.000	0.000	0.000	1.000
American Indian	1.000	1.055	0.991	0.903	0.941***	0.957*	0.950
	(0.793 - 1.261)	(0.929 - 1.197)	(0.910 - 1.080)	(0.800 - 1.020)	(0.903 - 0.981)	(0.914 - 1.002)	(0.887 - 1.017)
	1.000	0.412	0.845	0.101	0.004	0.062	0.137
Asian	0.712**	1.053	0.834***	0.978	0.983	0.942**	0.925**
	(0.517 - 0.979)	(0.917 - 1.210)	(0.746 - 0.931)	(0.853 - 1.121)	(0.944 - 1.024)	(0.897 - 0.989)	(0.860 - 0.995)
	0.037	0.465	0.001	0.745	0.409	0.017	0.036
Black	0.648***	0.822***	0.957***	0.990	0.998	1.011*	1.175***
	(0.606 - 0.693)	(0.787 - 0.859)	(0.934 - 0.981)	(0.958 - 1.023)	(0.987 - 1.009)	(0.998 - 1.024)	(1.154 - 1.196)
	0.000	0.000	0.000	0.531	0.679	0.091	0.000
More than one race	0.742**	0.912	0.917**	0.938	1.001	1.016	1.028
	(0.574 - 0.957)	(0.804 - 1.035)	(0.844 - 0.996)	(0.842 - 1.046)	(0.965 - 1.038)	(0.975 - 1.059)	(0.968 - 1.091)
	0.022	0.152	0.040	0.248	0.967	0.459	0.366
Native Hawaiian	0.754**	1.018	0.914*	0.899	1.047**	0.982	0.970
	(0.575 - 0.989)	(0.877 - 1.181)	(0.825 - 1.013)	(0.780 - 1.037)	(1.004 - 1.091)	(0.935 - 1.031)	(0.899 - 1.046)
	0.041	0.819	0.087	0.145	0.033	0.464	0.424
Unknown race	0.941	0.929**	0.946**	0.952	0.937***	0.929***	1.002
	(0.850 - 1.042)	(0.869 - 0.994)	(0.907 - 0.988)	(0.898 - 1.010)	(0.919 - 0.954)	(0.909 - 0.950)	(0.971 - 1.034)
	0.244	0.033	0.011	0.104	0.000	0.000	0.903
Non-Hispanic	1.140**	0.956	1.042**	1.054*	0.982**	0.967***	0.938***

© 2024 Reger MA et al. *JAMA Network Open.*

	(1.020 - 1.274)	(0.901 - 1.014)	(1.001 - 1.083)	(1.000 - 1.112)	(0.965 - 0.999)	(0.948 - 0.986)	(0.912 - 0.965)
	0.021	0.133	0.042	0.051	0.038	0.001	0.000
Unknown Ethnicity	1.222**	1.023	0.967	1.050	0.855***	0.924***	0.852***
	(1.042 - 1.432)	(0.929 - 1.126)	(0.907 - 1.031)	(0.963 - 1.144)	(0.832 - 0.879)	(0.896 - 0.954)	(0.814 - 0.892)
	0.014	0.649	0.305	0.272	0.000	0.000	0.000
Never Married	1.240***	0.973	1.172***	1.159***	0.983***	0.981***	1.165***
	(1.151 - 1.335)	(0.932 - 1.016)	(1.141 - 1.204)	(1.118 - 1.202)	(0.971 - 0.994)	(0.967 - 0.994)	(1.143 - 1.188)
	0.000	0.218	0.000	0.000	0.004	0.005	0.000
Divorced/Separated/Widowed	1.214***	1.027	1.192***	1.148***	0.996	0.987**	1.168***
	(1.150 - 1.281)	(0.989 - 1.068)	(1.165 - 1.219)	(1.111 - 1.186)	(0.986 - 1.006)	(0.975 - 0.998)	(1.148 - 1.188)
	0.000	0.168	0.000	0.000	0.424	0.024	0.000
Marital Status Missing	1.359**	0.942	0.633***	0.647***	0.789***	0.906***	0.653***
	(1.041 - 1.773)	(0.805 - 1.101)	(0.550 - 0.729)	(0.539 - 0.776)	(0.753 - 0.825)	(0.861 - 0.954)	(0.596 - 0.715)
	0.024	0.452	0.000	0.000	0.000	0.000	0.000
Army	1.017	0.982	0.998	0.999	0.973***	0.959***	0.996
	(0.948 - 1.091)	(0.932 - 1.034)	(0.969 - 1.027)	(0.958 - 1.042)	(0.961 - 0.986)	(0.944 - 0.974)	(0.974 - 1.018)
	0.638	0.489	0.877	0.973	0.000	0.000	0.696
Navy	1.004	1.004	0.984	0.974	0.983**	0.977**	0.994
	(0.926 - 1.089)	(0.946 - 1.064)	(0.951 - 1.017)	(0.929 - 1.022)	(0.968 - 0.998)	(0.960 - 0.995)	(0.969 - 1.019)
	0.923	0.901	0.343	0.285	0.024	0.012	0.635
Marine Corps	0.982	1.032	0.998	0.988	0.962***	0.964***	0.981
	(0.894 - 1.077)	(0.969 - 1.100)	(0.961 - 1.035)	(0.938 - 1.041)	(0.946 - 0.979)	(0.945 - 0.983)	(0.954 - 1.009)
	0.695	0.323	0.900	0.652	0.000	0.000	0.174
Others and missing	1.094	0.954	0.970	0.985	0.953**	0.977	0.994
	(0.882 - 1.357)	(0.818 - 1.112)	(0.885 - 1.063)	(0.870 - 1.115)	(0.914 - 0.993)	(0.931 - 1.026)	(0.927 - 1.066)
	0.413	0.547	0.509	0.815	0.022	0.353	0.871
Age at separation (Q2)	1.060*	0.944**	0.992	0.981	1.011*	1.006	0.972***
	(0.997 - 1.126)	(0.902 - 0.988)	(0.967 - 1.017)	(0.946 - 1.016)	(0.999 - 1.023)	(0.992 - 1.021)	(0.953 - 0.991)
	0.061	0.013	0.521	0.283	0.076	0.393	0.004
Age at separation (Q3)	1.019	0.956**	0.997	0.990	1.018***	1.012	0.965***
	(0.955 - 1.088)	(0.913 - 1.000)	(0.972 - 1.023)	(0.955 - 1.026)	(1.006 - 1.031)	(0.997 - 1.026)	(0.947 - 0.985)
	0.567	0.049	0.828	0.566	0.003	0.108	0.000
Age at separation (Q4)	0.974	0.915***	0.938***	0.944***	1.030***	1.037***	0.912***
	(0.907 - 1.047)	(0.872 - 0.960)	(0.912 - 0.964)	(0.908 - 0.982)	(1.017 - 1.043)	(1.022 - 1.053)	(0.893 - 0.932)
	0.477	0.000	0.000	0.004	0.000	0.000	0.000
Dishonorable	1.041	1.144	0.985	1.043	0.783***	0.881***	1.107**
	(0.733 - 1.481)	(0.942 - 1.389)	(0.865 - 1.121)	(0.892 - 1.219)	(0.734 - 0.836)	(0.823 - 0.944)	(1.006 - 1.218)
	0.821	0.176	0.817	0.598	0.000	0.000	0.037
Other discharge	1.115**	1.123***	1.090***	1.101***	0.972***	0.996	1.056***
	(1.000 - 1.242)	(1.064 - 1.184)	(1.053 - 1.129)	(1.055 - 1.150)	(0.955 - 0.989)	(0.978 - 1.016)	(1.028 - 1.085)
	0.049	0.000	0.000	0.000	0.001	0.716	0.000
Unknown discharge	0.935	1.033	0.936	0.938	0.969	0.978	0.945
	(0.723 - 1.210)	(0.878 - 1.216)	(0.842 - 1.041)	(0.809 - 1.088)	(0.927 - 1.013)	(0.929 - 1.031)	(0.876 - 1.018)
	0.611	0.696	0.222	0.399	0.160	0.413	0.137
Acquired immune deficiency	0.883	1.237***	1.125***	1.159***	1.120***	1.011	1.233***
syndrome	(0.695 - 1.123)	(1.083 - 1.414)	(1.042 - 1.216)	(1.048 - 1.281)	(1.075 - 1.167)	(0.963 - 1.061)	(1.161 - 1.309)
	0.312	0.002	0.003	0.004	0.000	0.656	0.000

© 2024 Reger MA et al. *JAMA Network Open.*

Alcohol disorder	1.264***	1.206***	1.441***	1.318***	1.017***	1.045***	1.093***
	(1.187 - 1.346)	(1.161 - 1.253)	(1.409 - 1.474)	(1.277 - 1.360)	(1.006 - 1.029)	(1.032 - 1.058)	(1.074 - 1.112)
	0.000	0.000	0.000	0.000	0.002	0.000	0.000
Anemias due to other	1.198***	1.036	1.124***	1.028	1.037***	0.987	1.087***
nutritional deficiencies	(1.124 - 1.276)	(0.980 - 1.096)	(1.093 - 1.155)	(0.986 - 1.070)	(1.022 - 1.052)	(0.970 - 1.004)	(1.063 - 1.112)
	0.000	0.210	0.000	0.193	0.000	0.140	0.000
Autoimmune conditions	0.904	1.015	1.085***	0.937	1.155***	1.029*	1.165***
	(0.792 - 1.032)	(0.920 - 1.119)	(1.031 - 1.141)	(0.865 - 1.015)	(1.128 - 1.183)	(0.999 - 1.060)	(1.120 - 1.212)
	0.134	0.771	0.002	0.112	0.000	0.060	0.000
Chronic blood loss (iron	1.111	0.865	1.092**	0.970	0.997	0.984	1.018
deficiency)	(0.947 - 1.303)	(0.720 - 1.040)	(1.011 - 1.180)	(0.848 - 1.110)	(0.953 - 1.043)	(0.932 - 1.038)	(0.953 - 1.088)
	0.197	0.123	0.025	0.658	0.893	0.549	0.590
Leukemia	1.329**	0.624*	1.074	0.609***	1.036	1.069	1.025
	(1.035 - 1.706)	(0.388 - 1.004)	(0.920 - 1.254)	(0.440 - 0.843)	(0.957 - 1.122)	(0.971 - 1.177)	(0.906 - 1.159)
	0.026	0.052	0.367	0.003	0.377	0.174	0.695
Lymphoma	1.327***	0.705**	1.130**	0.769**	1.098***	0.934*	1.147***
	(1.080 - 1.629)	(0.513 - 0.969)	(1.008 - 1.267)	(0.614 - 0.963)	(1.037 - 1.163)	(0.865 - 1.008)	(1.048 - 1.256)
	0.007	0.031	0.036	0.022	0.001	0.080	0.003
Metastatic cancer	2.927***	0.928	1.336***	0.850	1.158***	0.870***	1.234***
	(2.549 - 3.362)	(0.705 - 1.222)	(1.204 - 1.482)	(0.687 - 1.051)	(1.096 - 1.223)	(0.808 - 0.937)	(1.136 - 1.340)
	0.000	0.595	0.000	0.132	0.000	0.000	0.000
Solid tumor without	1.034	0.963	0.988	0.971	1.066***	0.991	0.999
metastasis, in situ	(0.930 - 1.149)	(0.837 - 1.108)	(0.931 - 1.048)	(0.875 - 1.077)	(1.037 - 1.095)	(0.956 - 1.027)	(0.955 - 1.046)
	0.536	0.598	0.681	0.575	0.000	0.618	0.982
Solid tumor without	1.301***	0.863***	1.155***	0.942*	1.110***	0.973**	1.168***
metastasis, malignant	(1.203 - 1.407)	(0.780 - 0.954)	(1.109 - 1.204)	(0.877 - 1.011)	(1.088 - 1.132)	(0.948 - 0.999)	(1.130 - 1.206)
	0.000	0.004	0.000	0.098	0.000	0.039	0.000
Cerebrovascular disease -	1.136***	1.021	1.186***	1.063***	1.081***	0.990	1.161***
Primary	(1.071 - 1.204)	(0.975 - 1.070)	(1.157 - 1.215)	(1.025 - 1.101)	(1.068 - 1.095)	(0.975 - 1.005)	(1.138 - 1.183)
	0.000	0.377	0.000	0.001	0.000	0.176	0.000
Cerebrovascular disease -	0.912	0.987	0.963	0.998	0.985	1.040*	1.010
Sequela	(0.789 - 1.055)	(0.861 - 1.133)	(0.900 - 1.030)	(0.898 - 1.110)	(0.950 - 1.022)	(0.996 - 1.085)	(0.956 - 1.066)
	0.214	0.857	0.267	0.977	0.428	0.073	0.726
Coagulopathy	1.321***	0.959	1.226***	1.069**	1.040***	0.990	1.135***
	(1.204 - 1.448)	(0.873 - 1.054)	(1.176 - 1.279)	(1.000 - 1.141)	(1.014 - 1.066)	(0.960 - 1.020)	(1.093 - 1.178)
	0.000	0.387	0.000	0.048	0.003	0.492	0.000
Dementia	1.266***	1.030	1.095***	1.171***	0.947***	1.043***	1.038*
	(1.158 - 1.384)	(0.930 - 1.142)	(1.045 - 1.147)	(1.090 - 1.257)	(0.923 - 0.972)	(1.012 - 1.075)	(0.998 - 1.079)
	0.000	0.569	0.000	0.000	0.000	0.007	0.066
Depression	0.893***	1.217***	1.039***	1.000	1.100***	1.159***	1.066***
	(0.845 - 0.944)	(1.170 - 1.266)	(1.015 - 1.062)	(0.968 - 1.032)	(1.089 - 1.111)	(1.146 - 1.173)	(1.048 - 1.083)
	0.000	0.000	0.001	0.982	0.000	0.000	0.000
Diabetes with chronic	1.271***	1.044	1.135***	0.990	1.069***	0.980**	1.130***
complications	(1.175 - 1.375)	(0.973 - 1.120)	(1.097 - 1.175)	(0.938 - 1.045)	(1.052 - 1.086)	(0.961 - 1.000)	(1.101 - 1.161)
	0.000	0.231	0.000	0.721	0.000	0.047	0.000
Diabetes without chronic	1.008	0.971	1.006	0.979	1.046***	0.997	1.013
complications	(0.936 - 1.087)	(0.912 - 1.034)	(0.975 - 1.039)	(0.933 - 1.027)	(1.031 - 1.061)	(0.980 - 1.015)	(0.989 - 1.038)

© 2024 Reger MA et al. *JAMA Network Open.*

	0.829	0.364	0.698	0.392	0.000	0.779	0.289
Drug use disorder	1.045	1.128***	1.356***	1.414***	1.054***	1.064***	1.214***
	(0.974 - 1.121)	(1.083 - 1.175)	(1.324 - 1.389)	(1.369 - 1.462)	(1.041 - 1.067)	(1.050 - 1.079)	(1.192 - 1.237)
	0.219	0.000	0.000	0.000	0.000	0.000	0.000
Fluid and electrolyte disorders	1.275***	1.117***	1.406***	1.160***	1.033***	1.012	1.350***
	(1.194 - 1.363)	(1.062 - 1.174)	(1.370 - 1.443)	(1.118 - 1.205)	(1.018 - 1.048)	(0.995 - 1.029)	(1.321 - 1.380)
	0.000	0.000	0.000	0.000	0.000	0.168	0.000
Heart failure	1.544***	1.043	1.215***	0.948	1.034***	0.953***	1.097***
	(1.434 - 1.663)	(0.956 - 1.139)	(1.171 - 1.261)	(0.888 - 1.012)	(1.013 - 1.054)	(0.929 - 0.978)	(1.064 - 1.132)
	0.000	0.343	0.000	0.109	0.001	0.000	0.000
Homeless	1.122***	1.015	1.222***	1.255***	0.976***	1.059***	1.173***
	(1.045 - 1.205)	(0.974 - 1.057)	(1.194 - 1.251)	(1.217 - 1.294)	(0.963 - 0.988)	(1.045 - 1.074)	(1.151 - 1.195)
	0.002	0.478	0.000	0.000	0.000	0.000	0.000
Hypertension, complicated	1.002	1.065*	1.134***	1.042	1.022**	0.996	1.074***
	(0.928 - 1.082)	(0.989 - 1.148)	(1.096 - 1.174)	(0.987 - 1.101)	(1.004 - 1.041)	(0.974 - 1.019)	(1.044 - 1.105)
	0.957	0.097	0.000	0.139	0.015	0.752	0.000
Hypertension, uncomplicated	1.005	0.957**	1.083***	0.972*	1.107***	0.983***	1.074***
	(0.948 - 1.066)	(0.920 - 0.996)	(1.059 - 1.108)	(0.942 - 1.002)	(1.096 - 1.118)	(0.971 - 0.995)	(1.055 - 1.092)
	0.864	0.031	0.000	0.071	0.000	0.006	0.000
Liver disease, mild	1.259***	1.022	1.208***	1.101***	1.055***	1.025***	1.166***
	(1.174 - 1.351)	(0.968 - 1.079)	(1.175 - 1.241)	(1.060 - 1.145)	(1.039 - 1.071)	(1.007 - 1.043)	(1.140 - 1.193)
	0.000	0.433	0.000	0.000	0.000	0.006	0.000
Liver disease and failure,	1.672***	0.899	1.030	0.922	0.988	0.921***	1.026
moderate to severe	(1.447 - 1.932)	(0.760 - 1.063)	(0.958 - 1.107)	(0.820 - 1.037)	(0.943 - 1.036)	(0.872 - 0.972)	(0.962 - 1.095)
	0.000	0.214	0.432	0.175	0.619	0.003	0.432
Liver disease	1.196	0.908	1.042	0.926	0.902*	0.921	0.959
	(0.853 - 1.677)	(0.638 - 1.292)	(0.877 - 1.239)	(0.703 - 1.219)	(0.804 - 1.012)	(0.817 - 1.038)	(0.816 - 1.126)
	0.299	0.590	0.639	0.583	0.078	0.178	0.606
Chronic pulmonary disease	1.253***	1.050**	1.075***	1.016	1.080***	1.023***	1.133***
	(1.187 - 1.322)	(1.006 - 1.097)	(1.050 - 1.100)	(0.983 - 1.051)	(1.068 - 1.092)	(1.010 - 1.037)	(1.112 - 1.153)
	0.000	0.027	0.000	0.345	0.000	0.001	0.000
Neurological disorders affecting	1.010	1.071	1.093***	1.172***	1.077***	1.098***	1.093***
movement	(0.907 - 1.124)	(0.985 - 1.164)	(1.044 - 1.144)	(1.100 - 1.249)	(1.053 - 1.102)	(1.069 - 1.128)	(1.053 - 1.134)
	0.859	0.109	0.000	0.000	0.000	0.000	0.000
Other neurological disorders	1.054	1.026	1.029*	1.010	1.044***	1.038***	1.078***
	(0.975 - 1.140)	(0.961 - 1.094)	(0.995 - 1.065)	(0.961 - 1.061)	(1.025 - 1.063)	(1.016 - 1.060)	(1.048 - 1.108)
	0.185	0.443	0.095	0.704	0.000	0.001	0.000
Seizures and epilepsy	1.234***	1.090**	1.069***	1.014	1.042***	0.979*	1.047***
	(1.117 - 1.363)	(1.019 - 1.167)	(1.028 - 1.111)	(0.962 - 1.068)	(1.019 - 1.064)	(0.956 - 1.003)	(1.013 - 1.082)
	0.000	0.012	0.001	0.601	0.000	0.088	0.006
Obesity	0.832***	0.979	0.998	0.987	1.112***	1.067***	1.088***
	(0.782 - 0.885)	(0.941 - 1.019)	(0.975 - 1.020)	(0.957 - 1.019)	(1.100 - 1.123)	(1.054 - 1.080)	(1.069 - 1.106)
	0.000	0.300	0.832	0.426	0.000	0.000	0.000
Paralysis	1.210**	1.052	1.133***	0.848***	1.018	0.863***	1.037
	(1.035 - 1.416)	(0.916 - 1.209)	(1.056 - 1.216)	(0.757 - 0.949)	(0.978 - 1.060)	(0.824 - 0.904)	(0.979 - 1.099)
	0.017	0.470	0.001	0.004	0.383	0.000	0.213
Peripheral vascular disease	1.144***	0.953	1.122***	0.904***	1.106***	0.996	1.127***

© 2024 Reger MA et al. *JAMA Network Open.*

	(1.072 - 1.221)	(0.886 - 1.026)	(1.086 - 1.158)	(0.856 - 0.956)	(1.088 - 1.124)	(0.976 - 1.017)	(1.098 - 1.157)
	0.000	0.203	0.000	0.000	0.000	0.707	0.000
Psychoses	1.001	1.189***	1.218***	1.544***	1.070***	1.186***	1.139***
	(0.942 - 1.063)	(1.147 - 1.232)	(1.192 - 1.244)	(1.500 - 1.590)	(1.059 - 1.081)	(1.173 - 1.200)	(1.121 - 1.158)
	0.982	0.000	0.000	0.000	0.000	0.000	0.000
Pulmonary circulation disease	1.205***	1.082	1.137***	1.002	1.066***	1.021	1.127***
	(1.080 - 1.345)	(0.955 - 1.226)	(1.074 - 1.203)	(0.910 - 1.103)	(1.032 - 1.101)	(0.983 - 1.061)	(1.074 - 1.183)
	0.001	0.217	0.000	0.975	0.000	0.287	0.000
Renal (kidney) failure and disease, moderate	1.053	0.933	1.030	0.992	1.030***	1.002	1.009
	(0.971 - 1.142)	(0.850 - 1.024)	(0.990 - 1.072)	(0.928 - 1.061)	(1.010 - 1.051)	(0.976 - 1.028)	(0.977 - 1.043)
	0.211	0.142	0.148	0.818	0.003	0.896	0.578
Renal (kidney) failure and disease, severe	1.978***	0.669***	1.246***	0.641***	1.053**	0.897***	1.069**
	(1.764 - 2.218)	(0.539 - 0.831)	(1.166 - 1.330)	(0.545 - 0.753)	(1.012 - 1.097)	(0.852 - 0.944)	(1.010 - 1.131)
	0.000	0.000	0.000	0.000	0.012	0.000	0.022
Renal failure	0.826***	1.048	0.984	1.016	1.033*	1.017	1.037
	(0.717 - 0.951)	(0.904 - 1.213)	(0.923 - 1.050)	(0.913 - 1.131)	(0.997 - 1.070)	(0.974 - 1.061)	(0.985 - 1.093)
	0.008	0.535	0.626	0.766	0.071	0.439	0.166
Hypothyroidism	1.003	1.058*	1.044**	1.068***	1.053***	1.034***	1.015
	(0.930 - 1.082)	(0.993 - 1.127)	(1.009 - 1.080)	(1.016 - 1.122)	(1.036 - 1.070)	(1.014 - 1.055)	(0.988 - 1.043)
	0.931	0.081	0.013	0.010	0.000	0.001	0.271
Other thyroid disorders	0.833**	0.919	1.018	0.898**	1.074***	1.016	1.126***
	(0.710 - 0.979)	(0.811 - 1.041)	(0.958 - 1.082)	(0.817 - 0.987)	(1.042 - 1.107)	(0.981 - 1.053)	(1.075 - 1.179)
	0.026	0.184	0.561	0.026	0.000	0.378	0.000
Peptic ulcer with bleeding	1.054	1.298***	1.093**	1.092*	1.020	1.017	1.130***
	(0.904 - 1.228)	(1.143 - 1.473)	(1.019 - 1.172)	(0.988 - 1.207)	(0.980 - 1.062)	(0.972 - 1.064)	(1.066 - 1.198)
	0.501	0.000	0.012	0.085	0.325	0.471	0.000
Valvular disease	1.118**	0.961	1.073***	0.961	1.052***	0.969*	1.066***
	(1.022 - 1.224)	(0.858 - 1.076)	(1.023 - 1.125)	(0.883 - 1.045)	(1.024 - 1.080)	(0.938 - 1.001)	(1.025 - 1.110)
	0.015	0.490	0.004	0.350	0.000	0.056	0.002
Weight loss	1.385***	1.076*	1.119***	1.034	1.048***	0.996	1.081***
	(1.281 - 1.497)	(0.998 - 1.160)	(1.079 - 1.161)	(0.978 - 1.092)	(1.026 - 1.069)	(0.972 - 1.021)	(1.048 - 1.116)
	0.000	0.055	0.000	0.236	0.000	0.752	0.000
Past-year inpatient mental health use	0.878***	1.319***	1.642***	2.306***	1.051***	1.136***	1.383***
	(0.796 - 0.969)	(1.257 - 1.384)	(1.596 - 1.690)	(2.224 - 2.390)	(1.033 - 1.070)	(1.115 - 1.157)	(1.350 - 1.417)
	0.010	0.000	0.000	0.000	0.000	0.000	0.000
Past-year outpatient mental health use	0.849***	1.221***	1.198***	1.415***	1.436***	2.069***	1.268***
	(0.799 - 0.901)	(1.166 - 1.278)	(1.167 - 1.229)	(1.358 - 1.475)	(1.420 - 1.451)	(2.041 - 2.098)	(1.245 - 1.292)
	0.000	0.000	0.000	0.000	0.000	0.000	0.000
Past-year suicide attempt	1.021	4.786***	1.502***	1.700***	1.265***	1.393***	1.199***
	(0.912 - 1.143)	(4.593 - 4.987)	(1.455 - 1.551)	(1.637 - 1.766)	(1.242 - 1.288)	(1.366 - 1.420)	(1.168 - 1.231)
	0.722	0.000	0.000	0.000	0.000	0.000	0.000
2019	0.970	1.064**	0.884***	0.896***	0.988	0.929***	0.875***
	(0.895 - 1.050)	(1.010 - 1.120)	(0.858 - 0.910)	(0.861 - 0.933)	(0.974 - 1.003)	(0.914 - 0.944)	(0.855 - 0.895)
	0.448	0.020	0.000	0.000	0.121	0.000	0.000
2020	1.136	1.171***	0.780***	0.776***	0.955***	0.822***	0.748***
	(0.974 - 1.325)	(1.061 - 1.293)	(0.736 - 0.827)	(0.716 - 0.841)	(0.928 - 0.982)	(0.796 - 0.850)	(0.715 - 0.781)
	0.105	0.002	0.000	0.000	0.001	0.000	0.000

© 2024 Reger MA et al. *JAMA Network Open.*

2021	1.184	1.197**	0.772***	0.735***	1.112***	0.923***	0.734***
	(0.952 - 1.473)	(1.039 - 1.379)	(0.710 - 0.840)	(0.655 - 0.825)	(1.069 - 1.157)	(0.881 - 0.966)	(0.689 - 0.781)
	0.129	0.013	0.000	0.000	0.000	0.001	0.000
February	1.134**	1.048	0.991	0.965	1.024**	1.015	1.012
	(1.009 - 1.276)	(0.969 - 1.132)	(0.947 - 1.038)	(0.906 - 1.029)	(1.002 - 1.046)	(0.990 - 1.041)	(0.979 - 1.047)
	0.035	0.241	0.714	0.274	0.031	0.235	0.477
March	1.157**	1.025	1.005	0.977	1.030**	1.078***	0.972
	(1.020 - 1.313)	(0.940 - 1.118)	(0.956 - 1.055)	(0.911 - 1.047)	(1.007 - 1.053)	(1.050 - 1.107)	(0.937 - 1.008)
	0.023	0.575	0.858	0.502	0.011	0.000	0.126
April	1.180**	1.080*	1.019	0.988	1.014	1.050***	1.007
	(1.037 - 1.342)	(0.990 - 1.177)	(0.970 - 1.071)	(0.922 - 1.059)	(0.991 - 1.037)	(1.023 - 1.079)	(0.970 - 1.044)
	0.012	0.083	0.454	0.741	0.240	0.000	0.728
May	1.142**	1.084*	1.051**	1.008	1.017	1.090***	1.036*
	(1.004 - 1.299)	(0.995 - 1.182)	(1.001 - 1.104)	(0.941 - 1.080)	(0.995 - 1.040)	(1.062 - 1.119)	(0.999 - 1.074)
	0.044	0.064	0.048	0.812	0.134	0.000	0.059
June	1.239***	1.039	0.951**	0.908***	1.003	1.011	0.944***
	(1.089 - 1.409)	(0.954 - 1.132)	(0.904 - 0.999)	(0.847 - 0.973)	(0.981 - 1.027)	(0.984 - 1.038)	(0.909 - 0.979)
	0.001	0.383	0.045	0.006	0.770	0.430	0.002
July	1.185**	1.051	0.926***	0.886***	1.006	0.977	0.931***
	(1.034 - 1.357)	(0.962 - 1.149)	(0.879 - 0.975)	(0.825 - 0.951)	(0.981 - 1.031)	(0.949 - 1.005)	(0.896 - 0.968)
	0.015	0.272	0.003	0.001	0.646	0.101	0.000
August	1.094	1.061	0.916***	0.915**	0.996	0.967**	0.934***
	(0.954 - 1.255)	(0.971 - 1.160)	(0.870 - 0.965)	(0.852 - 0.982)	(0.972 - 1.021)	(0.940 - 0.995)	(0.899 - 0.971)
	0.200	0.190	0.001	0.013	0.763	0.020	0.001
September	1.156**	1.065	0.923***	0.894***	1.021*	0.979	0.914***
	(1.008 - 1.327)	(0.973 - 1.165)	(0.876 - 0.972)	(0.832 - 0.961)	(0.996 - 1.047)	(0.951 - 1.007)	(0.879 - 0.951)
	0.039	0.173	0.003	0.002	0.099	0.137	0.000
October	1.236***	1.071	0.895***	0.866***	1.009	0.972*	0.892***
	(1.079 - 1.415)	(0.979 - 1.172)	(0.850 - 0.943)	(0.806 - 0.930)	(0.984 - 1.034)	(0.945 - 1.000)	(0.858 - 0.927)
	0.002	0.133	0.000	0.000	0.492	0.052	0.000
November	1.241***	1.015	0.899***	0.862***	1.000	0.958***	0.908***
	(1.081 - 1.425)	(0.926 - 1.113)	(0.853 - 0.949)	(0.801 - 0.928)	(0.975 - 1.025)	(0.931 - 0.986)	(0.873 - 0.945)
	0.002	0.747	0.000	0.000	0.970	0.004	0.000
December	1.151**	1.081*	0.894***	0.822***	0.976*	0.930***	0.909***
	(1.000 - 1.324)	(0.987 - 1.184)	(0.847 - 0.943)	(0.764 - 0.885)	(0.952 - 1.001)	(0.904 - 0.957)	(0.874 - 0.946)
	0.050	0.094	0.000	0.000	0.056	0.000	0.000
Observations	217,747	217,747	217,747	217,747	217,747	217,747	217,747
AIC	174177	390983	1.144e+06	586494	4.757e+06	3.731e+06	2.060e+06
BIC	176914	393721	1.147e+06	589232	4.760e+06	3.734e+06	2.063e+06

Notes: *** p<0.01, ** p<0.05, * p<0.1; Coefficients are hazard ratios with 95% CI in parentheses, followed by p-value; Standard errors were clustered on patienticn level to account for multiple observations per individual; Excluded those with age at separation missing due to small cell size.

eTable 11. Hazard Ratios from Time-to-Event Analyses of Association between Outcomes and Caring Letters Receipt, Repeat Callers

VARIABLES	All-Cause Mortality	Suicide Attempt	Inpatient	Inpatient Mental Health	Outpatient	Outpatient Mental Health Care	Emergency Department Use
**Caring Letters Received**	0.898	1.144*	**1.133*** **	**1.149** **	**1.059*** **	**1.132*** **	**1.103*** **

© 2024 Reger MA et al. *JAMA Network Open.*

	(0.725 - 1.112)	(0.997 - 1.313)	**(1.042 - 1.233)**	**(1.030 - 1.281)**	**(1.015 - 1.106)**	**(1.078 - 1.188)**	**(1.032 - 1.179)**
	0.322	0.056	**0.004**	**0.013**	**0.009**	**0.000**	**0.004**
Call during COVID Period	0.959	0.820**	0.885**	0.900	0.814***	0.712***	1.058
	(0.717 - 1.283)	(0.683 - 0.983)	(0.792 - 0.989)	(0.778 - 1.041)	(0.767 - 0.864)	(0.666 - 0.761)	(0.968 - 1.157)
	0.777	0.032	0.031	0.157	0.000	0.000	0.215
Age 40-54	1.776***	0.835***	1.047*	0.957	1.015	0.973*	0.987
	(1.444 - 2.183)	(0.771 - 0.903)	(0.993 - 1.104)	(0.896 - 1.021)	(0.987 - 1.043)	(0.945 - 1.002)	(0.947 - 1.028)
	0.000	0.000	0.091	0.181	0.294	0.068	0.523
Age 55-64	2.451***	0.713***	1.048*	0.922**	1.000	0.891***	0.957**
	(2.013 - 2.986)	(0.653 - 0.777)	(0.991 - 1.107)	(0.861 - 0.988)	(0.972 - 1.029)	(0.864 - 0.920)	(0.916 - 0.999)
	0.000	0.000	0.099	0.022	0.999	0.000	0.044
Age 65-79	3.555***	0.519***	0.988	0.773***	0.962**	0.748***	0.902***
	(2.914 - 4.337)	(0.463 - 0.581)	(0.928 - 1.053)	(0.710 - 0.842)	(0.932 - 0.994)	(0.721 - 0.776)	(0.858 - 0.948)
	0.000	0.000	0.719	0.000	0.021	0.000	0.000
Age over 80	7.513***	0.215***	0.976	0.341***	1.021	0.469***	0.827***
	(5.886 - 9.589)	(0.145 - 0.317)	(0.861 - 1.106)	(0.258 - 0.451)	(0.963 - 1.083)	(0.431 - 0.509)	(0.750 - 0.913)
	0.000	0.000	0.704	0.000	0.484	0.000	0.000
Female	0.559***	0.999	0.834***	0.863***	1.085***	1.089***	1.013
	(0.461 - 0.678)	(0.922 - 1.083)	(0.791 - 0.880)	(0.806 - 0.924)	(1.058 - 1.114)	(1.059 - 1.120)	(0.974 - 1.053)
	0.000	0.985	0.000	0.000	0.000	0.000	0.522
American Indian	0.903	1.137	1.068	0.997	0.950	0.928*	0.921
	(0.581 - 1.404)	(0.916 - 1.411)	(0.922 - 1.237)	(0.815 - 1.219)	(0.874 - 1.032)	(0.849 - 1.014)	(0.811 - 1.045)
	0.651	0.245	0.380	0.977	0.222	0.100	0.200
Asian	0.441*	0.907	0.850	1.002	0.985	0.988	0.811***
	(0.180 - 1.079)	(0.687 - 1.200)	(0.694 - 1.039)	(0.792 - 1.267)	(0.901 - 1.078)	(0.890 - 1.098)	(0.697 - 0.944)
	0.073	0.495	0.113	0.988	0.743	0.826	0.007
Black	0.548***	0.856***	0.967	1.016	1.011	1.020	1.122***
	(0.480 - 0.626)	(0.792 - 0.925)	(0.926 - 1.011)	(0.960 - 1.074)	(0.988 - 1.036)	(0.994 - 1.047)	(1.084 - 1.162)
	0.000	0.000	0.138	0.592	0.342	0.141	0.000
More than one race	0.745	0.832	0.833**	0.823*	1.009	1.051	1.035
	(0.469 - 1.183)	(0.652 - 1.063)	(0.710 - 0.977)	(0.672 - 1.008)	(0.934 - 1.089)	(0.963 - 1.148)	(0.920 - 1.164)
	0.213	0.141	0.025	0.059	0.829	0.265	0.566
Native Hawaiian	0.817	1.044	1.012	1.009	1.009	0.975	0.949
	(0.471 - 1.418)	(0.776 - 1.403)	(0.823 - 1.244)	(0.781 - 1.303)	(0.918 - 1.109)	(0.876 - 1.085)	(0.813 - 1.108)
	0.473	0.778	0.913	0.944	0.858	0.640	0.510
Unknown race	0.770**	0.942	0.921*	0.970	0.929***	0.932***	0.976
	(0.623 - 0.951)	(0.830 - 1.070)	(0.848 - 1.000)	(0.873 - 1.077)	(0.892 - 0.969)	(0.891 - 0.975)	(0.916 - 1.039)
	0.015	0.361	0.051	0.568	0.001	0.002	0.443
Non-Hispanic	1.137	0.926	0.995	0.968	1.003	0.993	0.931**
	(0.911 - 1.418)	(0.832 - 1.032)	(0.925 - 1.071)	(0.882 - 1.061)	(0.966 - 1.041)	(0.953 - 1.035)	(0.880 - 0.985)
	0.257	0.164	0.899	0.485	0.889	0.731	0.013
Unknown Ethnicity	1.058	0.990	0.955	0.981	0.863***	0.943*	0.839***
	(0.757 - 1.478)	(0.825 - 1.188)	(0.845 - 1.079)	(0.842 - 1.144)	(0.812 - 0.917)	(0.882 - 1.007)	(0.764 - 0.922)
	0.741	0.915	0.457	0.811	0.000	0.081	0.000
Never Married	1.116	0.945	1.149***	1.164***	0.991	0.994	1.106***
	(0.968 - 1.287)	(0.873 - 1.023)	(1.094 - 1.207)	(1.092 - 1.241)	(0.966 - 1.017)	(0.966 - 1.022)	(1.065 - 1.149)
	0.131	0.162	0.000	0.000	0.494	0.673	0.000

© 2024 Reger MA et al. *JAMA Network Open.*

Divorced/Separated/Widowed	1.253***	1.010	1.143***	1.143***	0.997	0.993	1.096***
	(1.119 - 1.402)	(0.939 - 1.085)	(1.094 - 1.195)	(1.078 - 1.212)	(0.975 - 1.019)	(0.969 - 1.019)	(1.059 - 1.134)
	0.000	0.794	0.000	0.000	0.761	0.611	0.000
Marital Status Missing	1.467	0.837	0.651***	0.604***	0.732***	0.859***	0.608***
	(0.892 - 2.413)	(0.607 - 1.155)	(0.498 - 0.850)	(0.421 - 0.865)	(0.659 - 0.813)	(0.767 - 0.963)	(0.499 - 0.740)
	0.131	0.279	0.002	0.006	0.000	0.009	0.000
Army	0.951	0.934	1.033	1.002	0.980	0.959**	1.044*
	(0.828 - 1.091)	(0.851 - 1.026)	(0.978 - 1.092)	(0.932 - 1.077)	(0.953 - 1.009)	(0.929 - 0.990)	(1.000 - 1.091)
	0.472	0.153	0.241	0.958	0.174	0.011	0.052
Navy	0.941	1.022	1.030	1.000	0.991	0.989	1.039
	(0.804 - 1.101)	(0.920 - 1.134)	(0.968 - 1.096)	(0.921 - 1.086)	(0.959 - 1.025)	(0.953 - 1.026)	(0.989 - 1.091)
	0.448	0.689	0.353	0.996	0.611	0.542	0.132
Marine Corps	0.917	1.002	1.024	0.978	0.988	0.989	0.988
	(0.766 - 1.097)	(0.893 - 1.125)	(0.955 - 1.098)	(0.892 - 1.072)	(0.952 - 1.024)	(0.950 - 1.030)	(0.934 - 1.045)
	0.342	0.969	0.504	0.628	0.503	0.596	0.671
Others and missing	1.153	0.957	1.132	0.957	1.001	0.977	1.078
	(0.794 - 1.674)	(0.726 - 1.261)	(0.968 - 1.323)	(0.768 - 1.192)	(0.917 - 1.094)	(0.887 - 1.077)	(0.947 - 1.227)
	0.454	0.754	0.121	0.696	0.976	0.641	0.256
Age at separation (Q2)	0.914	0.928*	1.021	1.071**	1.015	1.006	0.979
	(0.810 - 1.031)	(0.856 - 1.006)	(0.974 - 1.071)	(1.007 - 1.139)	(0.989 - 1.041)	(0.977 - 1.035)	(0.942 - 1.018)
	0.143	0.071	0.381	0.030	0.263	0.712	0.290
Age at separation (Q3)	0.930	0.902**	1.032	1.016	0.993	0.986	0.985
	(0.817 - 1.058)	(0.831 - 0.979)	(0.983 - 1.083)	(0.954 - 1.083)	(0.967 - 1.020)	(0.957 - 1.016)	(0.947 - 1.024)
	0.268	0.013	0.212	0.618	0.608	0.350	0.437
Age at separation (Q4)	0.858**	0.868***	0.942**	0.970	1.007	1.007	0.941***
	(0.747 - 0.986)	(0.797 - 0.946)	(0.894 - 0.993)	(0.906 - 1.038)	(0.980 - 1.034)	(0.977 - 1.038)	(0.903 - 0.980)
	0.030	0.001	0.026	0.374	0.620	0.656	0.003
Dishonorable	0.956	1.123	1.012	1.107	0.870**	0.918	1.176*
	(0.539 - 1.697)	(0.822 - 1.536)	(0.813 - 1.260)	(0.852 - 1.440)	(0.781 - 0.971)	(0.809 - 1.041)	(0.992 - 1.394)
	0.879	0.465	0.913	0.446	0.013	0.183	0.062
Other discharge	0.845	1.159***	1.072**	1.132***	0.948***	0.976	1.053**
	(0.686 - 1.040)	(1.056 - 1.271)	(1.009 - 1.139)	(1.053 - 1.217)	(0.916 - 0.981)	(0.941 - 1.012)	(1.002 - 1.106)
	0.111	0.002	0.025	0.001	0.002	0.184	0.040
Unknown discharge	0.716	0.955	0.937	0.931	0.991	0.947	0.944
	(0.420 - 1.220)	(0.710 - 1.284)	(0.775 - 1.132)	(0.728 - 1.192)	(0.902 - 1.087)	(0.851 - 1.054)	(0.821 - 1.086)
	0.219	0.760	0.497	0.573	0.843	0.317	0.419
Acquired immune deficiency	0.813	1.353***	1.168**	1.195**	1.200***	0.983	1.288***
syndrome	(0.521 - 1.268)	(1.095 - 1.672)	(1.026 - 1.330)	(1.013 - 1.410)	(1.107 - 1.301)	(0.893 - 1.082)	(1.162 - 1.429)
	0.361	0.005	0.019	0.035	0.000	0.721	0.000
Alcohol disorder	1.286***	1.229***	1.434***	1.264***	1.016	1.039***	1.080***
	(1.147 - 1.443)	(1.148 - 1.317)	(1.375 - 1.495)	(1.196 - 1.335)	(0.993 - 1.039)	(1.014 - 1.065)	(1.045 - 1.117)
	0.000	0.000	0.000	0.000	0.178	0.002	0.000
Anemias due to other	1.192***	1.163***	1.206***	1.089**	1.026	1.008	1.144***
nutritional deficiencies	(1.049 - 1.353)	(1.057 - 1.278)	(1.146 - 1.269)	(1.017 - 1.166)	(0.994 - 1.059)	(0.973 - 1.044)	(1.095 - 1.195)
	0.007	0.002	0.000	0.014	0.108	0.662	0.000
Autoimmune conditions	0.874	0.987	1.122**	0.972	1.157***	1.031	1.140***
	(0.659 - 1.159)	(0.820 - 1.188)	(1.017 - 1.238)	(0.844 - 1.119)	(1.095 - 1.221)	(0.966 - 1.100)	(1.050 - 1.237)

© 2024 Reger MA et al. *JAMA Network Open.*

	0.350	0.888	0.021	0.692	0.000	0.355	0.002
Chronic blood loss (iron deficiency)	1.328*	1.201	0.940	0.992	0.977	0.990	0.952
	(0.979 - 1.802)	(0.909 - 1.586)	(0.811 - 1.089)	(0.795 - 1.239)	(0.890 - 1.073)	(0.885 - 1.107)	(0.834 - 1.086)
	0.068	0.198	0.409	0.946	0.628	0.857	0.461
Leukemia	1.746**	1.034	1.088	0.666	1.018	1.016	0.966
	(1.068 - 2.855)	(0.496 - 2.157)	(0.761 - 1.556)	(0.349 - 1.274)	(0.835 - 1.241)	(0.811 - 1.272)	(0.717 - 1.302)
	0.026	0.929	0.645	0.219	0.858	0.892	0.821
Lymphoma	0.569**	0.445**	0.952	0.770	1.002	0.881	1.132
	(0.328 - 0.987)	(0.210 - 0.942)	(0.761 - 1.191)	(0.519 - 1.142)	(0.873 - 1.151)	(0.746 - 1.040)	(0.951 - 1.348)
	0.045	0.034	0.668	0.193	0.975	0.134	0.164
Metastatic cancer	2.445***	0.704	1.325***	0.667*	1.108	0.834**	1.222**
	(1.779 - 3.361)	(0.389 - 1.273)	(1.074 - 1.634)	(0.441 - 1.008)	(0.967 - 1.271)	(0.704 - 0.989)	(1.025 - 1.456)
	0.000	0.246	0.008	0.055	0.141	0.037	0.025
Solid tumor without metastasis, in situ	1.022	0.994	0.998	1.079	1.070**	0.962	1.032
	(0.807 - 1.293)	(0.761 - 1.298)	(0.877 - 1.135)	(0.896 - 1.299)	(1.003 - 1.142)	(0.887 - 1.043)	(0.928 - 1.148)
	0.859	0.965	0.971	0.422	0.041	0.349	0.558
Solid tumor without metastasis, malignant	1.398***	0.984	1.082*	0.962	1.089***	0.970	1.113***
	(1.185 - 1.649)	(0.817 - 1.186)	(0.993 - 1.179)	(0.848 - 1.090)	(1.039 - 1.140)	(0.914 - 1.028)	(1.035 - 1.197)
	0.000	0.869	0.073	0.542	0.000	0.304	0.004
Cerebrovascular disease - Primary	1.210***	1.021	1.192***	1.109***	1.067***	1.008	1.135***
	(1.077 - 1.359)	(0.942 - 1.107)	(1.139 - 1.247)	(1.045 - 1.177)	(1.039 - 1.095)	(0.979 - 1.038)	(1.093 - 1.179)
	0.001	0.613	0.000	0.001	0.000	0.606	0.000
Cerebrovascular disease - Sequela	0.889	0.845	0.975	1.086	0.997	1.045	1.030
	(0.661 - 1.196)	(0.653 - 1.094)	(0.856 - 1.111)	(0.900 - 1.312)	(0.919 - 1.081)	(0.955 - 1.145)	(0.921 - 1.151)
	0.437	0.201	0.704	0.390	0.936	0.338	0.603
Coagulopathy	1.256**	0.843**	1.197***	1.010	1.107***	1.020	1.144***
	(1.054 - 1.497)	(0.722 - 0.984)	(1.109 - 1.292)	(0.908 - 1.123)	(1.051 - 1.166)	(0.963 - 1.079)	(1.066 - 1.228)
	0.011	0.031	0.000	0.853	0.000	0.503	0.000
Dementia	1.065	0.960	1.042	1.063	0.912***	0.929**	1.034
	(0.893 - 1.270)	(0.804 - 1.145)	(0.958 - 1.133)	(0.945 - 1.196)	(0.866 - 0.960)	(0.876 - 0.986)	(0.960 - 1.113)
	0.483	0.650	0.340	0.308	0.000	0.015	0.379
Depression	0.930	1.098**	0.982	0.921***	1.085***	1.112***	1.034**
	(0.833 - 1.038)	(1.020 - 1.183)	(0.940 - 1.026)	(0.869 - 0.976)	(1.062 - 1.109)	(1.085 - 1.139)	(1.000 - 1.069)
	0.194	0.013	0.418	0.005	0.000	0.000	0.048
Diabetes with chronic complications	1.329***	0.977	1.059*	0.905**	1.105***	0.964*	1.132***
	(1.140 - 1.551)	(0.862 - 1.107)	(0.991 - 1.132)	(0.825 - 0.993)	(1.066 - 1.144)	(0.925 - 1.004)	(1.073 - 1.194)
	0.000	0.717	0.089	0.035	0.000	0.077	0.000
Diabetes without chronic complications	0.987	1.009	1.011	1.085**	1.009	1.033*	0.989
	(0.851 - 1.144)	(0.906 - 1.124)	(0.952 - 1.074)	(1.000 - 1.177)	(0.978 - 1.042)	(0.996 - 1.072)	(0.941 - 1.038)
	0.858	0.867	0.714	0.049	0.568	0.080	0.643
Drug use disorder	0.976	1.102***	1.283***	1.324***	1.054***	1.073***	1.151***
	(0.864 - 1.103)	(1.027 - 1.184)	(1.229 - 1.339)	(1.252 - 1.401)	(1.029 - 1.080)	(1.046 - 1.102)	(1.112 - 1.192)
	0.700	0.007	0.000	0.000	0.000	0.000	0.000
Fluid and electrolyte disorders	1.296***	1.070	1.376***	1.149***	1.034**	1.002	1.313***
	(1.145 - 1.468)	(0.984 - 1.164)	(1.314 - 1.441)	(1.081 - 1.221)	(1.005 - 1.064)	(0.970 - 1.034)	(1.261 - 1.366)
	0.000	0.114	0.000	0.000	0.022	0.924	0.000
Heart failure	1.511***	1.035	1.153***	0.902*	1.076***	0.954*	1.069**

© 2024 Reger MA et al. *JAMA Network Open.*

	(1.293 - 1.766)	(0.890 - 1.204)	(1.073 - 1.240)	(0.808 - 1.006)	(1.030 - 1.124)	(0.905 - 1.006)	(1.003 - 1.140)
	0.000	0.655	0.000	0.064	0.001	0.085	0.039
Homeless	0.928	0.957	1.179***	1.221***	0.983	1.046***	1.180***
	(0.824 - 1.045)	(0.895 - 1.023)	(1.133 - 1.228)	(1.161 - 1.285)	(0.960 - 1.006)	(1.019 - 1.073)	(1.141 - 1.219)
	0.219	0.199	0.000	0.000	0.146	0.001	0.000
Hypertension, complicated	1.176**	1.093	1.124***	1.053	0.973	0.954**	1.079**
	(1.006 - 1.376)	(0.958 - 1.247)	(1.052 - 1.201)	(0.959 - 1.155)	(0.935 - 1.013)	(0.911 - 1.000)	(1.018 - 1.144)
	0.042	0.188	0.001	0.278	0.178	0.049	0.010
Hypertension, uncomplicated	0.970	0.985	1.113***	1.060**	1.068***	0.970**	1.048***
	(0.869 - 1.082)	(0.918 - 1.058)	(1.068 - 1.161)	(1.004 - 1.119)	(1.044 - 1.091)	(0.946 - 0.994)	(1.014 - 1.084)
	0.580	0.684	0.000	0.037	0.000	0.015	0.006
Liver disease, mild	1.362***	0.994	1.203***	1.073**	1.037**	1.002	1.175***
	(1.191 - 1.556)	(0.906 - 1.091)	(1.145 - 1.264)	(1.006 - 1.145)	(1.006 - 1.069)	(0.969 - 1.036)	(1.125 - 1.227)
	0.000	0.906	0.000	0.033	0.020	0.917	0.000
Liver disease and failure,	1.395**	0.997	1.026	0.878	0.928	0.935	0.987
moderate to severe	(1.080 - 1.803)	(0.770 - 1.290)	(0.906 - 1.161)	(0.728 - 1.060)	(0.846 - 1.017)	(0.845 - 1.035)	(0.878 - 1.109)
	0.011	0.980	0.689	0.176	0.110	0.194	0.822
Liver disease	2.199***	1.473	1.282*	0.996	1.119	1.057	1.048
	(1.285 - 3.763)	(0.924 - 2.350)	(0.980 - 1.676)	(0.670 - 1.480)	(0.931 - 1.345)	(0.857 - 1.303)	(0.816 - 1.347)
	0.004	0.104	0.070	0.983	0.233	0.606	0.712
Chronic pulmonary disease	1.223***	1.081**	1.087***	1.015	1.091***	1.035**	1.107***
	(1.100 - 1.360)	(1.001 - 1.168)	(1.041 - 1.134)	(0.959 - 1.074)	(1.065 - 1.118)	(1.006 - 1.064)	(1.069 - 1.148)
	0.000	0.048	0.000	0.602	0.000	0.016	0.000
Neurological disorders	0.819*	1.021	1.050	1.198***	1.046*	1.101***	1.103***
affecting movement	(0.654 - 1.026)	(0.878 - 1.187)	(0.962 - 1.146)	(1.075 - 1.334)	(0.996 - 1.099)	(1.042 - 1.164)	(1.027 - 1.186)
	0.083	0.786	0.272	0.001	0.075	0.001	0.008
Other neurological disorders	1.068	1.062	0.995	1.039	1.085***	1.034	1.019
	(0.925 - 1.232)	(0.953 - 1.185)	(0.936 - 1.058)	(0.959 - 1.127)	(1.046 - 1.125)	(0.992 - 1.078)	(0.966 - 1.074)
	0.372	0.278	0.869	0.348	0.000	0.114	0.490
Seizures and epilepsy	1.111	1.048	1.080**	0.969	1.054**	1.007	1.079***
	(0.930 - 1.327)	(0.934 - 1.176)	(1.012 - 1.153)	(0.891 - 1.055)	(1.012 - 1.098)	(0.964 - 1.052)	(1.019 - 1.144)
	0.248	0.423	0.021	0.473	0.011	0.760	0.010
Obesity	0.872**	1.081**	1.018	0.981	1.115***	1.094***	1.088***
	(0.774 - 0.982)	(1.006 - 1.162)	(0.976 - 1.063)	(0.928 - 1.037)	(1.089 - 1.142)	(1.066 - 1.124)	(1.051 - 1.126)
	0.024	0.035	0.401	0.503	0.000	0.000	0.000
Paralysis	1.188	1.222*	1.071	0.772***	1.006	0.870***	0.988
	(0.880 - 1.605)	(0.965 - 1.549)	(0.938 - 1.223)	(0.635 - 0.939)	(0.924 - 1.096)	(0.794 - 0.954)	(0.882 - 1.106)
	0.260	0.097	0.309	0.010	0.890	0.003	0.833
Peripheral vascular disease	1.123*	1.043	1.101***	0.970	1.065***	1.014	1.089***
	(0.979 - 1.288)	(0.912 - 1.192)	(1.032 - 1.175)	(0.880 - 1.069)	(1.025 - 1.105)	(0.969 - 1.061)	(1.031 - 1.150)
	0.099	0.539	0.004	0.537	0.001	0.549	0.002
Psychoses	0.999	1.084**	1.165***	1.391***	1.063***	1.159***	1.114***
	(0.898 - 1.112)	(1.018 - 1.156)	(1.122 - 1.211)	(1.323 - 1.463)	(1.041 - 1.085)	(1.133 - 1.186)	(1.081 - 1.149)
	0.988	0.013	0.000	0.000	0.000	0.000	0.000
Pulmonary circulation disease	1.395***	0.928	1.159***	0.835**	1.032	1.019	1.051
	(1.137 - 1.713)	(0.742 - 1.161)	(1.040 - 1.291)	(0.702 - 0.994)	(0.961 - 1.108)	(0.940 - 1.105)	(0.953 - 1.159)
	0.001	0.513	0.007	0.042	0.382	0.645	0.317

© 2024 Reger MA et al. *JAMA Network Open.*

Renal (kidney) failure and disease, moderate	1.096	0.869	1.036	0.962	1.010	1.027	1.036
	(0.928 - 1.293)	(0.732 - 1.031)	(0.957 - 1.121)	(0.857 - 1.079)	(0.964 - 1.059)	(0.973 - 1.085)	(0.968 - 1.110)
	0.281	0.106	0.382	0.504	0.674	0.336	0.309
Renal (kidney) failure and disease, severe	1.970***	0.777	1.292***	0.676**	1.067	0.961	1.079
	(1.537 - 2.526)	(0.525 - 1.149)	(1.127 - 1.480)	(0.496 - 0.919)	(0.966 - 1.179)	(0.856 - 1.078)	(0.952 - 1.223)
	0.000	0.206	0.000	0.013	0.199	0.499	0.235
Renal failure	0.876	1.040	0.970	1.178*	1.006	0.992	1.079
	(0.655 - 1.171)	(0.799 - 1.353)	(0.849 - 1.108)	(0.977 - 1.420)	(0.925 - 1.094)	(0.900 - 1.093)	(0.963 - 1.208)
	0.372	0.773	0.652	0.087	0.895	0.868	0.189
Hypothyroidism	0.975	1.055	1.031	1.107**	1.076***	1.085***	1.006
	(0.832 - 1.144)	(0.942 - 1.182)	(0.967 - 1.100)	(1.017 - 1.204)	(1.038 - 1.115)	(1.041 - 1.131)	(0.953 - 1.062)
	0.760	0.355	0.347	0.018	0.000	0.000	0.828
Other thyroid disorders	1.099	1.051	1.013	0.797***	1.068*	1.035	1.165***
	(0.811 - 1.489)	(0.850 - 1.300)	(0.902 - 1.138)	(0.673 - 0.944)	(0.999 - 1.142)	(0.958 - 1.118)	(1.058 - 1.282)
	0.543	0.647	0.827	0.009	0.055	0.380	0.002
Peptic ulcer with bleeding	1.114	1.191	1.053	1.008	0.957	0.984	1.104*
	(0.837 - 1.482)	(0.958 - 1.480)	(0.929 - 1.195)	(0.850 - 1.196)	(0.882 - 1.038)	(0.901 - 1.076)	(0.992 - 1.229)
	0.459	0.116	0.420	0.924	0.287	0.729	0.069
Valvular disease	1.133	0.811*	1.001	0.968	1.071**	1.033	1.107**
	(0.945 - 1.359)	(0.657 - 1.000)	(0.910 - 1.101)	(0.842 - 1.112)	(1.010 - 1.136)	(0.963 - 1.107)	(1.021 - 1.202)
	0.178	0.050	0.982	0.641	0.022	0.363	0.014
Weight loss	1.138	1.071	1.063*	1.016	1.008	0.942**	1.053*
	(0.969 - 1.336)	(0.941 - 1.219)	(0.992 - 1.139)	(0.922 - 1.119)	(0.965 - 1.052)	(0.897 - 0.989)	(0.990 - 1.119)
	0.116	0.300	0.083	0.752	0.727	0.016	0.099
Past-year inpatient mental health care use	0.880*	1.473***	1.718***	2.532***	1.058***	1.162***	1.419***
	(0.763 - 1.015)	(1.365 - 1.590)	(1.643 - 1.797)	(2.390 - 2.682)	(1.029 - 1.087)	(1.129 - 1.197)	(1.366 - 1.473)
	0.079	0.000	0.000	0.000	0.000	0.000	0.000
Past-year outpatient mental health care use	0.814***	1.209***	1.230***	1.347***	1.604***	2.235***	1.367***
	(0.717 - 0.923)	(1.087 - 1.345)	(1.156 - 1.308)	(1.227 - 1.478)	(1.559 - 1.650)	(2.159 - 2.314)	(1.306 - 1.432)
	0.001	0.000	0.000	0.000	0.000	0.000	0.000
Past-year suicide attempt	0.985	4.298***	1.353***	1.502***	1.168***	1.269***	1.145***
	(0.838 - 1.157)	(4.015 - 4.600)	(1.288 - 1.420)	(1.418 - 1.592)	(1.133 - 1.203)	(1.230 - 1.309)	(1.099 - 1.194)
	0.850	0.000	0.000	0.000	0.000	0.000	0.000
2019	0.830***	1.113**	0.927***	0.926**	0.983	0.942***	0.876***
	(0.725 - 0.951)	(1.020 - 1.214)	(0.881 - 0.975)	(0.867 - 0.989)	(0.956 - 1.010)	(0.914 - 0.971)	(0.841 - 0.913)
	0.007	0.016	0.004	0.023	0.220	0.000	0.000
2020	0.896	1.199**	0.829***	0.856**	0.956	0.857***	0.740***
	(0.678 - 1.184)	(1.009 - 1.425)	(0.746 - 0.921)	(0.746 - 0.982)	(0.903 - 1.013)	(0.805 - 0.914)	(0.680 - 0.805)
	0.440	0.039	0.000	0.027	0.127	0.000	0.000
2021	0.832	1.208	0.886	0.884	1.126**	1.022	0.732***
	(0.525 - 1.318)	(0.913 - 1.599)	(0.748 - 1.051)	(0.706 - 1.107)	(1.029 - 1.232)	(0.923 - 1.132)	(0.639 - 0.838)
	0.434	0.186	0.164	0.281	0.010	0.669	0.000
February	0.961	1.154*	0.989	0.957	1.047*	1.024	1.030
	(0.762 - 1.212)	(0.999 - 1.332)	(0.905 - 1.080)	(0.853 - 1.074)	(0.998 - 1.098)	(0.971 - 1.080)	(0.960 - 1.106)
	0.739	0.051	0.806	0.454	0.061	0.390	0.407
March	1.030	0.960	1.059	1.009	1.054*	1.076**	0.958
	(0.796 - 1.332)	(0.805 - 1.146)	(0.959 - 1.169)	(0.884 - 1.151)	(1.000 - 1.112)	(1.014 - 1.142)	(0.884 - 1.037)

© 2024 Reger MA et al. *JAMA Network Open.*

	0.824	0.655	0.259	0.895	0.051	0.015	0.286
April	0.928	1.119	1.012	1.010	0.995	1.048	0.955
	(0.706 - 1.220)	(0.942 - 1.331)	(0.914 - 1.121)	(0.884 - 1.155)	(0.941 - 1.051)	(0.987 - 1.114)	(0.880 - 1.036)
	0.593	0.201	0.814	0.880	0.846	0.127	0.265
May	0.809	1.113	1.068	1.026	1.019	1.023	0.996
	(0.612 - 1.070)	(0.936 - 1.322)	(0.965 - 1.181)	(0.898 - 1.172)	(0.966 - 1.075)	(0.964 - 1.086)	(0.918 - 1.080)
	0.138	0.225	0.202	0.710	0.483	0.445	0.914
June	1.074	1.224**	0.996	1.028	0.999	0.985	0.919**
	(0.836 - 1.380)	(1.043 - 1.437)	(0.906 - 1.095)	(0.907 - 1.164)	(0.950 - 1.052)	(0.930 - 1.043)	(0.851 - 0.992)
	0.578	0.013	0.934	0.668	0.982	0.600	0.031
July	0.871	0.972	0.908**	0.917	1.009	0.966	0.875***
	(0.671 - 1.129)	(0.823 - 1.147)	(0.824 - 1.000)	(0.808 - 1.040)	(0.958 - 1.062)	(0.912 - 1.023)	(0.810 - 0.945)
	0.297	0.734	0.049	0.177	0.740	0.231	0.001
August	0.854	1.005	0.921*	0.946	0.981	0.952*	0.888***
	(0.657 - 1.111)	(0.851 - 1.187)	(0.836 - 1.016)	(0.833 - 1.074)	(0.932 - 1.033)	(0.899 - 1.008)	(0.821 - 0.959)
	0.240	0.952	0.099	0.391	0.462	0.092	0.003
September	0.907	0.993	0.968	0.942	1.024	1.003	0.925**
	(0.697 - 1.180)	(0.839 - 1.175)	(0.878 - 1.067)	(0.828 - 1.072)	(0.971 - 1.079)	(0.946 - 1.063)	(0.856 - 1.000)
	0.468	0.932	0.511	0.367	0.380	0.930	0.049
October	0.903	1.198**	0.949	0.961	0.992	0.980	0.881***
	(0.693 - 1.176)	(1.016 - 1.413)	(0.860 - 1.046)	(0.845 - 1.092)	(0.941 - 1.046)	(0.924 - 1.039)	(0.815 - 0.953)
	0.449	0.032	0.293	0.539	0.769	0.497	0.002
November	0.944	1.025	0.892**	0.932	0.982	0.937**	0.851***
	(0.721 - 1.236)	(0.864 - 1.216)	(0.806 - 0.988)	(0.817 - 1.064)	(0.931 - 1.036)	(0.883 - 0.994)	(0.785 - 0.923)
	0.676	0.778	0.028	0.296	0.503	0.031	0.000
December	0.879	0.956	0.909*	0.865**	0.937**	0.896***	0.847***
	(0.670 - 1.153)	(0.805 - 1.135)	(0.822 - 1.005)	(0.758 - 0.987)	(0.888 - 0.989)	(0.844 - 0.951)	(0.781 - 0.918)
	0.352	0.605	0.062	0.031	0.018	0.000	0.000
Observations	44,707	44,707	44,707	44,707	44,707	44,707	44,707
AIC	39961	99714	274066	160686	839513	734485	433310
BIC	42269	102022	276374	162994	841820	736793	435617

Notes: *** p<0.01, ** p<0.05, * p<0.1; Coefficients are hazard ratios with 95% CI in parentheses, followed by p-value; Robust standard errors were used; Excluded those with age at separation missing due to small cell size.

© 2024 Reger MA et al. *JAMA Network Open.*

References

1. Hannemann CM, Hughes GJ, McCarthy JF, Katz IR. Suicide Mortality Following Veterans Crisis Line Calls by Individuals with Recent Veterans Health Administration (VHA) Use: 2010-2015. AAS Presentation. 2019.
2. Milner AJ, Carter G, Pirkis J, Robinson J, Spittal MJ. Letters, green cards, telephone calls and postcards: systematic and meta-analytic review of brief contact interventions for reducing self-harm, suicide attempts and suicide. *Br J Psychiatry*. 2015;206(3):184-190. doi:10.1192/bjp.bp.114.147819

© 2024 Reger MA et al. *JAMA Network Open.*
