# Supplementary material for: Caring Letters Sent by a Clinician or Peer to At-Risk Veterans: A Randomized Clinical Trial
Source: JAMA Netw Open. 2024 Apr 29;7(4):e248064. doi: 10.1001/jamanetworkopen.2024.8064 (PMC11059042; doi:10.1001/jamanetworkopen.2024.8064)
Supplement: Supplement 2. — eAppendix 1. Trial Protocol eAppendix 2. Statistical Analysis Plan [file jamanetwopen-e248064-s002.pdf]

Supplemental Online Content

Reger MA, Legler A, Lauver M. Caring letters sent by a clinician or peer to at-risk veterans: a randomized clinical trial. *JAMA Netw Open*. 2024;7(4):e248064. doi:10.1001/jamanetworkopen.2024.8064

eAppendix 1. Trial Protocol

eAppendix 2. Statistical Analysis

eAppendix 1. Original Protocol

ISRCTN27551361 https://doi.org/10.1186/ISRCTN27551361

Note: All dates are in the following format: Day/Month/Year

Plain English Summary

Background and study aims

US Veterans are at higher risk of suicide than the rest of the US population, and the rates of suicide among callers to the Veteran Crisis Line are especially high. Therefore, suicide prevention efforts are a high priority for this group. Caring Letters (sometimes called Caring Contacts) is one intervention that has lowered rates of suicide in prior studies. With this approach, someone (usually the person's healthcare provider) sends them letters about once a month for about a year. These letters typically let them know that others are thinking of them and wishing them well. It may seem simple, but there is a theory behind how this can reduce suicide by making people feel less socially isolated and reminding them that help is available. The evaluation for this new program for the Veteran Crisis Line (VCL) will look at whether sending Caring Letters to Veterans who contact VCL will lower the incidence of suicide attempts (as a group) compared to before the program started. It will also examine hospitalizations for mental health reasons and their use of resources like mental health care. Since Caring Letters have not been used with people who contact a crisis line before, we will be evaluating the effects of letters sent from two different people: a counselor or a Veteran peer. Everyone enrolled will receive Caring Letters, but we will randomly assign participants to receive letters from one or the other (counselor or peer) and compare the results. We will evaluate the initial set up and operation of this project for the first few years to help understand program implementation. These results will help guide how the Caring Letters program for Veterans Crisis Line callers will continue.

Who can participate?

Anyone who is a Veteran and contacts the Veterans Crisis Line from the summer of 2020 to the summer of 2021 will be included in the program evaluation as long as they are calling about themselves, not about a loved one, they are enrolled in Veterans Health Administration (VHA) care, and there is no reason to think the program would be unhelpful in terms of their medical care. Since Veterans can contact the VCL in several ways (e.g., phone call, "warm transfer" from another telephone hotline, text message communication with the VCL), Veterans from multiple forms of contact are included. Individuals who are enrolled will include all genders and all ages represented by Veterans who meet these criteria.

What does the study involve?

All individuals will receive a series of cards, sent in envelopes, wishing them well and letting them know that there are resources and people available to help them if they need anything. Half of the Veterans will receive cards that are written from a peer Veteran and the other half will receive letters from a counselor. The Veterans will

receive a total of 9 cards in one year. For the first 4 months after calling the Veterans Crisis Line, the letters will be sent every month. Then they will be sent every other month. There will be an extra card for Veterans day. After the first year of the program we will reach out to some participants (about 30) to collect information about what it was like to receive the letters and whether they found them to be helpful or caring. This information will be used to help guide program continuation. In addition, because Veterans who contact the VCL again after completing the initial Caring Letters program are likely at increased risk of suicide, half of these Veterans will receive an additional three cards every other month.

What are the possible benefits and risks of participating?
The benefits are unknown. Prior research has shown that receiving Caring Letters can reduce rates of suicide behaviors. There are few risks. If someone had a negative experience with their military service, with the VA or with their contact to the Veteran Crisis Line, they may have negative feelings about receiving letters from a provider or peer from the Veterans Crisis Line. However, if they want to opt out of receiving the letters they can request to do so. It is illegal for someone to open mail that is not addressed to them, but it is possible this could happen; therefore, it is possible an unintended person could read the Caring Letters.

Where is the study run from?
The evaluation of the VCL Caring Letters program is a partnership between investigators at the VA Puget Sound Health Care System in Seattle, WA, USA, and the Veteran Crisis Line in Canandaigua, NY, USA. Other partner sites for the evaluation of this program include the Central Arkansas Veterans Health Care System in Little Rock, AR, USA and the VA Partnered Evidence-based Policy Resource Center (PEPReC) in Boston, MA, USA.

When is the study starting and how long is it expected to run for?
April 2019 to September 2024

Who is funding the study?
This study is funded by two groups from the United States Department of Veterans Affairs (VA):
The VA Office of Mental Health and Suicide Prevention – Veterans Crisis Line and VA Quality Enhancement Research Initiative (QUERI).

Who is the main contact?
Dr Mark Reger, mark.reger@va.gov

Study website
https://www.queri.research.va.gov/centers/Caring-Letters.pdf

Contact information

Type(s)
Public

Contact name
Dr Mark Reger

ORCID ID
http://orcid.org/0000-0003-3222-9734

Contact details
VA Puget Sound Healthcare System
1660 S Columbian Way
Seattle
United States of America
98108
+1 253 583 3295
mark.reger@va.gov

Additional identifiers

EudraCT/CTIS number
Nil known

IRAS number
ClinicalTrials.gov number
Nil known

Protocol/serial number
Nil known

Study information

Randomized evaluation of a caring letters suicide prevention campaign

Study hypothesis
Aim 1: To evaluate the effects of Caring Letters on clinical outcomes and VA clinical utilization rates.
Among veteran callers to the VCL who engage in VHA care, those who receive Caring Letters will have:
1.1. A lower incidence of VA documented suicide attempts
1.2. Lower incidence and frequency of VA psychiatric hospitalization compared to veterans who called VCL in the 2 years that preceded the launch of the Caring Letters campaign
1.3. Veterans who receive Caring Letters will have higher outpatient mental health utilization rates compared to the service members who called VCL in the 2 years that preceded the program.
Exploratory Aim: To examine rates of all-cause mortality and suicide for veterans who receive Caring Letters compared to the comparison cohort of veterans from the 2 years prior to the launch of the Caring Letters campaign.

Aim 2: To study the effects of the two different Caring Letter signatories (peer and provider) on the clinical effectiveness of the intervention.
Among Veteran callers to the VCL who engage in VHA care, those who receive Caring Letters from a Peer Veteran will have:
2.1. A lower incidence and frequency of VA psychiatric hospitalization
2.2. Higher outpatient mental health utilization rates compared to veterans who receive Caring Letters from a VA Provider. When compared to the comparison cohort, the effects will be greater for those who receive Peer letters relative to VA Provider letters.

Exploratory Aim 2: To compare two types of Caring Letter signatories on rates of all-cause mortality, suicide, and suicide attempts.

Ethics approval(s)
This project has been reviewed and meets the criteria for classification as non-research. The purpose of this project is to support internal implementation and evaluation efforts to evaluate the effects of the Caring Letters suicide prevention intervention on clinical outcomes and VA clinical utilization rates for contacts to the Veterans Crisis Line. Since this is a new population for the use of the intervention, the project will also evaluate the effects of two different Caring Letter signatories (VA Counselor and a Peer Veteran) by randomizing each Veteran to one of two conditions. Furthermore, the project assesses facilitators and barriers to implementing the Caring Letters program and will include budget impact analyses. The project will involve use of secondary VA data that are collected as a part of routine care and/or clinical management. This project will be collecting information that is designed for quality improvement initiatives, as described in the Department of Veterans Affairs, Office of Research and Development Program Guide (1200.21), "VHA Operations Activities That May Constitute Research."

These activities are designed and implemented for internal VA purposes and findings are intended to be used to better inform care in the VA. This project is not designed to inform activities beyond VA, produce information that expands the knowledge base of a scientific discipline or other scholarly field, and does not involve collecting additional data or performing analyses that are not needed for the purposes of this internal implementation. This determination was confirmed by Matthew Miller, PhD, MPH, Acting Director for the Suicide Prevention Program, VHA Office of Mental Health and Suicide Prevention on February 6, 2020. Therefore, consistent with VA policy, no other review is required.

Study design
Randomised parallel trial, combined with pre-post comparison of treatment vs no treatment; Hybrid Type 1 effectiveness-implementation trial

Primary study design
Interventional

Secondary study design

Randomised parallel trial

Study setting(s)
Community

Study type(s)
Prevention

Participant information sheet
No participant information sheet available

Condition
Suicide prevention in US Veterans

Interventions
Caring Letters is a suicide prevention intervention that is well-suited for a public health approach delivered to a high-risk population. Caring Letters consist of simple expressions of care and support sent through the mail. Caring Letters directly work at the level of social support to enhance feelings of belongingness, but this model also has the potential to impact other factors by providing resources and promoting links to medical and mental health care and other services available to Veterans. This approach continues to be one of few interventions that has reduced rates of suicide in a randomized controlled trial. The Veterans Crisis Line has adopted this intervention for continued outreach to callers, and the planned evaluation will provide actionable program guidance.

A traditional approach to Caring Letters (in which a provider with an established relationship with a patient sends Caring Letters) is not feasible for this target group. Therefore, the study team will assess two different approaches. In one, letters are sent from a mental health provider that the participant has not met. The second approach will send letters from a peer veteran signatory; this is based on prior research highlighting the important role of peer support for veterans.

Each letter will be based on a template with unique messages for each mailing. Letters will be mailed out on a schedule that has been used in several successful trials, with letters mailed monthly for the first 4 months and then bi-monthly (Months 1, 2, 3, 4, 6, 8, 10, 12) with an additional card on Veterans Day. The letters and envelopes will be designed as personalized cards (colorful envelopes distinguishable from other VA correspondence or bills). A mental health resource card will be included with each letter containing information about how to access VA resources (including the Veteran Crisis Line) and free online mental health and coping resources.

Eligible callers to the Veterans Crisis Line will be identified weekly and randomized at the individual level. Callers will be randomly assigned to one of the two conditions (peer signatory, counselor signatory). Because there are fewer female than male veterans, we will stratify on sex. Within strata, we will use permuted block randomization with fixed block size to allocate participants to conditions. The random

allocation sequence will be based on a random number generator in Stata. Allocation of participants to conditions will be balanced (equal sample size in each treatment group). Letters will be mailed according to the randomized allocation and predetermined templates.

Intervention Type
Behavioural

Primary outcome measure
Suicide attempts, as measured by a record of a suicide attempts in VA suicide behavior surveillance data (reports submitted by VA providers) or ICD-10 codes during the 1-year receipt of Caring Letters. A record of a suicide attempt in either data source will indicate that the case is positive for a suicide attempt. ICD-10 codes that will be considered a suicide attempt include those associated with intentional poisoning and other intentional self-harm.

Secondary outcome measures
1. Incidence and frequency of VA psychiatric hospitalization from analysis of VA health care records data obtained from the VA's Corporate data Warehouse (CDW) assessed for 1 year during Caring Letters and 2 years pre-intervention
2. Incidence and frequency of VA emergency department visits obtained from analysis of VA health care record data from the CDW assessed for 1 year during Caring Letters and 2 years preintervention
3. Rates of outpatient VA mental health care utilization from analysis of VA health care record data from the CDW assessed for 1 year during Caring Letters and 2 years pre-intervention
4. All-cause mortality, i.e. death rate from all causes of death obtained from VA health care record data from the CDW assessed for 1 year during Caring Letters and 2 years pre-intervention
5. Rates of calls to the VCL and VA311 (help line included in the Caring Letters)assessed for 1 year during Caring Letters and 2 years pre-intervention
6. Rates of suicide: Suicide mortality rates will be obtained from the VA/DoD Mortality Data Repository which contains the National Death Index (state death records data) for all veterans assessed for 1 year during Caring Letters and 2 years pre-intervention. Since this data is not available until about 2 years after the year of death, these results will be delayed compared to other analyses.
A secondary goal for this study is to collect data on the delivery of the intervention to inform potential improvements for implementation. The project will assess facilitators and barriers to implementing the Caring Letters program and will include budget impact analyses. The RE-AIM analytic framework will be used to examine implementation and impact of the intervention.

Overall study start date
01/04/2019

Overall study end date
30/09/2024

Eligibility

Participant inclusion criteria
Participants will be included if they call the Veterans Crisis Line during the recruitment period (estimated to be 6/1/2020 – 5/30/2022) and:
1. Are an identifiable VCL caller (i.e. not an anonymous caller)
2. Have a valid mailing address on file with the VA
3. Are calling about themselves (i.e. not calling about a loved one)
All veteran age ranges and genders will be included.

Participant type(s)
Other

Age group
Adult

Sex
Both

Target number of participants
100,000

Participant exclusion criteria
1. No fixed valid mailing address available in VA records
2. Not identifiable (e.g. full SSN not known; last 4 digits of the SNN and name not available)
3. Not a veteran (e.g. someone calling on behalf of a veteran)
4. Has not received Veterans Health Administration (VHA) care
5. The intervention is thought to be counter-productive to clinical goals (e.g. poor boundaries
with VCL staff)

Locations

Countries of recruitment
United States Minor Outlying Islands

United States of America

Study participating centre
VA Puget Sound Healthcare System
1660 S Columbian Way
Seattle

United States of America
98108

Study participating centre
Partnered Evidence-Based Policy Resource Center (PEPReC) / VA Boston Healthcare System
Research & Development
150 South Huntington Avenue
Boston
United States of America
02130

Study participating centre
Central Arkansas Veterans Healthcare System
2200 Fort Roots Drive
Little Rock
United States of America
72114

Study participating centre
Veterans Crisis Line
400 Fort Hill Avenue
Canandaigua
United States of America
14424

Sponsor information

Organisation
VA Puget Sound Healthcare System

Sponsor details
1660 S Columbian Way
Seattle
United States of America
98108
+1 206 764 2848
Jane.summerfield@va.gov

Sponsor type
Hospital/treatment centre

Website
https://www.pugetsound.va.gov/

Organisation

Veterans Crisis Line
Sponsor details
400 Fort Hill Avenue
Canandaigua
United States of America
14424
+1 352 240 2748
vhavclcaringlettersprogram@va.gov

Sponsor type
Government

Website
https://www.veteranscrisisline.net/

Organisation
Quality Enhancement Research Initiative (QUERI)

Sponsor details
1100 1st Street NE, Suite 6
Washington, DC
United States of America
20002
(202) 443-5818
vacoqueri@va.gov

Sponsor type
Government

Website
https://www.queri.research.va.gov/

Funder(s)

Funder type
Government

Funder Name
U.S. Department of Veterans Affairs

Alternative Name(s)
Department of Veterans Affairs, United States Department of Veterans Affairs, US Department of Veterans Affairs, U.S. Dept. of Veterans Affairs, Veterans Affairs, Veterans Affairs Department, VA, USDVA

Funding Body Type

Government organisation

Funding Body Subtype
National government

Location
United States of America

Funder Name
The VA Office of Mental Health and Suicide Prevention – Veterans Crisis Line

Final Protocol

ISRCTN27551361 https://doi.org/10.1186/ISRCTN27551361

Note: All dates are in the following format: Day/Month/Year

Plain English Summary

Background and study aims

US Veterans are at higher risk of suicide than the rest of the US population, and the rates of suicide among callers to the Veteran Crisis Line are especially high. Therefore, suicide prevention efforts are a high priority for this group. Caring Letters (sometimes called Caring Contacts) is one intervention that has lowered rates of suicide in prior studies. With this approach, someone (usually the person's healthcare provider) sends them letters about once a month for about a year. These letters typically let them know that others are thinking of them and wishing them well. It may seem simple, but there is a theory behind how this can reduce suicide by making people feel less socially isolated and reminding them that help is available. The evaluation for this new program for the Veteran Crisis Line (VCL) will look at whether sending Caring Letters to Veterans who contact VCL will lower the incidence of suicide attempts (as a group) compared to before the program started. It will also examine hospitalizations for mental health reasons and their use of resources like mental health care. Since Caring Letters have not been used with people who contact a crisis line before, we will be evaluating the effects of letters sent from two different people: a counselor or a Veteran peer. Everyone enrolled will receive Caring Letters, but we will randomly assign participants to receive letters from one or the other (counselor or peer) and compare the results. We will evaluate the initial set up and operation of this project for the first few years to help understand program implementation. These results will help guide how the Caring Letters program for Veterans Crisis Line callers will continue.

Who can participate?

Anyone who is a Veteran and contacts the Veterans Crisis Line from the summer of 2020 to the summer of 2021 will be included in the program evaluation as long as they are calling about themselves, not about a loved one, they are enrolled in Veterans Health Administration (VHA) care, and there is no reason to think the program would be unhelpful in terms of their medical care. Since Veterans can contact the VCL in several ways (e.g., phone call, "warm transfer" from another telephone hotline, text message communication with the VCL), Veterans from multiple forms of contact are included. Individuals who are enrolled will include all genders and all ages represented by Veterans who meet these criteria.

What does the study involve?

All individuals will receive a series of cards, sent in envelopes, wishing them well and letting them know that there are resources and people available to help them if they need anything. Half of the Veterans will receive cards that are written from a peer Veteran and the other half will receive letters from a counselor. The Veterans will receive a total of 9 cards in one year. For the first 4 months after calling the Veterans Crisis Line, the letters will be sent every month. Then they will be sent every other

month. There will be an extra card for Veterans day. After the first year of the program we will reach out to some participants (about 30) to collect information about what it was like to receive the letters and whether they found them to be helpful or caring. This information will be used to help guide program continuation. In addition, because Veterans who contact the VCL again after completing the initial Caring Letters program are likely at increased risk of suicide, half of these Veterans will receive an additional three cards every other month.

What are the possible benefits and risks of participating?
The benefits are unknown. Prior research has shown that receiving Caring Letters can reduce rates of suicide behaviors. There are few risks. If someone had a negative experience with their military service, with the VA or with their contact to the Veteran Crisis Line, they may have negative feelings about receiving letters from a provider or peer from the Veterans Crisis Line. However, if they want to opt out of receiving the letters they can request to do so. It is illegal for someone to open mail that is not addressed to them, but it is possible this could happen; therefore, it is possible an unintended person could read the Caring Letters.

Where is the study run from?
The evaluation of the VCL Caring Letters program is a partnership between investigators at the VA Puget Sound Health Care System in Seattle, WA, USA, and the Veteran Crisis Line in Canandaigua, NY, USA. Other partner sites for the evaluation of this program include the Central Arkansas Veterans Health Care System in Little Rock, AR, USA and the VA Partnered Evidence-based Policy Resource Center (PEPReC) in Boston, MA, USA.

When is the study starting and how long is it expected to run for?
April 2019 to September 2024

Who is funding the study?
This study is funded by two groups from the United States Department of Veterans Affairs (VA):
The VA Office of Mental Health and Suicide Prevention – Veterans Crisis Line and VA Quality Enhancement Research Initiative (QUERI).

Who is the main contact?
Dr Mark Reger, mark.reger@va.gov

Study website
https://www.queri.research.va.gov/centers/Caring-Letters.pdf

Contact information

Type(s)
Public

Contact name

Dr Mark Reger

ORCID ID
http://orcid.org/0000-0003-3222-9734

Contact details
VA Puget Sound Healthcare System
1660 S Columbian Way
Seattle
United States of America
98108
+1 253 583 3295
mark.reger@va.gov

Additional identifiers

EudraCT/CTIS number
Nil known

IRAS number

ClinicalTrials.gov number
Nil known

Protocol/serial number
Nil known

Study information

Randomized evaluation of a caring letters suicide prevention campaign

Study hypothesis
Aim 1: To evaluate the effects of Caring Letters on clinical outcomes and VA clinical utilization rates.
Among Veteran contacts to the VCL who engage in VHA care, those who receive Caring Letters will have:
1.1. A lower incidence of VA documented suicide attempts
1.2. Lower incidence and frequency of VA psychiatric hospitalization compared to Veterans who contacted VCL in the 2 years that preceded the launch of the Caring Letters campaign
1.3. Veterans who receive Caring Letters will have higher outpatient mental health utilization rates compared to the service members who contacted VCL in the 2 years that preceded the program.
1.4. After completing one year of Caring Letters, Veterans who contact the VCL again and receive additional Caring Letters will have a higher proportion of VA mental

health outpatient visits compared to Veterans who contact the VCL again and do not receive the additional Caring Letters.

Exploratory Aim: To examine rates of all-cause mortality and suicide for Veterans who receive Caring Letters compared to the comparison cohort of Veterans from the 2 years prior to the launch of the Caring Letters campaign.

Aim 2: To study the effects of the two different Caring Letter signatories (peer and provider) on the clinical effectiveness of the intervention.
Among Veterans who contact the VCL and engage in VHA care, those who receive Caring Letters from a Peer Veteran will have:
2.1. A lower incidence and frequency of VA psychiatric hospitalization
2.2. Higher outpatient mental health utilization rates compared to Veterans who receive Caring Letters from a VA Provider. When compared to the comparison cohort, the effects will be greater for those who receive Peer letters relative to VA Provider letters.

Exploratory Aim 2: To compare two types of Caring Letter signatories on rates of all-cause mortality, suicide and suicide attempts.

Ethics approval(s)
This project has been reviewed and meets the criteria for classification as non-research. The purpose of this project is to support internal implementation and evaluation efforts to evaluate the effects of the Caring Letters suicide prevention intervention on clinical outcomes and VA clinical utilization rates for contacts to the Veterans Crisis Line. Since this is a new population for the use of the intervention, the project will also evaluate the effects of two different Caring Letter signatories (VA Counselor and a Peer Veteran) by randomizing each Veteran to one of two conditions. Furthermore, the project assesses facilitators and barriers to implementing the Caring Letters program and will include budget impact analyses. The project will involve use of secondary VA data that are collected as a part of routine care and/or clinical management. This project will be collecting information that is designed for quality improvement initiatives, as described in the Department of Veterans Affairs, Office of Research and Development Program Guide (1200.21), "VHA Operations Activities That May Constitute Research."

These activities are designed and implemented for internal VA purposes and findings are intended to be used to better inform care in the VA. This project is not designed to inform activities beyond VA, produce information that expands the knowledge base of a scientific discipline or other scholarly field, and does not involve collecting additional data or performing analyses that are not needed for the purposes of this internal implementation. This determination was confirmed by Matthew Miller, PhD, MPH, Acting Director for the Suicide Prevention Program, VHA Office of Mental Health and Suicide Prevention on February 6, 2020. Therefore, consistent with VA policy, no other review is required.

Study design

Randomised parallel trial, combined with pre-post comparison of treatment vs no treatment; Hybrid Type 1 effectiveness-implementation trial

Primary study design
Interventional

Secondary study design
Randomised parallel trial

Study setting(s)
Community

Study type(s)
Prevention

Participant information sheet
No participant information sheet available

Condition
Suicide prevention in US Veterans

Interventions
Caring Letters is a suicide prevention intervention that is well-suited for a public health approach delivered to a high-risk population. Caring Letters consist of simple expressions of care and support sent through the mail. Caring Letters directly work at the level of social support to enhance feelings of belongingness, but this model also has the potential to impact other factors by providing resources and promoting links to medical and mental health care and other services available to Veterans. This approach continues to be one of few interventions that has reduced rates of suicide in a randomized controlled trial. The Veterans Crisis Line has adopted this intervention for continued outreach to Veterans, and the planned evaluation will provide actionable program guidance.

A traditional approach to Caring Letters (in which a provider with an established relationship with a patient sends Caring Letters) is not feasible for this target group. Therefore, the study team will assess two different approaches. In one, letters are sent from a mental health provider that the participant has not met. The second approach will send letters from a peer Veteran signatory; thus inclusion of peer signed notes is based on prior research highlighting the important role of peer support for Veterans.

Each letter will be based on a template with unique messages for each mailing. Letters will be mailed out on a schedule that has been used in several successful trials, with letters mailed monthly for the first 4 months and then bi-monthly (Months 1, 2, 3, 4, 6, 8, 10, 12) with an additional card on Veterans Day. After the initial Caring Letters intervention, Veterans with repeat contact with the VCL will receive letters on months 1, 3, 5 after re-engaging with VCL. The letters and envelopes will be designed

as personalized cards (colorful envelopes distinguishable from other VA correspondence or bills). A mental health resource card will be included with each letter containing information about how to access VA resources (including the Veteran Crisis Line) and free online mental health and coping resources.

Eligible Veterans will be identified weekly and randomized at the individual level. Veterans will be randomly assigned to one of the two conditions (peer signatory, counselor signatory). Repeat contacts to the VCL will be randomized to one of two conditions (additional Caring Letters, no additional Caring Letters). Because there are fewer female than male Veterans, we will stratify on gender. Within strata, we will use permuted block randomization with fixed block size to allocate participants to conditions. The random allocation sequence will be based on a random number generator in Stata. Allocation of participants to conditions will be balanced (equal sample size in each treatment group). Letters will be mailed according to the randomized allocation and pre-determined templates.

Intervention Type
Behavioural

Primary outcome measure
Suicide attempts, as measured by a record of a suicide attempts in VA suicide behavior surveillance data (reports submitted by VA providers) or ICD-10 codes during the 1-year receipt of Caring Letters. A record of a suicide attempt in either data source will indicate that the case is positive for a suicide attempt. ICD-10 codes that will be considered a suicide attempt include those associated with intentional poisoning and other intentional self-harm.

Secondary outcome measures
1. Incidence and frequency of VA psychiatric hospitalization from analysis of VA health care records data obtained from the VA's Corporate data Warehouse (CDW) assessed for 1 year during Caring Letters and 2 years pre-intervention
2. Incidence and frequency of VA emergency department visits obtained from analysis of VA health care record data from the CDW assessed for 1 year during Caring Letters and 2 years preintervention
3. Rates of outpatient VA mental health care utilization from analysis of VA health care record data from the CDW assessed for 1 year during Caring Letters and 2 years pre-intervention
4. All-cause mortality, i.e., death rate from all causes of death obtained from VA health care record data from the CDW assessed for 1 year during Caring Letters and 2 years pre-intervention
5. Rates of calls to the VCL and VA411 (help line included in the Caring Letters) assessed for 1 year during Caring Letters and 2 years pre-intervention
6. Rates of suicide: Suicide mortality rates will be obtained from the VA/DoD Mortality Data Repository which contains the National Death Index (state death records data) for all Veterans assessed for 1 year during Caring Letters and 2 years pre-intervention. Since these data are not available until about 2 years after the year of death, these results will be delayed compared to other analyses.

7. Measures described in 1-6 above will also be compared between Veterans who contact the VCL again and receive additional Caring Letters, and Veterans who contact the VCL again and do not receive the additional Caring Letters.
A secondary goal for this study is to collect data on the delivery of the intervention to inform potential improvements for implementation. The project will assess facilitators and barriers to implementing the Caring Letters program and will include budget impact analyses. The RE-AIM analytic framework will be used to examine implementation and impact of the intervention.

Overall study start date
01/04/2019

Overall study end date
30/09/2024

Eligibility

Participant inclusion criteria
Participants will be included if they call the Veterans Crisis Line during the recruitment period (11/06/2020 – 10/06/2021) and:
1. Are an identifiable VCL contact (i.e., not an anonymous contact)
2. Have a valid mailing address on file with the VA
3. Are contacting VCL about themselves (i.e., not contacting VCL about a loved one)
All Veterans who complete the initial intervention and contact the VCL again will be considered for the additional Caring Letters.
All Veteran age ranges and genders will be included.

Participant type(s)
Other

Age group
Adult

Sex
Both

Target number of participants
100,000

Total final enrolment
102365

Participant exclusion criteria
1. No fixed valid mailing address available in VA records
2. Not identifiable (e.g., full SSN not known; last 4 digits of the SSN and name not available)

3. Not a Veteran (e.g., someone calling on behalf of a Veteran)
4. Has not received Veterans Health Administration (VHA) care

Recruitment start date
11/06/2020

Recruitment end date
10/06/2021

Locations

Countries of recruitment
United States Minor Outlying Islands

United States of America

Study participating centre
VA Puget Sound Healthcare System
1660 S Columbian Way
Seattle
United States of America
98108

Study participating centre
Partnered Evidence-Based Policy Resource Center (PEPReC) / VA Boston Healthcare System
Research & Development
150 South Huntington Avenue
Boston
United States of America
02130

Study participating centre
Central Arkansas Veterans Healthcare System
2200 Fort Roots Drive
Little Rock
United States of America
72114

Study participating centre
Veterans Crisis Line
400 Fort Hill Avenue
Canandaigua
United States of America
14424

Sponsor information

Organisation
VA Puget Sound Healthcare System

Sponsor details
1660 S Columbian Way
Seattle
United States of America
98108
+1 206 764 2848
Jane.summerfield@va.gov

Sponsor type
Hospital/treatment centre

Website
https://www.pugetsound.va.gov/

Organisation
Veterans Crisis Line
Sponsor details
400 Fort Hill Avenue
Canandaigua
United States of America
14424
+1 352 240 2748
vhavclcaringlettersprogram@va.gov

Sponsor type
Government

Website
https://www.veteranscrisisline.net/

Organisation
Quality Enhancement Research Initiative (QUERI)

Sponsor details
1100 1st Street NE, Suite 6
Washington, DC
United States of America
20002
(202) 443-5818
vacoqueri@va.gov

Sponsor type
Government

Website
https://www.queri.research.va.gov/

Funder(s)

Funder type
Government

Funder Name
U.S. Department of Veterans Affairs

Alternative Name(s)
Department of Veterans Affairs, United States Department of Veterans Affairs, US Department of Veterans Affairs, U.S. Dept. of Veterans Affairs, Veterans Affairs, Veterans Affairs Department, VA, USDVA

Funding Body Type
Government organisation

Funding Body Subtype
National government

Location
United States of America

Funder Name
The VA Office of Mental Health and Suicide Prevention – Veterans Crisis Line

eAppendix 2. Statistical Analysis Plan

Table of Contents

p. 2 Overview

p. 4 SAP Version 1 (11/6/20)

p. 9 SAP Version 2 (4/2/22)

p. 15 SAP Version 3(6/22/23)

Overview

This statistical analysis plan (SAP) builds upon the details included in the ISCRTN protocol (ISRCTN27551361). Initial analysis details were documented before outcome data was available for the study (version 1: November 6, 2020). The SAP includes plans to analyze both the randomized signatory component as well as the observational data on outcomes associated with receipt of letters (regardless of signatory).

The analysis plans for the observational component have evolved over time. In our original (11/6/20) SAP, we documented potential strategies to assess threats to internal validity, including the beginning of the COVID-19 pandemic, in our analysis of the association between receipt of caring letters and our primary and secondary outcomes. These strategies included identification of a viable instrumental variable that would be associated with timing of initial letter receipt but not outcome. We were unable to identify a strong, valid instrument and instead designed a difference in difference-in-differences (triple differences) approach that would compare changes from before to after a Veterans Crisis Line (VCL) call among a) Veterans who received letters (called between 6/20 and 6/21), b) Veterans who did not receive letters but called during the beginning of COVID lockdowns (called between 3/20 and 5/20), and c) Veterans who did not receive letters and called a year before COVID lockdowns (allowing us to observe outcomes in a pre-pandemic period; called between 6/18 and 3/19). This is documented in version 2 of the SAP (4/2/22) and was also published in the appendix of our paper describing the development and initial stages of the caring letters evaluation.[1]

When we implemented the triple differences approach after data collection was complete in June 2022, we learned that the assumptions behind a difference-in-differences approach were not supported in our data. Trends in outcomes were not parallel before the index call across our different treatment groups. Therefore, in January 2023, we pivoted to using time-to-event analyses and cause-specific hazard functions that include controls for year, month, and onset of the COVID pandemic. The cohort was reframed to include index calls between 6/11/2018 and 6/10/2021. This is the approach reported in the manuscript.

Further adjustments to the analytic sample, measures, and analyses are summarized below and documented in version 3 of the SAP, 6/22/23.

Analytic Sample

During the course of the study, we identified elements of the VCL Medora dataset that would allow us to more accurately verify our sample matched the goals of our inclusion criteria (Veterans who contacted VCL on behalf of themselves and communicated with a VCL responder). Therefore, from the sample of individuals presumed to receive at least one letter, we additionally excluded third party contacts (a variable that was not available at the beginning of the study, when the code to identify letter recipients was created), as well as calls in which a responder made an outgoing call to a Veteran and was unable to make contact, and calls in which the source of the VCL contact was an email referral, compassionate care, caregiver, Facebook, or assigned callback from our treatment group. This was an analytic refinement and not a change in the goals of the study.

Measures

We dropped both subsequent VCL call and calls to VA411 (help line included in the Caring Letters) as secondary outcomes. Subsequent VCL calls were dropped because a substantial proportion of VCL callers contact the VCL frequently — sometimes multiple times a day. Without knowing the timing of letter receipt, analyzing the association between multiple VCL calls and receipt of letters is unlikely to be informative. Calls to VA411 were dropped because data on call volume were unavailable. We also did not pursue suicidal ideation as an outcome, as it is unreliably documented in the medical record. We focus on suicide attempt incidence rather than frequency, because of concerns about the reliability of event frequency data. Suicide mortality remains an outcome of interest, but cause of death data are not yet available for the entire sample.

One covariate – rank – was not readily available and was not included in our final analyses. Data on active vs reserve status was missing for 41% of our sample and was thus not included. We also considered using years of service, but it was highly correlated with age at separation from service (Pearson correlation coefficient = 0.6) and was not included. Marital status was added as a covariate. We extended the lookback period for Elixhauser comorbidities from one year to two years to account for fewer opportunities for comorbidity ascertainment during the height of the COVID-19 pandemic.

Analyses

In the randomized signatory analyses, we did not observe any significant relationship between signatory and any of our primary or secondary outcomes in unadjusted analyses, so we refrained from running adjusted regression models for numbers of visits or Cox regressions to explore associations between caring letters and all-cause mortality. Details of our final analyses are documented in version 3 of the SAP (6/22/23).

1. Reger MA, Lauver MG, Manchester C, Abraham TH, Landes SJ, Garrido MM, Griffin C, Woods JA, Strombotne KL, Hughes G. Development of the Veterans Crisis Line caring letters suicide prevention intervention. Health Services Research 2022; 57(S1): 42-52. doi: 10.1111/1475-6773.13985

Randomized Evaluation of the VCL Caring Letters Suicide Prevention Campaign: Plan for Quantitative Analysis VERSION 1

Last Updated: November 6, 2020

Evaluation Overview | Quantitative Analysis Overview | Data Flow

Evaluation Overview

Goal: To test the effectiveness of a Caring Letters campaign on clinical outcomes and utilization rates in order to identify an effective and sustainable evidence-based practice to reduce suicide behaviors among veterans.

Eligible Cohort: All VCL callers with an identifiable address in VHA databases. The following callers will be excluded: (1) friends and family calling on behalf of a veteran, (2) civilians, and (3) callers who have died prior to being randomized. Furthermore, individuals who call the hotline after enrollment in the study will be flagged so as not to be re-randomized. Individuals who die after enrollment in the study will be flagged so as not to continue receiving letters.

Treatment/Exposures: A control group will be constructed from VCL callers two years prior to the trial start date. A total of nine (9) letters will be sent to the callers over the course of the intervention. One (1) letter will be sent to all callers on Veteran's Day, and an additional eight (8) letters will be mailed to each caller, each spaced approximately one month apart beginning from the initial call date.[1] Eligible veterans who call the VCL after the trial start date will be randomized to one of two interventions, detailed below.

Arm 1: Peer signatory letter
Arm 2: Provider signatory letter

Randomization: To ensure adequate gender representation in the peer signatory conditions, randomization will be conducted using permuted block randomization, stratified by gender. New callers will be identified monthly; randomization will occur monthly using blocks of 4 patients with 2 conditions (A=peer signatory, B=provider signatory). This set up allows for 6 permutations ((1-AABB, 2-ABAB, 3-ABBA, 4-BAAB, 5-BABA, 6-BBAA)).

After callers are successfully matched to CDW data, they are stratified by gender. Within each stratum, a uniform random number will be generated in SQL (range 0-1) that corresponds to each block of 4 patients. If the random number, R, is >= 0 and < 1/6, the block of 4 callers is assigned to permutation 1 (order of assignment to letter type is AABB). If 1/6 <= R < 2/6, the

[1] The specific months will be 1,2,3,4,6,8,10,12 + Veteran's Day card

block of 4 callers is assigned to permutation 2 (order of assignment to letter type is ABAB). The same pattern follows for permutations 3-6.

Data: The analytic file will be compiled from the following data below. Specific variables are detailed in the sections that follow.

- VCL Medora data
- CDW database
- SSA Vital Status file
- Mortality Data Repository
- OMHSP Standard Suicide Overdose Event Table (encompassing both SPAN and its replacement – the Suicide Behavior and Overdose Report [SBOR])
- 311 data

Outcomes: The outcomes for the study are listed below, along with data source, in Table 1.

Table 1. Study Outcomes

Clinical Outcomes	VHA Utilization (Incidence & Frequency)	VCL Utilization (Incidence & Frequency)	Others
• All-cause mortality (*CDW Spatient Table*, *Vital Status file*)<br>• Suicide mortality (*Mortality data repository*)<br>• Non-fatal suicide event (incidence & frequency) (*ICD-10 codes and OMHSP_Standard_SuicideOverdoseEvent Table*)<br>• Suicidal ideation (*OMHSP_Standard_SuicideOverdoseEvent Table*)	• Any VA healthcare services (*CDW*)<br>• Psychiatric hospitalization (*CDW*)<br>• Outpatient mental health services (*CDW*)<br>• ED visits (*CDW*)	• Subsequent VCL call (*Medora data*)	• 311 aggregate monthly call volume (external 311 partner data)

Additional variables (covariates):

- Sociodemographics
    - Age
    - Gender
    - Race/ethnicity
    - Branch of military
    - Discharge status
    - Years of service
    - Age at separation
    - Active or reserve component
    - Rank

    - VAMC
- Comorbidities in past year
    - Mental illness diagnoses
    - Elixhauser comorbidities
- Past SAEs
- Past mental health utilization
- Exploratory: Other covariates in the VCL caller data; REACH-VET risk score

Quantitative Analysis Overview

The primary analyses will be performed after the final letters are sent, and after monitoring and cleaning of data collected from all callers. Main analyses will be performed according to the intention-to-treat (ITT) principle, in which all callers are analyzed according to their initially assigned study arm at baseline, regardless of whether letters were received.

Data structure: Patient-month level.

Analysis 1: Pre-Post Comparison: The pre-post analysis will compare outcomes between callers who receive Caring Letters compared to callers in the previous two years. We will test the following three hypotheses:

- H1: lower incidence of VA documented suicide attempts in the post period.
- H2: lower incidence and frequency of VA psychiatric hospitalization in the post period.
- H3: higher rates of engagement in VA healthcare (in general) and higher outpatient mental health utilization rates in the post period.

We will analyze differences in proportions of VCL callers who engaged with any outpatient VA health care before and after the Caring Letters campaign with a chi-square test. Similarly, chi-square tests will be used to examine differences in incidence of suicide attempts, incidence of psychiatric hospitalization, and incidence of mental health outpatient visits. Differences in frequency of hospitalizations and mental health outpatient visits will be examined with Wilcoxon rank-sum tests. Pre-post analyses of associations among outcomes and Caring Letters will not be able to rule out the possibility that observed differences in outcomes are due to unmeasured temporal changes coinciding with Caring Letters.

<u>Potential threats to internal validity may include the following scenarios</u>:

1. Historical differences in outcomes pre- and post-trial start date. These may include overall trends or Covid-19 specific differences (especially in March 2020 and beyond).

2. Historical differences in caller composition (and thus likelihood of experiencing an outcome). These may include overall trends in callers, or Covid-19 specific trends in callers.
3. Policy changes that coincide with the intervention.

Potential strategies to assess and address threats to internal validity:

1. Examining monthly trends in outcomes over time.
2. Examining overall differences and monthly trends in caller characteristics. We will pull three years of VCL caller history and explore whether patterns of repeat calls and caller sociodemographic and clinical characteristics evolve over time.
3. Identification of alternate contemporaneous comparison groups
4. Identify a potential instrumental variable (variable associated with letter receipt but not with health care utilization or mortality). One potential instrument may be delays in mail delivery that lead to variation in timing of initial letter receipt? We can look at the date between call and bad-address notification from print vendor as a proxy.

Analysis 2: Comparison of Letter Signatories

Our Aim 2 analyses (cross-arm analyses among individuals engaged in VA care) will not be subject to the history threats to internal validity that exist in Aim 1. We can further isolate the impact of caring letter signatory on outcomes by controlling for the potential confounders noted above, in addition to confounders with potential prognostic value, such as mental health diagnoses (Kahan 2014). Dichotomous outcomes will be examined with logistic regression, and counts of hospitalizations and visits will be examined with zero-inflated Poisson or negative binomial models. Exploratory analyses of associations among Caring Letters and all-cause and suicide mortality will be conducted with Cox regression (using time of VCL call as time 0).

Budget Impact Analysis (BIA):

We will examine the budget impact of Caring Letters among all VCL callers over a 12-month period. We will compare costs related to inpatient and outpatient mental health care use and medications before the Caring Letters campaign with costs related to health care use and medications, as well as costs of mailing the letters during the Caring Letters campaign. We expect inpatient costs to decrease and outpatient costs to increase due to the intervention. We do not expect Caring Letters to serve as a substitute for any existing mental health outreach program. Costs of both VA-provided and purchased care will be captured. In all analyses, we will use the Gross Domestic Product Deflator to adjust costs to 2020 dollars. The budget impact of each version of Caring Letters will be assessed. Mean costs per patient-month alive will be reported.

Data Flow

VCL Data is downloaded from backups on a weekly basis. The data is downloaded from the Saturday two weeks prior through the end of the Friday of the previous week. That data is then uploaded to the Caring Letters data system that resided on CDWA01. A SQL script is then run to parse the new data, identify callers who can be matched to the CDW and enroll them into the program, then generates a list of all letters due to go out this week.

This includes letters for newly enrolled patients as well as patients enrolled previously who are getting subsequent letters. Once the letter file has been generated it is sent to the print vendor using encrypted email. The print vendor then sends back information on any patients with a change of address, any addresses that are undeliverable and the mail receipt of when each batch of letters was sent out.

Randomized Evaluation of the VCL Caring Letters Suicide Prevention Campaign:
Plan for Quantitative Analysis
VERSION 2

Last Updated: April 1, 2022

Evaluation Overview | Quantitative Analysis Overview | Data Flow

Evaluation Overview

Goal: To test the effectiveness of a Caring Letters campaign on clinical outcomes and utilization rates in order to identify an effective and sustainable evidence-based practice to reduce suicide behaviors among veterans.

Eligible Cohort: All VCL callers with an identifiable address in VHA databases. The following callers will be excluded: (1) friends and family calling on behalf of a veteran, (2) civilians, and (3) callers who have died prior to being randomized. Furthermore, individuals who call the hotline after enrollment in the study will be flagged so as not to be re-randomized. Individuals who die after enrollment in the study will be flagged so as not to continue receiving letters.

Treatment/Exposures: A control group will be constructed from VCL callers two years prior to the trial start date. A total of nine (9) letters will be sent to the callers over the course of the intervention. One (1) letter will be sent to all callers on Veteran's Day, and an additional eight (8) letters will be mailed to each caller, each spaced approximately one month apart beginning from the initial call date.[2] Eligible veterans who call the VCL after the trial start date will be randomized to one of two interventions, detailed below.

Arm 1: Peer signatory letter
Arm 2: Provider signatory letter

Randomization: To ensure adequate gender representation in the peer signatory conditions, randomization will be conducted using permuted block randomization, stratified by gender. New callers will be identified monthly; randomization will occur monthly using blocks of 4 patients with 2 conditions (A=peer signatory, B=provider signatory). This set up allows for 6 permutations ((1-AABB, 2-ABAB, 3-ABBA, 4-BAAB, 5-BABA, 6-BBAA)).

After callers are successfully matched to CDW data, they are stratified by gender. Within each stratum, a uniform random number will be generated in SQL (range 0-1) that corresponds to each block of 4 patients. If the random number, R, is >= 0 and < 1/6, the block of 4 callers is assigned to permutation 1 (order of assignment to letter type is AABB). If 1/6 <= R < 2/6, the

[2] The specific months will be 1,2,3,4,6,8,10,12 + Veteran's Day card

block of 4 callers is assigned to permutation 2 (order of assignment to letter type is ABAB). The same pattern follows for permutations 3-6.

Data: The analytic file will be compiled from the following data below. Specific variables are detailed in the sections that follow.

- VCL Medora data
- CDW database
- SSA Vital Status file
- Mortality Data Repository
- OMHSP Standard Suicide Overdose Event Table (encompassing both SPAN and its replacement – the Suicide Behavior and Overdose Report [SBOR])
- 311 data

Outcomes: The outcomes for the study are listed below, along with data source, in Table 1.

Table 1. Study Outcomes

Clinical Outcomes	VHA Utilization (Incidence & Frequency)	VCL Utilization (Incidence & Frequency)	Others
• All-cause mortality (*CDW Spatient Table*, *Vital Status file*)<br>• Suicide mortality (*Mortality data repository*)<br>• Non-fatal suicide event (incidence & frequency) (*ICD-10 codes and OMHSP_Standard_SuicideOverdoseEvent Table*)<br>• Suicidal ideation (*OMHSP_Standard_SuicideOverdoseEvent Table*)	• Any VA healthcare services (*CDW*)<br>• Psychiatric hospitalization (*CDW*)<br>• Outpatient mental health services (*CDW*)<br>• ED visits (*CDW*)	• Subsequent VCL call (*Medora data*)	• 311 aggregate monthly call volume (external 311 partner data)

Additional variables (covariates):

- Sociodemographics
  - Age
  - Gender
  - Race/ethnicity
  - Branch of military
  - Discharge status
  - Years of service
  - Age at separation
  - Active or reserve component
  - Rank

    - VAMC
- Comorbidities in past year
    - Mental illness diagnoses
    - Elixhauser comorbidities
- Past SAEs
- Past mental health utilization
- Exploratory: Other covariates in the VCL caller data; REACH-VET risk score

Quantitative Analysis Overview

The primary analyses will be performed after the final letters are sent, and after monitoring and cleaning of data collected from all callers. Main analyses will be performed according to the intention-to-treat (ITT) principle, in which all callers are analyzed according to their initially assigned study arm at baseline, regardless of whether letters were received.

Data structure: Patient-month level.

Analysis 1: Pre-Post Comparison: The pre-post analysis will compare outcomes between callers who receive Caring Letters compared to callers in the previous two years. We will test the following three hypotheses:

- H1: lower incidence of VA documented suicide attempts in the post period.
- H2: lower incidence and frequency of VA psychiatric hospitalization in the post period.
- H3: higher rates of engagement in VA healthcare (in general) and higher outpatient mental health utilization rates in the post period.

We will analyze differences in proportions of VCL callers who engaged with any outpatient VA health care before and after the Caring Letters campaign with a chi-square test. Similarly, chi-square tests will be used to examine differences in incidence of suicide attempts, incidence of psychiatric hospitalization, and incidence of mental health outpatient visits. Differences in frequency of hospitalizations and mental health outpatient visits will be examined with Wilcoxon rank-sum tests. Pre-post analyses of associations among outcomes and Caring Letters will not be able to rule out the possibility that observed differences in outcomes are due to unmeasured temporal changes coinciding with Caring Letters.

<u>Potential threats to internal validity may include the following scenarios</u>:

4. Historical differences in outcomes pre- and post-trial start date. These may include overall trends or Covid-19 specific differences (especially in March 2020 and beyond).

5. Historical differences in caller composition (and thus likelihood of experiencing an outcome). These may include overall trends in callers, or Covid-19 specific trends in callers.
6. Policy changes that coincide with the intervention.

Potential strategies to assess and address threats to internal validity:

5. Examining monthly trends in outcomes over time.
6. Examining overall differences and monthly trends in caller characteristics. We will pull three years of VCL caller history and explore whether patterns of repeat calls and caller sociodemographic and clinical characteristics evolve over time.
7. Identification of alternate contemporaneous comparison groups
8. Identify a potential instrumental variable (variable associated with letter receipt but not with health care utilization or mortality). One potential instrument may be delays in mail delivery that lead to variation in timing of initial letter receipt? We can look at the date between call and bad-address notification from print vendor as a proxy.

Analysis 2: Comparison of Letter Signatories

Our Aim 2 analyses (cross-arm analyses among individuals engaged in VA care) will not be subject to the history threats to internal validity that exist in Aim 1. We can further isolate the impact of caring letter signatory on outcomes by controlling for the potential confounders noted above, in addition to confounders with potential prognostic value, such as mental health diagnoses (Kahan 2014). Dichotomous outcomes will be examined with logistic regression, and counts of hospitalizations and visits will be examined with zero-inflated Poisson or negative binomial models. Exploratory analyses of associations among Caring Letters and all-cause and suicide mortality will be conducted with Cox regression (using time of VCL call as time 0).

Budget Impact Analysis (BIA):

We will examine the budget impact of Caring Letters among all VCL callers over a 12-month period. We will compare costs related to inpatient and outpatient mental health care use and medications before the Caring Letters campaign with costs related to health care use and medications, as well as costs of mailing the letters during the Caring Letters campaign. We expect inpatient costs to decrease and outpatient costs to increase due to the intervention. We do not expect Caring Letters to serve as a substitute for any existing mental health outreach program. Costs of both VA-provided and purchased care will be captured. In all analyses, we will use the Gross Domestic Product Deflator to adjust costs to 2020 dollars. The budget impact of each version of Caring Letters will be assessed. Mean costs per patient-month alive will be reported.

Addendum (Covid threats)

Letters began to be mailed out to VCL callers in June 2020, only 3 months after the start of COVID-19-related lockdowns. Therefore, it will be necessary to separate out changes in outcomes that are due to calls during the pandemic from changes in outcomes that are due to receipt of the letters. To isolate the effect of the letters from the effect of the pandemic, we will use a differences in differences-in-differences (or 'triple differences') approach: we will compare changes from before to after a VCL call among a) Veterans who received letters (called between 6/20 and 6/21), b) Veterans who did not receive letters but called during the beginning of COVID lockdowns (called between 3/20 and 5/20), and c) Veterans who did not receive letters and called a year before COVID lockdowns (allowing us to observe outcomes in a pre-pandemic period; called between 6/18 and 3/19). Sensitivity of results to inclusion of a fourth group (those who called between 4/19 and 2/20 – who had part of their post-call period during the beginning of COVID) will be evaluated.

The treatment group studied for this evaluation will have received letters during the COVID-19 pandemic. If the reasons for calling the VCL differ systematically during the pandemic compared to pre-pandemic times, and if these reasons are associated with either baseline risks of outcomes or interact with the caring letters program to change the treatment effect of the program our results may not generalize to a post-pandemic period. To understand the degree to which this is likely to be an issue, we will re-run our analyses, restricting our treatment group to include individuals who received letters but who may have been less adversely affected by the COVID-19 pandemic (e.g., callers from counties with relatively low COVID burden at the time of their call). We can also incorporate measures of COVID burden to the triple differences analyses described above. An additional sensitivity analysis will match callers during the letters campaign to earlier (pre-2020) callers who called from locations experiencing other high-stress events, including wildfires, flooding, and hurricane damage.

Data Flow

VCL Data is downloaded from backups on a weekly basis. The data is downloaded from the Saturday two weeks prior through the end of the Friday of the previous week. That data is then uploaded to the Caring Letters data system that resided on CDWA01. A SQL script is then run to parse the new data, identify callers who can be matched to the CDW and enroll them into the program, then generates a list of all letters due to go out this week.

This includes letters for newly enrolled patients as well as patients enrolled previously who are getting subsequent letters. Once the letter file has been generated it is sent to the print vendor using encrypted email. The print vendor then sends back information on any patients with a change of address, any addresses that are undeliverable and the mail receipt of when each batch of letters was sent out.

Randomized Evaluation of the VCL Caring Letters Suicide Prevention Campaign: Plan for Quantitative Analysis
VERSION 3
Last Updated: June 22, 2023
Evaluation Overview | Quantitative Analysis Overview | Data Flow

Evaluation Overview

Goal: To test the effectiveness of a Caring Letters campaign on clinical outcomes and utilization rates in order to identify an effective and sustainable evidence-based practice to reduce suicide behaviors among veterans.

Eligible Cohort: All VCL callers with an identifiable address in VHA databases. The following callers will be excluded: (1) friends and family calling on behalf of a veteran, (2) civilians, and (3) callers who have died prior to being randomized. Furthermore, individuals who call the hotline after enrollment in the study will be flagged so as not to be re-randomized. Individuals who die after enrollment in the study will be flagged so as not to continue receiving letters.

Treatment/Exposures: A control group will be constructed from VCL callers two years prior to the trial start date. A total of nine (9) letters will be sent to the callers over the course of the intervention. One (1) letter will be sent to all callers on Veteran's Day, and an additional eight (8) letters will be mailed to each caller, each spaced approximately one month apart beginning from the initial call date.[3] Eligible veterans who call the VCL after the trial start date will be randomized to one of two interventions, detailed below.

Arm 1: Peer signatory letter
Arm 2: Provider signatory letter

Randomization: To ensure adequate gender representation in the peer signatory conditions, randomization will be conducted using permuted block randomization, stratified by gender. New callers will be identified monthly; randomization will occur monthly using blocks of 4 patients with 2 conditions (A=peer signatory, B=provider signatory). This set up allows for 6 permutations ((1-AABB, 2-ABAB, 3-ABBA, 4-BAAB, 5-BABA, 6-BBAA)).

After callers are successfully matched to CDW data, they are stratified by gender. Within each stratum, a uniform random number will be generated in SQL (range 0-1) that corresponds to each block of 4 patients. If the random number, R, is >= 0 and < 1/6, the block of 4 callers is assigned to permutation 1 (order of assignment to letter type is AABB). If 1/6 <= R < 2/6, the

[3] The specific months will be 1,2,3,4,6,8,10,12 + Veteran's Day card

block of 4 callers is assigned to permutation 2 (order of assignment to letter type is ABAB). The same pattern follows for permutations 3-6.

Data: The analytic file will be compiled from the following data below. Specific variables are detailed in the sections that follow.

- VCL Medora data
- CDW database
- SSA Vital Status file
- Mortality Data Repository
- OMHSP Standard Suicide Overdose Event Table (encompassing both SPAN and its replacement – the Suicide Behavior and Overdose Report [SBOR])

Outcomes: The outcomes for the study are listed below, along with data source, in Table 1.

Table 1. Study Outcomes

**Clinical Outcomes**	**VHA Utilization (Incidence & Frequency)**
• All-cause mortality (*CDW Spatient Table*, *Vital Status file*)<br>• Suicide mortality (*Mortality data repository*) – left for future analyses<br>• Suicide attempt (incidence (*ICD-10 codes and OMHSP_Standard_SuicideOverdoseEvent Table*)	• Any VA healthcare services (*CDW*)<br>• Psychiatric hospitalization (*CDW*)<br>• Outpatient mental health services (*CDW*)<br>• ED visits (*CDW*)

Additional variables (covariates):

- Sociodemographics
  - Age
  - Gender
  - Race/ethnicity
  - Marital status
  - Branch of military
  - Age at separation
  - Discharge status
  - VAMC
- Comorbidities in past two years
  - Mental illness diagnoses

    - Elixhauser comorbidities
- Past SAEs
- Past mental health utilization

Quantitative Analysis Overview

The primary analyses will be performed after the final letters are sent, and after monitoring and cleaning of data collected from all callers. Main analyses will be performed according to the intention-to-treat (ITT) principle, in which all callers are analyzed according to their initially assigned study arm at baseline, regardless of whether letters were received.

Data structure: Patient-month level.

Analysis 1: Pre-Post Comparison: The pre-post analysis will compare outcomes between callers who receive Caring Letters compared to callers in the previous two years. We will test the following three hypotheses:

- H1: lower incidence of VA documented suicide attempts in the post period.
- H2: lower incidence and frequency of VA psychiatric hospitalization in the post period.
- H3: higher rates of engagement in VA healthcare (in general) and higher outpatient mental health utilization rates in the post period.

We will analyze differences in proportions of VCL callers who engaged with any outpatient VA health care before and after the Caring Letters campaign with a chi-square test. Similarly, chi-square tests will be used to examine differences in incidence of suicide attempts, incidence of psychiatric hospitalization, and incidence of mental health outpatient visits. Differences in frequency of hospitalizations and mental health outpatient visits will be examined with Wilcoxon rank-sum tests.

Pre-post analyses of associations among outcomes and Caring Letters will not be able to rule out the possibility that observed differences in outcomes are due to unmeasured temporal changes coinciding with Caring Letters. One strategy to account for this is a difference in difference-in-differences (triple differences approach). To isolate the effect of the letters from the effect of the pandemic, we attempted to compare changes from before to after a VCL call among a) Veterans who received letters (called between 6/20 and 6/21), b) Veterans who did not receive letters but called during the beginning of COVID lockdowns (called between 3/20 and 5/20), and c) Veterans who did not receive letters and called a year before COVID lockdowns (allowing us to observe outcomes in a pre-pandemic period; called between 6/18 and 3/19). Sensitivity of results to inclusion of a fourth group (those who called between 4/19 and 2/20 – who had part of their post-call period during the beginning of COVID) will be evaluated. A triple differences approach requires an assumption that, absent treatment, trends

in outcomes would evolve in parallel in the post-treatment period. In our sample, trends were not parallel in the pre-treatment period, so the parallel trends assumption was not supported.

An alternate approach models time to event while controlling for time and historic events. In this approach, the cohort was reframed to include index calls between 6/11/2018 and 6/10/2021. Because many VCL callers have more than a single contact with the VCL during their lifetime, we allowed individuals to appear in both the treatment and comparison group. We included all eligible treatment individuals in our cohort who had at least one VA inpatient or outpatient encounter in the 24 months prior to their call. To create the pool of eligible calls for the comparison group, we generated a list of all calls from 6/11/2018 to 6/10/2021 and dropped comparison calls after the index caring letters date for Veterans who are in the treatment group. From the remaining comparison calls that met treatment group eligibility criteria and had at least one VA inpatient or outpatient encounter in the 24 months prior to their call but who did not receive caring letters, we randomly selected one call per Veteran.

We used individual-level survival models for each outcome, modeling time to event. We used cause-specific hazard functions for modeling utilization and suicide attempts, where death was a competing risk. We controlled for COVID (1 = called in March 2020 or later), receipt of caring letters, month, year, as well as the other covariates listed above.

Analysis 2: Comparison of Letter Signatories

Our Aim 2 analyses (cross-arm analyses among individuals engaged in VA care) will not be subject to the history threats to internal validity that exist in Aim 1. We can further isolate the impact of caring letter signatory on outcomes by controlling for the potential confounders noted above, in addition to confounders with potential prognostic value, such as mental health diagnoses (Kahan 2014). Dichotomous outcomes were examined with logistic regression.

Budget Impact Analysis (BIA):

We will examine the budget impact of Caring Letters among all VCL callers over a 12-month period. We will compare costs related to inpatient and outpatient mental health care use and medications before the Caring Letters campaign with costs related to health care use and medications, as well as costs of mailing the letters during the Caring Letters campaign. We expect inpatient costs to decrease and outpatient costs to increase due to the intervention. We do not expect Caring Letters to serve as a substitute for any existing mental health outreach program. Costs of both VA-provided and purchased care will be captured. In all analyses, we will use the Gross Domestic Product Deflator to adjust costs to 2020 dollars. The budget impact of each version of Caring Letters will be assessed. Mean costs per patient-month alive will be reported. This analysis will be completed at a later date.

Data Flow

VCL Data is downloaded from backups on a weekly basis. The data is downloaded from the Saturday two weeks prior through the end of the Friday of the previous week. That data is then uploaded to the Caring Letters data system that resided on CDWA01. A SQL script is then run to parse the new data, identify callers who can be matched to the CDW and enroll them into the program, then generates a list of all letters due to go out this week.

This includes letters for newly enrolled patients as well as patients enrolled previously who are getting subsequent letters. Once the letter file has been generated it is sent to the print vendor using encrypted email. The print vendor then sends back information on any patients with a change of address, any addresses that are undeliverable and the mail receipt of when each batch of letters was sent out.
