# Supplementary material for: Caring Letters Sent by a Clinician or Peer to At-Risk Veterans: A Randomized Clinical Trial
Source: JAMA Netw Open. 2024 Apr 29;7(4):e248064. doi: 10.1001/jamanetworkopen.2024.8064 (PMC11059042; doi:10.1001/jamanetworkopen.2024.8064)
Supplement: Supplement 3. — Data Sharing Statement [file jamanetwopen-e248064-s003.pdf]

Data Sharing Statement

Data
Data available: No

Additional Information
Explanation for why data not available: Data sharing may be possible, but this is usually accomplished through the creation of a de-identified data set and the creation of a Data Use Agreement that complies with all applicable laws and regulations
